# Distribution of *Rhipicephalus microplus* and *Hyalomma lusitanicum,* and the pathogens they are carrying: A systematic review

**DOI:** 10.1016/j.parepi.2025.e00437

**Published:** 2025-06-04

**Authors:** Afito Luciano, Binta J.J. Jallow, Mandie Liu, Yuting Ma, Regina Daniel Miambo, Fanming Meng

**Affiliations:** a School of Basic Medical Sciences, Central South University, Changsha 410013, China; b School of Basic Medical Sciences, Xinjiang Medical University, Urumqi 830017, China; c SCIENCE Department, The Gambia College, Gambia; d Mozambique Institute for Health Education and Research (MIHER), Maputo, Mozambique; e Faculty of Veterinary, Eduardo Mondlane University, Maputo, Mozambique

**Keywords:** *Rhipicephalus microplus*, *Hyalomma lusitanicum*, Tick-borne pathogens, Global

## Abstract

*Rhipicephalus microplus* and *Hyalomma lusitanicum* are highly adaptable ectoparasites that feed on vertebrates, including people and both domestic and wild animals. This systematic review aims to identify, compile, and evaluate relevant articles published after January 1, 2000, until April 30, 2024, from several scientific databases documenting the distribution or prevalence of *Rh*. *microplus* and/or *Hy*. *lusitanicum*, as well as tick-borne pathogens globally. We conducted a thorough search in Embase, Ovid MEDLINE, ScienceDirect, Web of Science, and Scopus from January 1, 2000, to April 30, 2024. This systematic review was implemented according to PRISMA 2020 guidelines. Of the 223 studies included in this systematic review, 83.0 % detected *Rh*. *microplus*, reported across 42 countries. In contrast, 17.0 % detected *Hy*. *lusitanicum*, which has only been reported in eight countries. A total of 113 studies included in this systematic review reported the presence of tick-borne pathogens, with 78.8 % focused on *Rh*. *microplus* and 21.2 % addressing *Hy*. *lusitanicum*. In this review, 94 tick-borne pathogens were reported. Of the tick-borne pathogens identified in *Rh*. *microplus*, bacteria were the most reported, representing 71.6 %, followed by viruses with 15.1 %. Among bacteria, the genus *Anaplasma* was the most frequent, with 26.8 %, followed by *Rickettsia*, with 17.2 %. The tick-borne pathogens identified in *Hy*. *lusitanicum*, bacteria were the most frequent, with 68.1 %, followed by protozoa, with 21.3 %. Genus *Rickettsia* was the most frequent among bacteria, with 25.5 %, followed by *Anaplasma* with 19.2 %. This systematic review provided insight crucial for managing and controlling tick-borne diseases by integrating the One Health approach.

## Highlights

• *Rh. microplus* has a worldwide distribution and harbors a wide range of tick-borne pathogens.
• Cattle are the preferred hosts for *Rh. microplus*, while deer are the preferred hosts for *Hy. lusitanicum.*
• Standard PCR is the most used technique to identify pathogens in *Rh. microplus* and *Hy. lusitanicum.*
• Bacteria are the most frequent pathogens in *Rh. microplus* and *Hy. lusitanicum.*

## Graphical abstract

**Figure:**
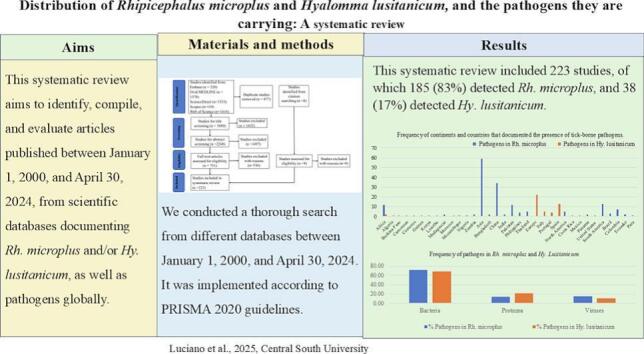

## 1 Introduction

Ticks are hematophagous arthropods that parasitize vertebrates, including humans and wild and domestic animals. Ticks are the world's leading cattle disease vector and rank second in terms of human pathogen transmission (Sultan et al., 2022; Tawiah-Mensah et al., 2024; Díaz-Cao et al., 2022). They can directly cause toxicosis, stress, weakness, skin damage, allergy, anemia, and immune suppression in their hosts (Etiang et al., 2024; Ghafar et al., 2020a; Rafiq et al., 2017; Jongejan and Uilenberg, 2004). Furthermore, they carry a wide variety of pathogens that infect both humans and animals (Tawiah-Mensah et al., 2024; Lu et al., 2022a; Qi et al., 2022; Sousa et al., 2024; Weaver et al., 2021; Zhang et al., 2022a; Blanda et al., 2017; Aubry et al., 2016; Chisu et al., 2019). Every year, ticks are responsible for more than 100,000 human cases of illness worldwide (Lu et al., 2022b). Moreover, 80 % of the world's cattle population is affected by ticks, which have a significant financial impact on livestock because of morbidity and mortality (Hussain et al., 2023; Vieira Lista et al., 2022; Magesa et al., 2023; Jabbar et al., 2015). They are projected to cost between USD 14 billion and USD 19 billion annually, leading to a significant impact on the veterinary sector (Vieira Lista et al., 2022; Balasubramanian et al., 2019). As several tick-borne diseases can harm both people and animals, their emergence and resurgence are raising concerns about global public health (Ali et al., 2019).

*Rhipicephalus microplus* is a highly invasive and adaptable ectoparasite that has spread worldwide. Its notorious nature results from its ability to spread a wide variety of infectious agents, replace other tick species, and acquire resistance to chemicals (Etiang et al., 2024; Addo et al., 2023; Kanduma et al., 2020; Madder et al., 2012; Sungirai et al., 2017; Silatsa et al., 2019a). This tick is characterized by a complex life cycle, including four stages: eggs, larvae, nymphs, and adults. This monotropic species feeds on a single host. The three active forms take approximately three weeks to complete on the host, while almost four weeks for the egg-laying process. The primary hosts of this tick are cattle, buffalo, goats, dogs, sheep, deer, and horses. It can also feed on humans, cats, donkeys, monkeys, and other animals (Ali et al., 2019; Elango et al., 2024; Karim et al., 2017; Khan et al., 2022; Khan et al., 2023; Mahlobo-Shwabede et al., 2021; Misra et al., 2021; Shahid et al., 2021; Alam et al., 2022; Molina-Hoyos et al., 2024). Furthermore, this tick serves as a vector reservoir for several emerging and re-emerging infectious pathogens with importance for human and animal health. These pathogens include bacteria such as *Anaplasma marginale*, *Anaplasma phagocitophylum*, *Borrelia theileri*, *Borrelia garinii*, *Coxiella burnetii*, *Ehrlichia canis*, *Ehrlichia chaffeensis*, *Rickettsia africae*, protozoa such as *Babesia bigemina*, *Theileria orientalis*, and viruses such as severe fever with thrombocytopenia virus (Khan et al., 2023; Xu et al., 2023; Zhang et al., 2022b; Del Valle-Mendoza et al., 2018; Galay et al., 2020; Oundo et al., 2024; Troyo et al., 2016; Phiri et al., 2023; Yssouf et al., 2014; Chiang et al., 2024; Li et al., 2020a; Sumrandee et al., 2015). As for the distribution pattern, this tick is found in Africa, North America, South America, and Asia (Molina-Hoyos et al., 2024; Sumrandee et al., 2015; Matsimbe et al., 2017; Ngnindji-Youdje et al., 2022; Molina-Garza et al., 2024; Cleveland et al., 2019; Szabó et al., 2019; Tsai et al., 2011; Ghafar et al., 2020b; Rialch et al., 2022).

*Hyalomma lusitanicum* is a highly adaptable ectoparasite that can survive in rough environments with few sources of nutrition or during periods of globally extreme environmental change. This ability may play an essential role in its extensive spread, which has been linked to severe global veterinary and public health issues (Valcarcel et al., 2023). *Hyalomma lusitanicum* is characterized by a complex life cycle that includes four stages: eggs, larvae, nymphs, and the adult form. This tick exhibits teletropic behavior and has three hosts. After each feeding session, immature ticks leave their hosts to undergo molting, and female adults leave to deposit eggs. The time spent by the three active stages until egg laying is fourteen to twenty-eight weeks under controlled conditions (Valcarcel et al., 2023; Valcárcel et al., 2020; Walker, 2003; Apanaskevich et al., 2008). *Hyalomma lusitanicum* hosts include cattle, deer, boars, rabbits, goats, birds, dogs, and horses. It occasionally bites humans and other animals (Apanaskevich et al., 2008; Elhachimi et al., 2021; Negredo et al., 2019; Paz Sanchez-Seco et al., 2022; Mokhtaria et al., 2018; Jimale et al., 2024). Furthermore, this tick harbors many pathogens, such as bacteria, including *Anaplasma marginale*, *Bartonella* spp., *Borrelia lusitaniae*, *Coxiella burnetii*, *Ehrlichia minasensis*, *Rickettsia massiliae*, protozoa such as *Babesia occultans*, *Babesia microti*, *Theileria orientalis*, and viruses such as Crimean–Congo hemorrhagic fever virus (Elhachimi et al., 2021; Negredo et al., 2019; Milhano et al., 2010; Gonzalez et al., 2020; Remesar et al., 2022). In addition, this tick is found in Europe and Africa (Elhachimi et al., 2021; Cajimat et al., 2017; Santos-Silva et al., 2017; Boularias et al., 2021).

Recently, ticks and tick-borne diseases have expanded to wider geographical areas due to many factors, such as climate change, urbanization, deforestation, animal movements, and increased global trade and travel (Blanda et al., 2017; Aubry et al., 2016; Oundo et al., 2024; Boularias et al., 2021; Xiang et al., 2023; Zhao et al., 2023; Fernández et al., 2022). This has resulted in changing rates of emerging and re-emerging diseases as well as tick-borne pathogens. These two selected ticks are highly impacted by the factors mentioned earlier, in addition to their significant medical and veterinary value (Addo et al., 2023; Valcarcel et al., 2023; Requena-García et al., 2017; Silatsa et al., 2019b; Nuttall, 2021). Some studies and reviews have documented the distribution of *Rhipicephalus* spp. or *Hy*. *lusitanicum* and the tick-borne pathogens they harbor in some parts of the world (Valcarcel et al., 2023; Mucheka et al., 2023; Van Wyk et al., 2023). To the best of our knowledge, systematic reviews on the distribution of *Rh*. *microplus*, and *Hy*. *lusitanicum* ticks worldwide are limited. Given the increasing factors that influence the spread of these two ticks to new areas and their significant impact on human and animal health globally, studies on the distribution of *Rh*. *microplus* and/or *Hy*. *lusitanicum*, as well as the tick-borne pathogens that they harbor, by integrating the One Health perspective, should continue. This could help identify the risky areas and develop strategies for managing and controlling tick-borne diseases (Valcarcel et al., 2023; Kobayashi et al., 2021). Thus, this systematic review aims to identify, compile, and evaluate articles published between 2000 and 2024 from several scientific databases documenting the distribution and prevalence of *Rh*. *microplus* and *Hy*. *lusitanicum* and their associated tick-borne pathogens globally.

## 2 Materials and methods

### 2.1 Search strategy and selection criteria

**Fig. 1:**
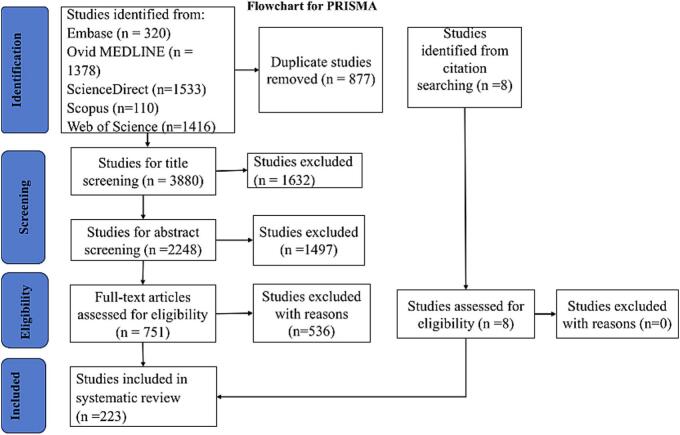
Flowchart for the selection of studies according to PRISMA 2020 recommendations.

A systematic literature search was performed in pairs according to the Preferred Reporting Items for Systematic Reviews and Meta-Analyses (PRISMA) criteria (Fig. 1). The search was implemented in Embase, Ovid MEDLINE, ScienceDirect, Web of Science, and Scopus using the key terms “tick,” “*Rhipicephalus microplus*,” “*Hyalomma lusitanicum*,” Pathogens.” In addition, Boolean operators “AND” and “OR” were used to combine key terms. To identify studies that were not found in the database search, we conducted a manual search using the references of retrieved articles. Moreover, the search focused on studies worldwide published between January 1, 2000, and April 30, 2024. Therefore, the following combined key terms were used in the search.

1. For Embase, the search terms were Ticks AND *Rhipicephalus microplus* OR *Hyalomma lusitanicum* AND Pathogens.
2. For Ovid MEDLINE, the search terms were Ticks AND *Rhipicephalus microplus* OR *Hyalomma lusitanicum* AND Pathogens.
3. For ScienceDirect, the search terms were Ticks AND *Rhipicephalus microplus* OR *Hyalomma lusitanicum* AND Pathogens.
4. For Web of Science, the search terms were Ticks AND *Rhipicephalus microplus* OR *Hyalomma lusitanicum* AND Pathogens.
5. For Scopus, the search terms were Ticks AND *Rhipicephalus microplus* OR *Hyalomma lusitanicum* AND Pathogens.
  Potential articles were selected by removing duplicates, screening titles and relevance, and screening abstracts. The selected studies were downloaded so that the full text could be reviewed for eligibility. EndNote V21 was used to store and manage full-text articles.

### 2.2 Inclusion and exclusion criteria

The inclusion criteria for this review were: (Sultan et al., 2022) original articles; (Tawiah-Mensah et al., 2024) open access articles; (Díaz-Cao et al., 2022) studies published in English; (Etiang et al., 2024) studies published between January 1, 2000, and April 30, 2024; (Ghafar et al., 2020a) studies that identified *Rh*. *microplus* and/or *Hy*. *lusitanicum*; (Rafiq et al., 2017) studies that identified ticks at the species level; (Jongejan and Uilenberg, 2004) studies that specified the number of ticks collected; (Lu et al., 2022a) studies that specified hosts; and (Qi et al., 2022) studies that specified the study location. The exclusion criteria were as follows: (Sultan et al., 2022) review of studies and (Tawiah-Mensah et al., 2024) studies that did not meet the inclusion criteria.

### 2.3 Data extraction

A spreadsheet was created to record the data collected from the articles, and the studies that met the inclusion criteria were recorded. The following information was recorded on the spreadsheet: country of study, host type, type of tick species, total number of ticks collected, diagnostic method used, type of tick-borne pathogens present, diagnostic method of pathogens, and study references.

### 2.4 Quality assessment

The risk of bias was assessed using the Joanna Briggs Institute (JBI) critical appraisal tool checklist for studies reporting prevalence. This tool assesses each study according to nine criteria (Munn et al., 2015). In this study, eight criteria were applied, and the last question was not applicable. A score of 6 or above indicated a high-quality study, whereas a score of less than 6 indicated a low-quality. Two reviewers independently assessed the selected articles, and discrepancies were resolved by discussion or by a third reviewer. The assessment of the quality of selected studies is presented in Supplementary Table 3.

## 3 Results

The systematic search yielded 4757 studies from five databases. After removing 877 duplicates, 3880 studies were retained. Title screening excluded 1632 studies. The abstracts of the remaining 2248 studies were screened, leading to the exclusion of 1497 articles. The remaining 751 studies were submitted for eligibility by a full-text evaluation, of which 536 were excluded. A total of 215 studies that met the inclusion criteria were included. Eight additional studies were obtained from the reference lists of articles that were not initially retrieved and were subsequently examined for eligibility by full-text screening. Thus, all eight studies met the inclusion criteria, with a total of 223 studies for qualitative analyses. The representative search and number of eligible studies are presented in Fig. 1.

### 3.1 Characteristics of the studies included in the systematic review

The characteristics of the studies on the distribution of *Rh. microplus* and *Hy*. *lusitanicum* are summarized in Supplementary Table 1. These included the country where the study was carried out, the host type that was sampled, the presence or number of *Rh*. *microplus* and/or *Hy*. *lusitanicum*, the total number of ticks collected, the diagnostic method of ticks, and study references. The characteristics of the studies related to the presence of tick-borne pathogens in *Rh*. *microplus* and/or *Hy*. *lusitanicum* are summarized in Supplementary Table 2. We included the country where the study was carried out, the presence of tick-borne pathogens in *Rh*. *microplus* and/or *Hy*. *lusitanicum*, the diagnostic method for tick-borne pathogens, and study references.

### 3.2 Distribution of *Rh*. *microplus* and/or *Hy*. *lusitanicum*

**Fig. 2:**
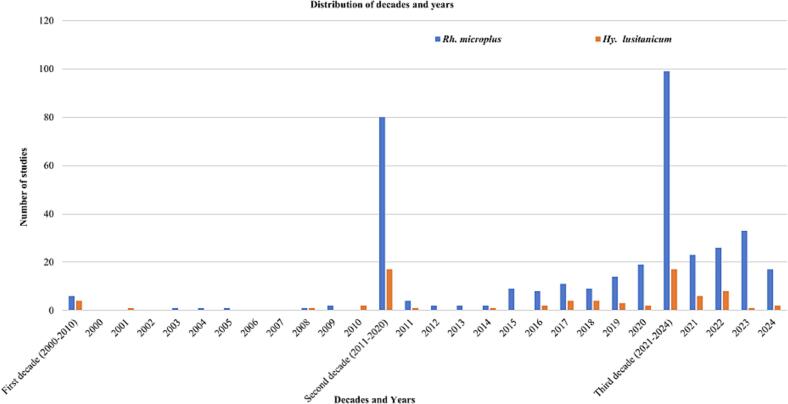
Number of publications of studies reporting *Rh*. *microplus* and/or *Hy*. *lusitanicum* through the years and decades.

Out of a total of 223 studies included in this systematic review, 185 (82.96 %) detected *Rh*. *microplus*, and 38 (17.04 %) detected *Hy*. *lusitanicum*. The studies that reported *Rh*. *microplus*, 99 (53.51 %) were published in the third decade, from 2021 to 2024 (2023 (*n* = 33), 2022 (*n* = 26), 2021 (*n* = 23), and 2024 (*n* = 17)); 80 (43.24 %) were published in the second decade, from 2011 to 2020 (2020 (*n* = 19), 2019 (*n* = 14), 2017 (*n* = 11), 2015 and 2018 (*n* = 9), 2016 (*n* = 8), 2011 (*n* = 4), 2012, 2013, and 2014 (*n* = 2)); 6 (3.24 %) were published in the first decade, from 2000 to 2010 (2009 (*n* = 2), 2003, 2004, 2005, and 2008 (*n* = 1)) (Fig. 2). While studies detected *Hy*. *lusitanicum* 17 (44.74 %) were published in the third decade (2022 (n = 8), 2021 (*n* = 6), 2024 (*n* = 2), 2023 (*n* = 1)) and the second decade (2017 and 2018 (*n* = 4), 2019 (*n* = 3), 2016 and 2020 (n = 2), 2011 and 2014 (n = 1)); 4 (10.53 %) were published in the first decade (2010 (n = 2), 2001 and 2008 (n = 1)) (Fig. 2).

**Fig. 3:**
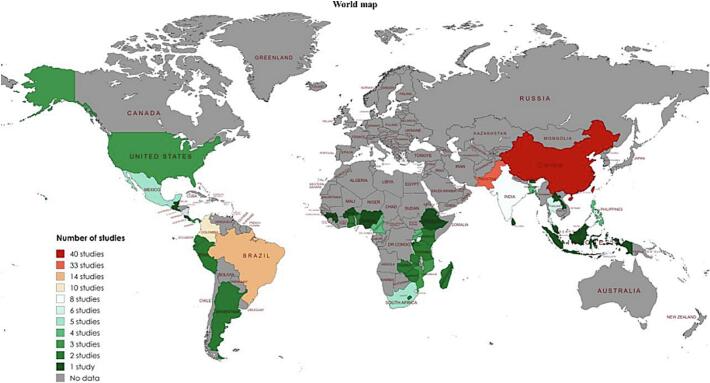
Global map indicating the number of studies from various countries that have documented the presence of *Rh*. *microplus*.

**Fig. 4:**
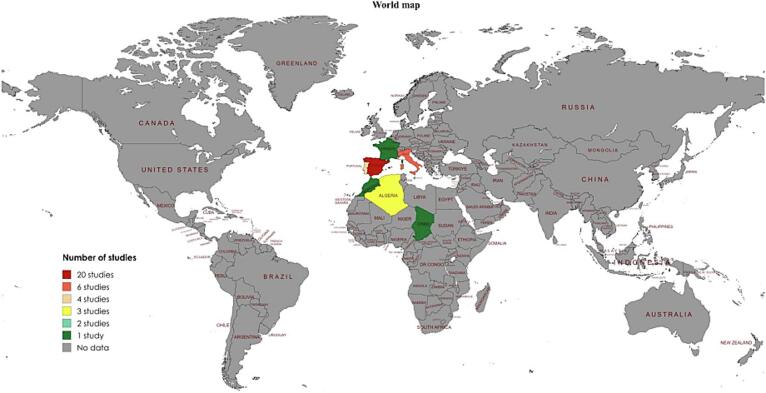
Global map indicating the number of studies from different countries that documented the presence of *Hy*. *lusitanicum*.

The studies that identified *Rh*. *microplus* 100 (54.05 %) were from Asia (China (*n* = 40), Pakistan (*n* = 33), India (*n* = 8), Thailand (*n* = 6), Bangladesh and Philippines (n = 4), Bhutan, Indonesia, Laos, Malaysia and Sri Lanka (*n* = 1)); 39 (21.08 %) were from Africa (South Africa (*n* = 5), Cameroon and Uganda (n = 4), Mozambique (*n* = 3), Benin, Comoros, Ghana, Kenya, Lesotho, Madagascar, Tanzania, Zambia and Zimbabwe (*n* = 2), Burkina Faso, Burundi, Ethiopia, Guinea and Nigeria (*n* = 1)); 30 (16.22 %) were from South America (Brazil (*n* = 14), Colombia (*n* = 10), Argentina, Ecuador and Peru (*n* = 2)); 16 (8.65 %) were from North America (Mexico (n = 5), Panama and United States (*n* = 3), Belize, Costa Rica, El Salvador, Guatemala and Trinidad and Tobago (*n* = 1)) (Fig. 3). While studies detected *Hy*. *lusitanicum* 33 (86.84 %) were from Europe (Spain (*n* = 20), Italy (*n* = 6), Portugal (*n* = 4), Malta (n = 2) and France (n = 1)); 5 (13.16 %) were from Africa (Algeria (n = 3), Chad and Morocco (n = 1) (Fig. 4).

**Fig. 5:**
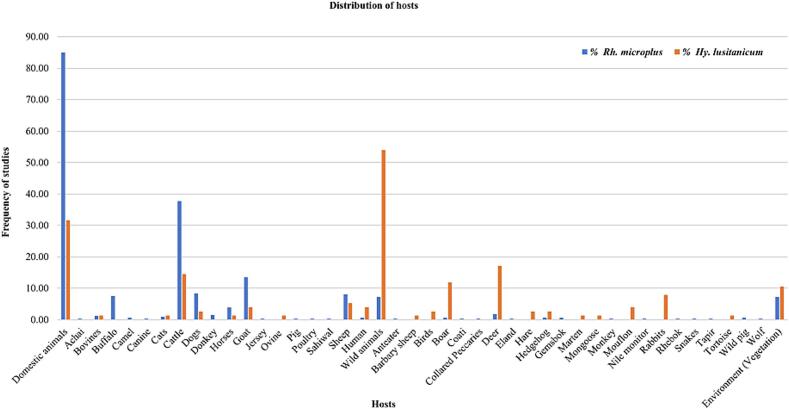
Frequency of hosts of *Rh*. *microplus* and *Hy*. *lusitanicum*.

The studies included in this systematic review were collected from 42 hosts. Studies identified *Rh*. *microplus*, 85.03 %, were collected from domestic animals (cattle (37.72 %), goats (13.47 %), (dogs 8.38 %), sheep (8.08 %), buffalo (7.49 %), horses (3.89 %), donkeys (1.50 %), bovines, (1.20 %), cats (0.90 %), camel (0.60 %), Achai, canine, Jersey, pig, Sahiwal, and poultry (0.30 %)); 7.19 % from wild animals (deer (1.80 %), hedgehog, gemsbok, wild pig, boar (0.60 %), anteater, coati, collared peccaries, eland, monkey, Nile monitor, rhebok, snakes, tapir, and wolf (0.30 %)); 7.19 % from the environment (vegetation) and 0.60 % from humans (Fig. 5).

The studies reported *Hy*. *lusitanicum*, 53.95 %, were collected from wild animals (deer (17.11 %), boar (11.84 %), rabbits (7.89 %), mouflon (3.95 %), birds, hare, hedgehog (2.63 %), barbary sheep, marten, mongoose, and the tortoise (1.32 %)); 31.58 % from domestic animals (cattle (14.47 %), sheep (5.26 %), goat (3.95 %), dogs (2.63 %), bovines, cats, horses, ovine (1.32 %)); 10.53 % from the environment (vegetation) and 3.95 % from humans (Fig. 5).

**Fig. 6:**
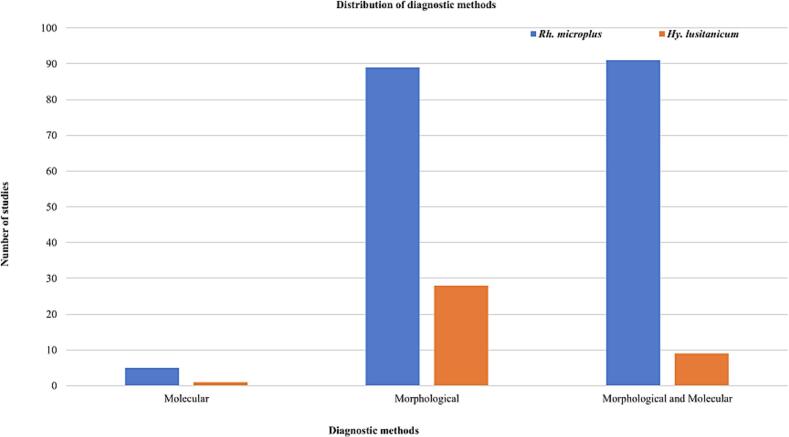
Useful frequency of diagnostic methods of *Rh*. *microplus*, and/or *Hy*. *lusitanicum*.

The diagnostic methods used for the detection of *Rh*. *microplus* in this systematic review were molecular, morphological, and a combination of both. Five studies used molecular techniques, 89 used morphological techniques, and 91 used a combination of morphological and molecular methods. The techniques used to identify *Hy*. *lusitanicum* were molecular (*n* = 1), morphological (*n* = 28), or a combination of morphological and molecular techniques (*n* = 9) (Fig. 6).

### 3.3 Distribution of tick-borne pathogens detected in *Rh*. *microplus*, or *Hy*. *lusitanicum*

**Fig. 7:**
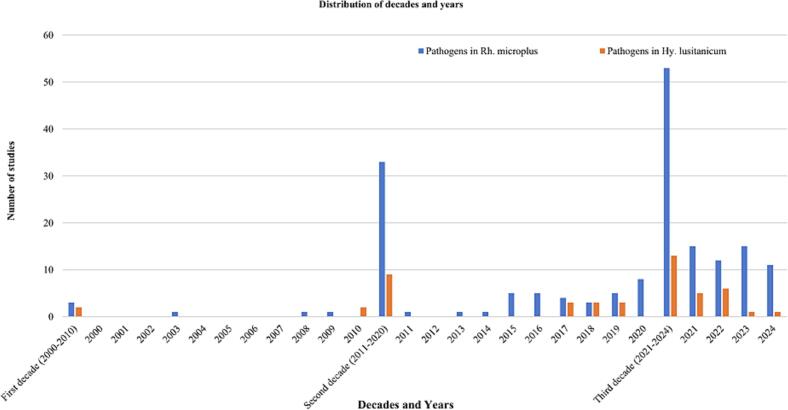
Number of studies reporting tick-borne pathogens in *Rh*. *microplus*, and/or *Hy*. *lusitanicum* through the years and decades.

This systematic review included 113 studies that identified tick-borne pathogens in *Rh*. *microplus*, and/or *Hy*. *lusitanicum*. Eighty-nine studies (78.76 %) reported the presence of tick-borne pathogens in *Rh*. *microplus*, whereas 24 (21.24 %) reported the presence of tick-borne pathogens in *Hy*. *lusitanicum*. Reported tick-borne pathogens within *Rh*. *microplus* 53 (59.55 %) studies were published in the third decade (2021 and 2023 (*n* = 15), 2022 (*n* = 12), 2024 (*n* = 11)); 33 (37.08 %) were published in the second decade (2020 (*n* = 8), 2015, 2016, and 2019 (*n* = 5), 2017 (*n* = 4), 2018 (*n* = 3), 2011, 2013, and 2014 (n = 1)); 3 (3.37 %) were published in the first decade (2003, 2008, and 2009 (n = 1)) (Fig. 7). The tick-borne pathogens reported in *Hy*. *lusitanicum* 13 (54.17 %) studies were published in the third decade (2022 (*n* = 6), 2021 (n = 5), 2023 and 2024 (*n* = 1)); 9 (37.50 %) were published in the second decade (2017, 2018, and 2019 (*n* = 3)); 2 (8.33 %) were published in the first decade (2010 (*n* = 2)) (Fig. 7).

**Fig. 8:**
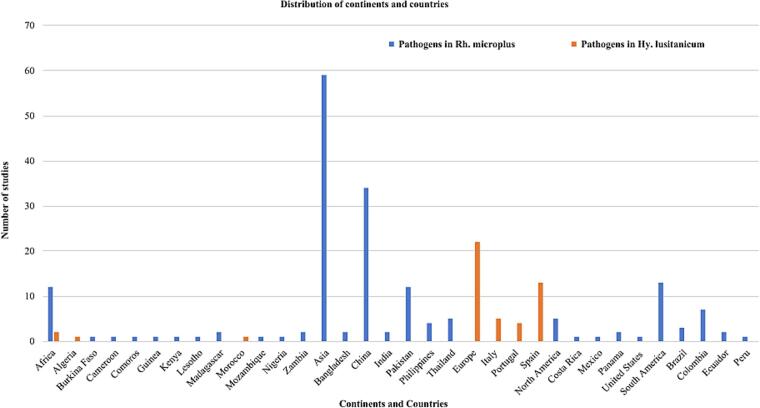
Frequency of continents and countries that documented the presence of tick-borne pathogens carried by *Rh*. *microplus* and/or *Hy*. *lusitanicum*.

Identified tick-borne pathogens within *Rh*. *microplus* 59 (66.29 %) studies were from Asia (China (*n* = 34), Pakistan (*n* = 12), Thailand and the Philippines (*n* = 4), Bangladesh and India (n = 2)); 13(14.61 %) were from South America (Colombia (*n* = 7), Brazil (n = 3), Ecuador (n = 2) and Peru (n = 1)); 12 (13.48 %) were from Africa (Madagascar and Zambia (n = 2), Burkina Faso, Cameroon, Comoros, Guinea, Kenya, Lesotho, Mozambique, Nigeria (n = 1)); 5 (5.62 %) were from North America (Panama (n = 2), Costa Rica, Mexico and the United States (n = 1)) (Fig. 8). The tick-borne pathogens identified in *Hy*. *lusitanicum* 22 (91.67 %) studies were from Europe (Spain (*n* = 13), Italy (*n* = 5), Portugal (n = 4)); 2 (8.33 %) were from Africa (Algeria and Morocco (n = 1)) (Fig. 8).

**Fig. 9:**
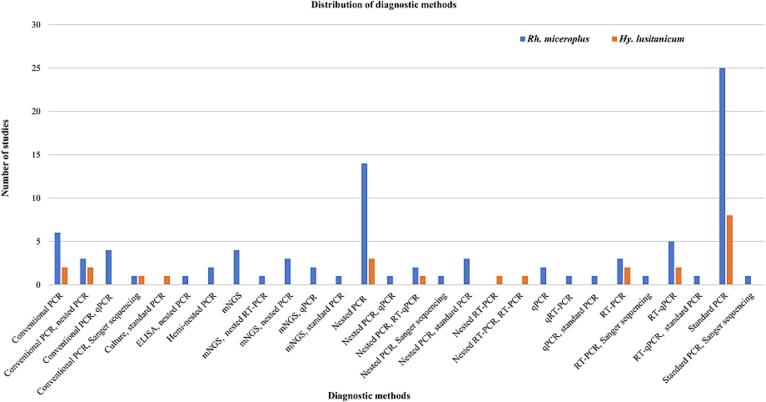
Frequency of diagnostic methods for tick-borne pathogens carried by *Rh*. *microplus*, and/or *Hy*. *lusitanicum*.

In this study, different techniques were used to detect pathogens in *Rh*. *microplus*, including molecular, serological, culture, and a combination of both. Twenty-five studies used standard PCR assays, 14 studies used nested PCR assays, 6 studies used conventional PCR assays, 5 studies used RT-qPCR assays, 4 studies used a combination of conventional PCR and qPCR; 4 studies used mNGS; 3 studies used a combination of conventional PCR and nested PCR, 3 studies used a combination of mNGS and nested PCR, 3 studies used a combination of nested PCR and standard PCR, 3 studies used RT-PCR, 2 studies used heminested PCR, 2 studies used a combination of mNGS and qPCR, 2 studies used a combination of nested PCR and RT-qPCR, two studies used qPCR, one study used a combination of conventional PCR and Sanger sequencing, one study used a combination of ELISA and nested PCR, one study used a combination of mNGS and nested RT-PCR, one study used a combination of mNGS and standard PCR, one study used a combination of nested PCR and qPCR, one study used a combination of nested PCR, Sanger sequencing, one study used qRT-PCR, one study used a combination of qPCR and standard PCR, one study used a combination of RT-PCR and Sanger sequencing, one study used a combination of RT-qPCR and standard PCR, and one study used a combination of standard PCR and Sanger sequencing. While for the identification of tick-borne pathogens in *Hy*. *lusitanicum*, were used standard PCR (*n* = 8), nested PCR (*n* = 3), conventional PCR (*n* = 2), a combination of conventional PCR and nested PCR (n = 2), RT-PCR (n = 2), RT-qPCR (n = 2), a combination of conventional PCR and Sanger sequencing (*n* = 1), a combination of culture and standard PCR (n = 1), a combination of conventional nested PCR and RT-qPCR (n = 1), nested RT-PCR (n = 1), a combination of nested RT-PCR and RT-PCR (n = 1) (Fig. 9).

The studies included in this systematic review identified 94 tick-borne pathogens. The studies identified tick-borne pathogens in *Rh*. *microplus* comprised 71.55 % bacteria, 15.06 % viruses, and 13.39 % protozoa. Among bacteria, 26.78 % belong to the genus *Anaplasma*, 17.15 % to *Rickettsia*, 13.81 % to *Ehrlichia*, 4.60 % to *Coxiella*, 3.77 % to *Bartonella* and *Borrelia*, 0.84 % to *Acinetobacter*, 0.42 % to *Dermatophilus* and *Mycobacterium*, among protozoa 6.69 % belong to the genus *Theileria*, 5.44 % to *Babesia* and 1.26 % to *Hepatozoon*, among viruses 4.60 % belong to the genus *Flavivirus*, 2.51 % to *Uukuvirus*, 1.67 % to *Orthonairovirus*, 0.84 % to *Phlebovirus*, *Bandavirus*, *Mivirus*, *Quaranjavirus* and *Timovirus*, 0.42 % to *Norwavirus*, *Hepacivirus*, *Parapoxvirus*, *Orthopoxvirus* and *Rhabdovirus* (Supplementary Table 4).

Studies identified tick-borne pathogens in *Hy*. *lusitanicum*, comprising 68.09 % bacteria, 21.28 % protozoa, and 10.64 % viruses. Among bacteria, 25.53 % belong to the genus *Rickettsia*, 19.15 % to *Anaplasma*, 8.51 % to *Ehrlichia*, 6.38 % to *Coxiella*, 4.26 % to *Borrelia*, and 2.13 % to *Bartonella* and *Francisella*, among protozoa, 10.64 % belong to the genus *Babesia* and *Theileria* and among viruses, 8.51 % belong to the genus *Orthonairovirus* and 2.13 % to the *Paslahepevirus* (Supplementary Table 4).

## 4 Discussion

### 4.1 Distribution of *Rhipicephalus microplus* and/or *Hyalomma lusitanicum*

This systematic review assessed 223 studies, of which *Rh*. *microplus* was the most frequently reported (82.96 %), and a few, 17.04 %, reported *Hy*. *lusitanicum*.

The majority of studies that identified *Rh. microplus* 99 (53.51 %) were published in the third decade, from 2021 to 2024, followed by the second decade, 80 (43.24 %), from 2011 to 2020. The majority of studies that reported *Hy*. *lusitanicum* 17 (44.74 %) were published in the third decade and the second decade. The number of studies on both ticks has been increasing in recent decades. In 2024, the number of studies declined. This may be because we completed the search on April 30, 2024. The increase in studies of these two ticks can be explained by the increase in pathogens that are important in public health and the veterinary sector (Vieira Lista et al., 2022; Ali et al., 2019).

*Rhipicephalus microplus* has been reported worldwide, including Asia, South America, North America, and Africa, whereas *Hy*. *lusitanicum* has only been reported in Europe and Africa. *Rhipicephalus microplus* was reported in 42 countries, whereas *Hy*. *lusitanicum* was reported in only eight countries. The majority of studies included in this systematic review reported *Rh*. *microplus* 54.05 % were from Asia (China (*n* = 40), and Pakistan (*n* = 33), while the least frequent 8.65 % were from North America (Belize, Costa Rica, El Salvador, Guatemala, and Trinidad and Tobago (*n* = 1)). Furthermore, the most frequently reported *Hy*. *lusitanicum* 86.84 % studies were from Europe (Spain (*n* = 20), while the least frequently reported 13.16 % were from Africa (Chad and Morocco (n = 1)). *Rhipicephalus microplus* originates in the Southeastern region of Asia but has expanded to other parts of the world (Walker, 2003; Horak et al., 2015). While *Hy*. *lusitanicum* is abundant in the western region of the Mediterranean subregion of European countries, it has expanded to other parts of Europe and some parts of Africa (Valcarcel et al., 2023; Valcárcel et al., 2020; Walker, 2003; Perveen et al., 2021). This distribution pattern and frequency of both *Rh*. *microplus* and *Hy*. *lusitanicum* ticks in the new areas are increasing due to many factors, including the movement of animals, climatic conditions, vegetation, and their adaptive capacity to the new environment (Vieira Lista et al., 2022; Addo et al., 2023; Madder et al., 2012; Valcárcel et al., 2020; Balinandi et al., 2020; Muhanguzi et al., 2020; Barandika et al., 2011; Zachée et al., 2020). This systematic review included studies on *Rh*. *microplus* collected from various hosts. In most studies, 85.03 % were collected from domestic animals (cattle, goats, dogs, sheep, buffalo). In contrast, the fewest, 0.60 %, were collected from humans. Although it was initially believed that *Rh*. *microplus* was exclusive to bovine hosts, reports have shown that it can parasitize a variety of domestic animals, as well as wildlife (Rodríguez-Vivas et al., 2013). This increase in the type of hosts can be explained not only because they must have acceptance of hosts when they are introduced in a new geographical area to complete the life cycle but also because the ability to adapt to the new environment can facilitate adaptation to the different types of hosts that are available in the new geographical area (Madder et al., 2012; Silatsa et al., 2019b). Studies that have reported *Hy*. *lusitanicum* were collected from various hosts. The majority, 53.95 %, were collected from wild animals (deer, boar, rabbits). In contrast, the least of the studies, 3.95 %, were collected from humans. *Hy*. *lusitanicum* is known to have a variety of hosts for each life cycle, including domestic and wild animals. This tick, because it is a three-host species, is believed to have different hosts during its life cycle (Apanaskevich et al., 2008). However, the type of hosts is also increasing, and many factors mentioned above contribute to its expansion. For example, when climate conditions change, humans destroy the animals' natural habitat or move close to the animals' habitats, automatically the type of host changes, and they must adapt to the new conditions (Nuttall, 2021).

Regarding the diagnostic methods for detecting *Rh*. *microplus* in this systematic review, most studies used a combination of morphological and molecular methods, followed by morphological techniques; the fewest studies used molecular techniques. Morphological identification is insufficient to distinguish between complexes of related species and *Rh*. *microplus*, especially when the specimens are physically damaged, engorged, or at an immature stage. Nonetheless, several studies recommend and use a combination of morphological and molecular approaches (Tawiah-Mensah et al., 2024; Balinandi et al., 2020; Intirach et al., 2023; Kandi et al., 2022; Rooman et al., 2021; Rehman et al., 2017). Furthermore, most of the techniques used to identify *Hy*. *lusitanicum*, were morphological techniques, followed by a combination of morphological and molecular techniques, and the least were molecular techniques. When morphological counterparts are challenging to identify *Hy*. *lusitanicum*, molecular identification is sometimes required. Examples include immature stages, damaged or engorged specimens, and species that share a similar morphology (Valcarcel et al., 2023; Estrada-Peña et al., 2017).

### 4.2 Distribution of tick-borne pathogens detected in *Rh*. *microplus*, or *Hy*. *lusitanicum*

This systematic review included 113 studies that identified tick-borne pathogens. In most studies, 78.76 % reported tick-borne pathogens in *Rh*. *microplus*, while 21.24 % reported them in *Hy*. *lusitanicum*. The majority of the studies reporting tick-borne pathogens in *Rh*. *microplus*, 53 (59.55 %), were published in the third decade, followed by the second decade, 33 (37.08 %). Most studies identified tick-borne pathogens reported in *Hy*. *lusitanicum* 13 (54.17 %) were published in the third decade, followed by the second decade, 9 (37.50 %). The number of studies on tick-borne pathogens carried by these two ticks has also been increasing in recent decades. This may be due to the increasing distribution of these ticks, and the pathogens they transmit are significant to human and animal health (Vieira Lista et al., 2022; Ali et al., 2019). Twenty-four countries reported tick-borne pathogens in *Rh*. *microplus*, and five reported tick-borne pathogens in *Hy*. *lusitanicum*. In most studies that reported tick-borne pathogens in *Rh*. *microplus*, 66.29 % were from Asia (China (*n* = 34)), whereas the least frequent, 5.62 %, were from North America (Costa Rica, Mexico, and the United States (*n* = 1)). In many studies that reported tick-borne pathogens in *Hy*. *lusitanicum*, 91.67 % were from Europe (Spain (*n* = 13), whereas the least frequent were from Africa (Algeria and Morocco (n = 1)). The distribution of tick-borne pathogens is directly linked to the distribution of *Rh*. *microplus*, and *Hy*. *lusitanicum*. It is influenced by several factors, such as climate change, urbanization, deforestation, animal movement, and increased global trade and travel (Díaz-Cao et al., 2022; Sousa et al., 2024; Blanda et al., 2017; Oundo et al., 2024; Boularias et al., 2021; Xiang et al., 2023; Zhao et al., 2023; Hornok et al., 2022; Ehlers et al., 2020; Pereira et al., 2018; Castillo-Contreras et al., 2022). To detect pathogens in both *Rh*. *microplus* and *Hy*. *lusitanicum*, most studies used molecular techniques such as standard PCR assays, nested PCR assays, and conventional PCR assays. Fewer studies employed a combination of different molecular, culture, and serological techniques such as NGS, Sanger sequencing, and ELISA. Molecular techniques have enabled the characterization of species to the species level and have played a significant role in the detection of new tick-borne pathogens that are unable to be isolated by culture-based techniques (Shahid et al., 2021; Molina-Garza et al., 2024; Ghafar et al., 2020b; Elhachimi et al., 2021; Negredo et al., 2019; Milhano et al., 2010; Remesar et al., 2022; Rehman et al., 2019; Lu et al., 2017; Hernandez et al., 2023; Gómez et al., 2020; Intirach et al., 2024; Pesquera et al., 2015; Shi et al., 2021).

The studies included in this systematic review reported a range of potential emerging tick-borne pathogens in *Rh*. *microplus*, and *Hy*. *lusitanicum*. The majority of tick-borne pathogens identified in *Rh*. *microplus*, 71.55 %, were bacteria, while the least 13.39 % were protozoa. Of the majority of tick-borne pathogens identified in *Hy*. *lusitanicum*, 68.09 % were bacteria, while the least 10.64 % were viruses.

#### 4.2.1 Bacteria

##### 4.2.1.1 Anaplasma

*Anaplasma* species are Gram-negative, obligatory intracellular bacteria belonging to the family Anaplasmataceae and the order Rickettsiales*.* They cause various diseases in both humans and animals (Thinnabut et al., 2023; Lu et al., 2022c). This systematic review reported 11.30 % of *A. marginale*, in *Rh*. *microplus* from 11 countries (China, Pakistan, Philippines, Guinea, Colombia, Cameroon, Ecuador, Madagascar, Brazil, Panama, and Thailand) and 2.13 % in *Hy*. *lusitanicum*, from Morocco. *Anaplasma centrale*, 2.93 %, was identified only in *Rh*. *microplus* from 6 countries (Thailand, Pakistan, India, Cameroon, Ecuador, and Philippines). *Anaplasma marginale* and *A. centrale* are Gram-negative bacteria that cause anaplasmosis in ruminants (Zhang et al., 2022b; Matsimbe et al., 2017; Ngnindji-Youdje et al., 2022; Elhachimi et al., 2021; Zhao et al., 2023; Thinnabut et al., 2023; Ali et al., 2023a; Gioia et al., 2018; Galay et al., 2021; Lu et al., 2022d; Pothmann et al., 2016; Polsomboon et al., 2017; Zhang et al., 2023; Chisu et al., 2018a). Anaplasmosis is characterized by progressive hemolytic anemia, condition loss, abortion, altered milk production, and death (Makenov et al., 2021; Ybanez et al., 2013; Bermudez et al., 2024). *Anaplasma bovis*, 0.42 %, was identified in *Rh*. *microplus* from China, and 2.13 % in *Hy*. *lusitanicum* from Morocco. *Anaplasma bovis* is an obligatory intracellular Gram-negative bacterium, thought to cause bovine anaplasmosis in cattle and buffalo (Elhachimi et al., 2021; Rehman et al., 2019; Lu et al., 2017; Thinnabut et al., 2023; Chisu et al., 2018a; Lu et al., 2023; Ghafar et al., 2020c), in addition to causing infection in humans (Zhao et al., 2023; Lu et al., 2022d; Zhang et al., 2023; Lu et al., 2023). *Anaplasma platys*, 4.18 %, was reported in *Rh*. *microplus* from 4 countries (Thailand, China, Guinea, and Cameroon), and 6.38 % was identified in *Hy*. *lusitanicum*, from 3 countries (Morocco, Italy, and Portugal). *Anaplasma platys* is an obligatory intracellular Gram-negative bacterium that causes canine cyclic thrombocytopenia and infections in dogs, cattle, goats, horses, camels, cats, and humans (Elhachimi et al., 2021; Zhao et al., 2023; Kobayashi et al., 2021; Lu et al., 2017; Thinnabut et al., 2023; Lu et al., 2022c; Guo et al., 2019a; Qiu et al., 2016). Typically, it manifests as chills, malaise, headache, fever, and dizziness; these vague symptoms are easily confused with other diseases (Guo et al., 2019a). *Anaplasma capra*, 1.67 %, was identified in *Rh*. *microplus* from Thailand and China, and 2.13 % in *Hy*. *lusitanicum* from Morocco. *Anaplasma capra* is a type of Gram-negative bacterium thought to cause diseases in goats, sheep, cattle, dogs, and wild animals in addition to humans worldwide (Lu et al., 2022b; Elhachimi et al., 2021; Zhang et al., 2023; Lu et al., 2023; Guo et al., 2019a; Qiu et al., 2016; Segura et al., 2024). The aforementioned pathogen, *A. platys*, causes similar clinical symptoms like fever, dizziness, chills, headache, myalgia, malaise, eschar, rash, and lymphadenopathy (Lu et al., 2022a; Zhang et al., 2023; Guo et al., 2019a). *Anaplasma phagocitophylum*, 1.26 %, was identified in *Rh*. *microplus* from Colombia, Ecuador, and China, and 6.38 % in *Hy*. *lusitanicum* from Morocco, Italy, and Spain. *Anaplasma phagocitophylum* is an obligatory intracellular Gram-negative bacterium that not only causes tick-borne fever (TBF) or granulocytic anaplasmosis (GA) in several mammalian species but also causes diseases known as human granulocytic anaplasmosis (HGA), human granulocytic *Ehrlichia*, or *Ehrlichia phagocytophilia* in humans (Lu et al., 2022b; Mahlobo-Shwabede et al., 2021; Zhang et al., 2022b; Elhachimi et al., 2021; Thinnabut et al., 2023; Lu et al., 2022c; Gioia et al., 2018; Lu et al., 2022d; Zhang et al., 2023; Chisu et al., 2018a; Lu et al., 2023; Ghafar et al., 2020c; Guo et al., 2019a; Segura et al., 2024; Sumrandee et al., 2016; Nogueira et al., 2017; Osorio et al., 2018; Parola et al., 2003; Stuen et al., 2013). *Anaplasma ovis*, 2.51 %, was reported only in *Rh*. *microplus* from 4 countries (Pakistan, China, Mozambique, and Ecuador). *Anaplasma ovis* is a gram-negative bacterium that causes disease in goats, sheep, and humans (Lu et al., 2022b; Thinnabut et al., 2023; Lu et al., 2022d; Lu et al., 2023; Guo et al., 2019a). Symptoms of the illness include fever, hepatosplenomegaly, and lymph node (Lu et al., 2022d). *Candidatus* Anaplasma boleense, 2.51 %, was reported only in *Rh*. *microplus* from China. *Candidatus* Anaplasma boleense is an obligatory intracellular Gram-negative bacterium that infects mammals, such as cervids and cattle (Lu et al., 2022d).

##### 4.2.1.2 Acinetobacter

*Acinetobacter* species are non-fastidious, aerobic, non-motile, Gram-negative bacilli belonging to the family Moraxellaceae, order Pseudomonadales, which are ubiquitous and commonly found in water, soil, and dry environments. Certain strains of *Acinetobacter* can infect fish and people with weakened immune systems (Kozińska et al., 2014; Cao et al., 2018; Almasaudi, 2018). Furthermore, in this systematic review, both *A. lwoffii* and *A. johnsonii*, 0.42 %, were reported only in *Rh*. *microplus* from Mexico, which are responsible for causing diseases in fish (Kozińska et al., 2014; Cao et al., 2018).

##### 4.2.1.3 Bartonella

*Bartonella* species are pleomorphic, fastidious, facultative intracellular rod bacteria belonging to the family Bartonellaceae, order Rhizobiales, and associated with a variety of human and animal diseases (Mahlobo-Shwabede et al., 2021; Tsai et al., 2011; Ehlers et al., 2020). They can spread through direct contact, such as scratches or bites from infected animals, as well as by blood-sucking arthropod vectors (Paulauskas et al., 2022). In this study, *B. bacilliformis*, 0.42 %, was identified only in *Rh*. *microplus* from Peru. *Bartonella bacilliformis* is known to cause Carrion's disease in humans. Bartonellosis occurs in two stages. These two phages are called “Oroya fever” for their early life-threatening symptoms and “Verruga Peruana” for their chronic cutaneous manifestations. Oroya fever is characterized by acute fever and hemolytic anemia (Mahlobo-Shwabede et al., 2021; Ehlers et al., 2020; Vayssier-Taussat et al., 2016; Majerova et al., 2021). *Bartonella chomelii* and *B. schoenbuchensis*, 0.42 %, were reported only in *Rh*. *microplus* from the Philippines. *Bartonella chomelii* is known to cause infectious diseases in ruminants worldwide, and cattle are considered reservoir hosts (Boularias et al., 2020; Chochlakis et al., 2020; Tahmasebi Ashtiani et al., 2024; Goncalves-Oliveira et al., 2020). *Bartonella schoenbuchensis* is an emerging zoonotic pathogen that causes infectious diseases in humans and cattle (de Bruin et al., 2015). *Bartonella henselae*, *B. clarridgeiae*, *B. elizabethae*, *B. rochalimae*, and *B. rattimassiliensis*, 0.42 %, were reported only in *Rh*. *microplus* from China. *Bartonella henselae* is the etiologic agent of cat scratch fever (cat scratch disease) in humans and can infect ruminants. The clinical symptoms of cat scratch fever include a skin rash, fever, and swollen lymph nodes. Cats are natural reservoirs of this pathogen, and they typically transmit it to humans through scratches and saliva. In addition, cat fleas and ticks are responsible for spreading *B. henselae* between cats (Vayssier-Taussat et al., 2016; Majerova et al., 2021; Boularias et al., 2020; Chochlakis et al., 2020; Tahmasebi Ashtiani et al., 2024; Razgunaite et al., 2021; Iannino et al., 2018; Kosoy et al., 2010). *Bartonella clarridgeiae* is a causative agent of cat scratch disease-like illness in humans. It can cause endocarditis and hepatic disease in dogs, as well as blindness and neuritis in cats (Majerova et al., 2021; Tahmasebi Ashtiani et al., 2024; Cheslock and Embers, 2019). *Bartonella elizabethae* is considered the causative agent of endocarditis and neuroretinitis in humans and can sometimes infect dogs. Rodents and shrews are natural reservoirs of this zoonotic pathogen (Vayssier-Taussat et al., 2016; Majerova et al., 2021; Tahmasebi Ashtiani et al., 2024; Iannino et al., 2018; Pangjai et al., 2014; Tsai et al., 2010). *Bartonella rochalimae* is a zoonotic pathogen that can cause fever, myalgia, nausea, headache, mild cough, rash, anemia, and splenomegaly in humans. Wild animals, such as foxes, wolves, hedgehogs, raccoons, rodents, and dogs, are considered reservoirs (Majerova et al., 2021; Tahmasebi Ashtiani et al., 2024; Goncalves-Oliveira et al., 2020; Greco et al., 2021). *Bartonella rattimassiliensis* is a novel bacterium that infects rodents and shrews, and is thought to cause diseases in humans (Majerova et al., 2021; Tsai et al., 2010; Gundi et al., 2004).

##### 4.2.1.4 Borrelia

*Borrelia* species belonging to the phylum Spirochaetes, family Spirochaetaceae, and order Spirochaetales are Gram-negative bacteria that act as obligatory parasites. Furthermore, the majority of *Borrelia* detected in the studies included in this systematic review belong to the *Borrelia burgdorferi* sensu latu (s.l.) complex, which is usually transmitted by hard ticks and causes several zoonotic diseases (Khan et al., 2023; Rialch et al., 2022; Yu et al., 2016; Chu et al., 2008; Vorou et al., 2007; Veronesi et al., 2012). *Borrelia theileri*, 0.84 %, was reported only in *Rh*. *microplus* from Ecuador and Pakistan. *Borrelia theileri* is a tick-borne spirochete belonging to the relapsing fever group Borreliae (RFGB), which is known to cause bovine borreliosis in livestock such as goats, sheep, and cows (Khan et al., 2023; Figueiroa et al., 2023). *Borrelia garinii*, 0.84 %, and *B. valaisiana*, 0.42 %, were reported only in *Rh*. *microplus* from China. They are zoonotic pathogens belonging to the *Borrelia burgdorferi* sensu lato group, responsible for human Lyme disease. Infection usually results in neuroborreliosis (Chu et al., 2008; Ortega et al., 2024; Wang et al., 2024a). *Borrelia lusitaniae,* 2.13 %, was identified only in *Hy*. *lusitanicum* from Portugal. It is an emerging zoonotic pathogen belonging to the *Borrelia burgdorferi* sensu lato group. It is the causative agent of human Lyme borreliosis and can cause borreliosis in horses (Veronesi et al., 2012; Collares-Pereira et al., 2004; de Carvalho et al., 2008)*.*

##### 4.2.1.5 Coxiella

*Coxiella* species are obligate intracellular, Gram-negative, pleomorphic coccobacillus belonging to the family Coxiellaceae, which can infect humans and animals (Gurtler et al., 2014). In this review, 0.84 % of *C. burnetii* was identified in *Rh*. *microplus* from the Philippines and China, and 6.38 % in *Hy*. *lusitanicum* from Spain and Portugal. *Coxiella burnetii* is a strict intracellular Gram-negative bacterium that causes Query fever (Q fever), a zoonotic disease affecting both people and various animals, including sheep, cattle, camels, and goats (Galay et al., 2020; Ngnindji-Youdje et al., 2022; Kobayashi et al., 2021; Segura et al., 2024; Sumrandee et al., 2016; Yu et al., 2016; Gurtler et al., 2014; Ali et al., 2023b; Li et al., 2023; Jiao et al., 2021; Sánchez et al., 2022). The most common form of Q fever in humans is an acute febrile illness with non-specific symptoms. However, Q fever can also present as acute hepatitis and pneumonia or as a chronic illness that can be life-threatening in severe cases or even valvular endocarditis (Mahlobo-Shwabede et al., 2021; Kim et al., 2007). *Coxiella-*like endosymbionts (CLEs), 2.51 %, were reported only in *Rh*. *microplus* from 6 countries (Thailand, China, Zambia, India, Colombia, and Mexico). *Coxiella-*like endosymbionts are uncultured and relatively common in the microbiota of ticks worldwide, affecting the development, chemical defense, nutrition, and reproduction of hosts (Jiao et al., 2021; Thanchomnang et al., 2023). It was initially thought that CLEs were transformed into *C. burnetii* through genetic mutations and the acquisition of genes that define virulence from pathogens. However, recent research supported the alternative theory that CLEs evolved independently from pathogenic *Coxiella* (Kobayashi et al., 2021; Brenner et al., 2021; Sulyok et al., 2014; Duron et al., 2014).

##### 4.2.1.6 Dermatophilus

In this review, 0.42 % of *D. congolensis* was identified only in *Rh. microplus* from the Philippines. It is a facultative anaerobic actinomycete, a Gram-positive bacterium belonging to the family Dermatophilaceae that causes dermatophilosis (cutaneous streptothricosis) in a variety of domestic animals, including sheep, goats, cattle, camels, and equines, as well as in a wide range of wild species and occasionally people. Globally, the illness is most common in humid, tropical, and subtropical areas, where it is referred to by a variety of names, including “lumpy wool disease,” “cutaneous streptothricosis,” “rain scald,” “strawberry foot rot,” and “mud fever” (Branford et al., 2021).

##### 4.2.1.7 Ehrlichia

*Ehrlichia* species, which belong to the order Rickettsiales, are Gram-negative, obligate intracellular bacteria that can cause a wide range of infections in both humans and animals (Lu et al., 2022c; Yu et al., 2016). In this study, 5.02 % of *E*. *minasensis* was identified in *Rh*. *microplus* from 7 countries (Thailand, Philippines, China, Colombia, Kenya, Italy, and Panama), and 2.13 % was reported in *Hy*. *lusitanicum* from Morocco. *Ehrlichia minasensis* causes tick-borne bovine ehrlichiosis in cattle and is characterized by anemia, severe fever, lethargy, thrombocytopenia, and depression (Lu et al., 2022b; Xu et al., 2023; Lu et al., 2022c; Lu et al., 2022d). *Ehrlichia canis* 1.26 % was reported only in *Rh*. *microplus* from China and Colombia. It is a causative agent of canine monocytic ehrlichiosis (CME) in dogs globally. Moreover, it also causes ehrlichioses ranging from mild to severe or fatal in humans and other animals, including sheep, goats, foxes, and deer (Lu et al., 2022b; Intirach et al., 2024; Lu et al., 2022c; Yu et al., 2016). *Ehrlichia canis*-like and *E. chaffeensis*, 0.42 %, were reported only in *Rh*. *microplus* from China. *Ehrlichia canis*-like is a known pathogen that causes infectious diseases similar to *E. canis (*Lu et al., *2022c**;* Breitschwerdt et al., *2008**)*. *Ehrlichia chaffeensis* is a well-known pathogen that causes bovine ehrlichiosis in large ruminants and human monocytotropic ehrlichiosis (HME) (Lu et al., 2022b; Lu et al., 2022c; Galay et al., 2021; Lu et al., 2022d; Zhang et al., 2023; Lu et al., 2023; Parola et al., 2003; Yu et al., 2016; Bermudez et al., 2009). Morbilliform rash, fever, headache, rigor, myalgia, episcleritis, and malaise are symptoms of human monocytotropic ehrlichiosis (Tan et al., 2001). *Ehrlichia ruminantium* and *E. ruminantium*-like, 0.42 %, were reported only in *Rh*. *microplus* from Kenya and China, respectively. *Ehrlichia ruminantium* is the causative agent of heartwater or cowdriosis, which primarily affects domestic animals such as goats, sheep, and cattle, as well as wild ruminants, and features fever, anemia, and thrombocytopenia (Ngnindji-Youdje et al., 2022; Elhachimi et al., 2021; Thinnabut et al., 2023; Galay et al., 2021; Lu et al., 2022d). *Ehrlichia ruminantium*-like is a pathogen genetically and antigenically similar to *E. ruminantium* and is the causative agent of Palona Moutain Ehrlichia (PME) in humans, dogs, and goats. Moreover, deer are natural reservoirs of *Ehrlichia*, and ticks spread them to humans and other animals (Yu et al., 2016; Reeves et al., 2008; Yabsley et al., 2008).

##### 4.2.1.8 Francisella

In this systematic review, 2.13 % of *Francisella* spp. were reported only in *Hy*. *lusitanicum* from Italy. The *Francisella* spp., which belong to the family Francisellaceae, are strictly aerobic, non-motile, facultatively intracellular, Gram-negative coccobacillus, and they can contain species that infect a wide range of animals as well as humans (Sumrandee et al., 2016; Birkbeck et al., 2011; van Hoek, 2013; Petersen et al., 2009; Soto et al., 2018).

##### 4.2.1.9 Mycobacterium

The genus *Mycobacterium* is a rod-shaped, Gram-positive, non-motile, slender, non-spore-forming bacterium that belongs to the Mycobacteriaceae family and can have a bent or curved appearance. Pleomorphism allows cells to adopt a variety of shapes, from coccoid forms to long, slender rods (Cao et al., 2024; Morgado and Vicente, 2021). In this review, 0.42 % of *Mycobacterium abscessus* was reported only in *Rh*. *microplus* from Mexico. It is a notorious fast-growing mycobacterium (RGM) within the nontuberculous mycobacterium (NTM) species, emerging opportunistic pathogens that can cause widespread infections such as pulmonary lung disease, soft tissue, and skin in traumatized, immunocompromised, and postsurgical patients. It is ubiquitous in the environment and found in soils, freshwater rivers and lakes, animal drinking troughs, decaying vegetation, and oceans (Cao et al., 2024; Morgado and Vicente, 2021; To K et al., 2020).

##### 4.2.1.10 Rickettsia

*Rickettsia* species are obligate intracellular growth, Gram-negative coccobacilli of the family Rickettsiaceae. They can cause disease in a variety of animals and humans via the bite of an arthropod vector. Furthermore, the majority of *Rickettsia* detected in the studies included in this systematic review belong to the spotted fever group (SFG), which are recognized as the causative agents of important emerging tick-borne human infectious diseases (Zhang et al., 2023; Osorio et al., 2018; Vorou et al., 2007; Martinez Diaz et al., 2023; Wang et al., 2021a; Qin et al., 2019). *Rickettsia aeschlimanni*, 0.42 %, was identified in *Rh*. *microplus* from Pakistan, and 6.38 % in *Hy*. *lusitanicum* from Italy and Spain. *Rickettsia massiliae*, 1.26 % was identified in *Rh*. *microplus* from Pakistan and Zambia, and 2.17 % in *Hy*. *lusitanicum* from Spain. *Rickettsia aeschlimanni* and *R. massiliae* have the potential to spread zoonotic diseases because they belong to the spotted fever group (SFG) (Blanda et al., 2017; Mahlobo-Shwabede et al., 2021; Ghafar et al., 2020b; Milhano et al., 2010; Pereira et al., 2018; Zhang et al., 2023; Ghafar et al., 2020c; Qiu et al., 2016; Osorio et al., 2018; Parola et al., 2003; Vorou et al., 2007; Chisu et al., 2018b). Recently, they have been recognized as causative agents of emerging human rickettsioses or Mediterranean spotted fever-like (Phiri et al., 2023; Remesar et al., 2022; Castillo-Contreras et al., 2022; Qiu et al., 2016). In addition, *R*. *aeschlimanni* and *R. massiliae* present clinical symptoms in humans similar to Mediterranean spotted fever-like, including weakness, pain, high fever, chills, rash, and eschar (Phiri et al., 2023; Remesar et al., 2022; Zhang et al., 2023; Qiu et al., 2016). *Rickettsia slovaca*, 0.42 %, was identified in *Rh*. *microplus* from Pakistan and 6.38 % in *Hy*. *lusitanicum* from Spain. *Rickettsia raoultii*, 0.84 %, was reported only in *Rh*. *microplus* from Pakistan and China. *Rickettsia slovaca* and *R. raoultii* are emerging zoonotic spotted fever group (SFG) rickettsiae species. They both are etiologic agents of TIBOLA (tick-borne lymphadenopathy) or DEBONEL (Dermacentor-borne necrotic erythema and lymphadenopathy) in humans, characterized by scalp eschars and neck lymphadenopathy after a tick bite (Blanda et al., 2017; Milhano et al., 2010; Remesar et al., 2022; Pereira et al., 2018; Castillo-Contreras et al., 2022; Zhang et al., 2023; Lu et al., 2023; Qiu et al., 2016; Vorou et al., 2007; Wang et al., 2021a; Chisu et al., 2018b). *Rickettsia africae*, 1.26 %, was identified only in *Rh*. *microplus* from Cameroon, Kenya, and the Comoros. It belongs to the spotted fever group (SFG) rickettsiae, the etiologic agent of African tick-bite fever (ATBF) in humans, characterized by fever, headache, muscle pain, and skin rash. Nevertheless, on rare occasions, inflammatory cardiomyopathy and central nervous system neuropathy (Mahlobo-Shwabede et al., 2021; Phiri et al., 2023; Yssouf et al., 2014; Qiu et al., 2016; Parola et al., 2003; Jensenius et al., 2003). *Rickettsia felis*, 0.84 %, was identified only in *Rh*. *microplus* from Colombia and the United States. *Candidatus* Rickettsia senegalensis, 0.42 %, was reported only in *Rh*. *microplus* from the United States. *Rickettsia felis* is a spotted fever group of human pathogens found worldwide and is known to be an etiological agent of flea-borne spotted fever (Cleveland et al., 2019; Lu et al., 2023; Brown and Macaluso, 2016). However, the clinical manifestations of flea-borne rickettsiosis are indistinguishable fever. This fever is often accompanied by headache, malaise, and myalgia. Additionally, rashes can occur at various frequencies (Caravedo Martinez et al., 2021). *Candidatus* Rickettsia senegalensis is a species closely related to *R. felis*, which was recently proposed as a causative agent of human febrile illnesses similar to *R. felis* (Cleveland et al., 2019; Brown and Macaluso, 2016)*. Rickettsia japonica*, 0.42 %, was reported only in *Rh*. *microplus* from China*.* It belongs to the spotted fever group (SFG) rickettsiae is the etiologic agent of Japanese spotted fever (JSF), characterized by fever and rash. The geographical distribution of these agents continues to expand (Lu et al., 2017; Zhang et al., 2023; Lu et al., 2023; Wang et al., 2021a; Qin et al., 2019; Brown and Macaluso, 2016; Uchida, 1993). *Rickettsia monacensis*, 0.84 %, was reported only in *Rh*. *microplus* from Ecuador and Bangladesh. It is a member of the spotted fever group (SFG) rickettsia that has been involved in human infectious diseases (Milhano et al., 2010; Pereira et al., 2018; Chisu et al., 2018b). *Rickettsia* sp. Sw, 0.84 %, was reported only in *Rh*. *microplus* from China. It shares significant similarities with *R. aeschlimannii* and two other pathogenic spotted fever group rickettsiae species: *R. raoultii* and *R. massiliae*. It is a significant human pathogen, with several cases being recorded in certain countries (Wang et al., 2021a). *Rickettsia tamurae*, 0.85 %, was reported only in *Rh*. *microplus* from Thailand and Ecuador. It is a member of the spotted fever group (SFG) rickettsia, which has been implicated in human infectious diseases (Yuan et al., 2021; Imaoka et al., 2011). *Candidatus* Rickettsia xinyangensis, 0.42 %, was identified only in *Rh*. *microplus* from China. It is a novel uncultured Rickettsia species reported to cause diseases in humans and is characterized by mild fever, elevated hepatic enzyme levels, eschars on the body, and leukopenia (Xu et al., 2023). *Candidatus* Rickettsia. jingxinensis, 2.51 %, was reported only in *Rh*. *microplus* from China. It is a spotted fever group (SFG) rickettsiae that has been recovered from tick-bitten patients and has been suggested to be a potential human pathogen (Lu et al., 2022d; Jiao et al., 2021; Wang et al., 2021a; Guo et al., 2019b). *Rickettsia amblyommatis*, 0.84 %, was identified only in *Rh*. *microplus* from Pakistan and the United States. *Rickettsia amblyommatis* (formerly known as *Candidatus* Rickettsia amblyommii) is a Gram-negative bacterium belonging to the spotted fever group (SFG) Rickettsiae. Although it is not a recognized pathogen for animals or humans, there have been isolated cases that indicate it may cause human diseases similar to Mountain Spotted Fever (RMSF) (Cleveland et al., 2019; Novakova et al., 2015; Richardson et al., 2023; Yen et al., 2021). *Rickettsia bellii*, 0.42 %, was reported only in *Rh*. *microplus* from Brazil. It belongs to the ancestral group (AG) Rickettsiae and shares close relatives with the pathogens responsible for Rocky Mountain spotted fever and other tick-borne pathogens; nevertheless, its exact function as a human pathogen remains unclear. According to some research, *R. bellii* can artificially infect mammals with disease, and it is possible that arthropod bites could spread it to people (Szabó et al., 2019; Fournier et al., 2005; Ogata et al., 2006; Snellgrove et al., 2021; Seidi et al., 2024). *Rickettsia sibirica* subsp. *Mongolitimonae* and *R. helvetica* 2.13 % were reported only in *Hy*. *lusitanicum* from Spain and Portugal, respectively. *Rickettsia sibirica* subsp. *Mongolitimonae* belongs to the *Rickettsia sibirica* species and is responsible for lymphangitis-associated rickettsiosis in humans, and is characterized by fever, rash, eschar inoculation, headache, and painful lymph nodes (Milhano et al., 2010; Remesar et al., 2022; Pereira et al., 2018; Fournier et al., 2005; Li et al., 2020b). *Rickettsia helvetica* belongs to the spotted fever group (SFG) rickettsiae and has been identified as a causative agent of emerging human rickettsioses, characterized by rash, fever, headache, and eschar inoculation (Milhano et al., 2010; Qiu et al., 2016; Vorou et al., 2007; Fournier et al., 2005).

#### 4.2.2 Protozoa

##### 4.2.2.1 Babesia

*Babesia* species are intraerythrocytic parasites that cause tick-borne diseases in animals and humans worldwide. They are members of the phylum Apicomplexa, family Babesiidae, and order Piroplasmida (Li et al., 2020a; Puri et al., 2021). In this study, 2.93 % of *B. bigemina* was identified only in *Rh*. *microplus* from 6 countries (Ecuador, Pakistan, China, Guinea, Colombia, and Burkina Faso). *Babesia bovis*, 1.67 %, was reported in *Rh*. *microplus* from 4 countries (China, Pakistan, the Philippines, and Burkina Faso), and 2.13 % in *Hy*. *lusitanicum* from Morocco. *Babesia occultans*, 0.42 %, was identified in *Rh*. *microplus* from Pakistan and 2.13 % in *Hy*. *lusitanicum* from Morocco. *Babesia bigemina*, *B. bovis*, and *B*. *occultans* are the primary causative agents of bovine babesiosis in cattle, transmitted by ticks worldwide (Zhang et al., 2022b; Ngnindji-Youdje et al., 2022; Tsai et al., 2011; Elhachimi et al., 2021; Zhao et al., 2023; Rehman et al., 2019; Ghafar et al., 2020c; Yu et al., 2016; Ouedraogo et al., 2021; Zeb et al., 2019; Mahmoud et al., 2024). Recently, they have been recognized as being responsible for human babesiosis (Calvopina et al., 2023; Ord and Lobo, 2015). *Babesia microti*, 2.13 %, was reported only in *Hy*. *lusitanicum* from Portugal. It is the etiologic agent of human babesiosis worldwide, characterized by fever, sweat, malaise, fatigue, and headache (Li et al., 2020a; Fernández et al., 2022; Pereira et al., 2018; Yu et al., 2016; Calvopina et al., 2023; Ord and Lobo, 2015; Tanyel et al., 2015; Senanayake et al., 2012; Bullard et al., 2014; Kumar et al., 2021).

##### 4.2.2.2 Theileria

*Theileria* species are intraerythrocytic parasites that share several microbiologic and pathogenic features with *Babesia* and belong to the same phylum, Apicomplexa, of the family Theileridae, order Piroplasmida. Furthermore, they can cause infectious diseases in a wide range of domestic and wild animals globally and are transmitted by ticks (Sumrandee et al., 2015; Mans et al., 2015).

This systematic review identified 1.67 % of *T. orientalis* in *Rh*. *microplus* from Pakistan and China, and 2.13 % in *Hy*. *lusitanicum* from Morocco. It is a common causative pathogen of bovine and ovine theileriosis (Elhachimi et al., 2021; Pereira et al., 2018). *Theileria annulata*, 1.26 %, was reported in *Rh*. *microplus* from Pakistan and Burkina Faso, and 2.13 % in *Hy*. *lusitanicum* from Morocco. It is the etiological agent of tropical theileriosis in cattle (Elhachimi et al., 2021; Pereira et al., 2018; Rehman et al., 2019; Makenov et al., 2021; Zeb et al., 2019). *Theileria ovis*, *T*. *luwenshuni*, and *T*. *equi*, 0.42 %, were reported only in *Rh*. *microplus* from Pakistan, China, and Brazil, respectively. *Theileria ovis* is responsible for theileriosis in small ruminants worldwide (Rehman et al., 2019). *Theileria luwenshuni* is the causative agent of bovine theileriosis in goats and sheep (Zhao et al., 2023). *Theileria equi*, formerly known as *Babesia equi*, is the causative agent of equine piroplasmosis in horses (Pereira et al., 2018; Nogueira et al., 2017; Yu et al., 2016). *Theileria buffeli***,** 2.13 %, was reported only in *Hy*. *lusitanicum* from Morocco. *Theileria sinensis***,** 0.42 %, was identified only in *Rh*. *microplus* from China. *Theileria buffeli* is responsible for bovine theileriosis (Sumrandee et al., 2015; Elhachimi et al., 2021; Pereira et al., 2018). *Theileria sinensis* is the causative agent of benign bovine theileriosis in cattle and yaks (Wang et al., 2021b). *Theileria mutans* and *T. velifera*, 0.42 %, were reported only in *Rh*. *microplus* from Burkina Faso and Mozambique, respectively. *Theileria mutans* and *T. velifera* are the causative agents of diseases in cattle (Kartashov et al., 2021).

##### 4.2.2.3 Hepatozoon

*Hepatozoon* spp. are apicomplexan hemoparasites belonging to the phylum Apicomplexa of the family Hepatozoidae that can infect domestic and wild animals worldwide (Sumrandee et al., 2015; Ali et al., 2023b). This review reported only 0.84 % of *H. canis* in *Rh*. *microplus* from Bangladesh and China. *Hepatozoon canis* is the causative agent of hepatozoonosis in dogs and cats, transmitted by ingesting infected ticks worldwide (Sumrandee et al., 2015; Intirach et al., 2024; Ali et al., 2023b; Mohanta et al., 2024; Traversa et al., 2024; De Bonis et al., 2021).

#### 4.2.3 Viruses

##### 4.2.3.1 Orthonairovirus

*Orthonairoviruses* belong to the family Nairoviridae and are negative-sense single-stranded RNA viruses that usually infect particular ixodid or argasid ticks and subsequently transmit the viruses to small vertebrates, such as bats and birds, which act as reservoirs for tick infection (Wang et al., 2024a; Garrison et al., 2020). This systematic review identified 0.42 % of Crimean–Congo hemorrhagic fever virus in *Rh*. *microplus* from Pakistan and 8.51 % in *Hy*. *lusitanicum* from Spain. Crimean–Congo hemorrhagic fever virus (*Crimean-Congo hemorrhagic fever orthonairovirus*), belonging to the genus *Orthonairovirus*, is an emerging zoonotic tick-borne pathogen responsible for sporadic cases or outbreaks of hemorrhagic fever in humans worldwide. This zoonotic disease is contracted by the biting of infected ticks on people or by direct contact with the body fluids of sick people or animals, which is typified by headache, fever, vomiting, skin bleeding, diarrhea, and muscle aches (Shahid et al., 2021; Negredo et al., 2019; Paz Sanchez-Seco et al., 2022; Wang et al., 2024a; Shahhosseini et al., 2021; Kuhn et al., 2022; Moraga-Fernández et al., 2021; Bente et al., 2013). Dugbe orthonairovirus, 0.42 %, was reported only in *Rh*. *microplus* from Nigeria. It was first identified from ticks collected from cattle in Nigeria and is responsible for causing diseases in humans characterized by mild febrile (Daoduid et al., 2021). Nairobi sheep disease virus and Meihua Mountain virus, 0.42 %, were reported only in *Rh*. *microplus* from China. Nairobi sheep disease virus (*Orthonairovirus nairobiense)*, also known as Ganjam virus, belonging to the genus *Orthonairovirus*, is a zoonotic tick-borne pathogen that causes fever, headache, nausea, and vomiting in people and is associated with high fever, abortion, diarrhea, and high mortality in small ruminants, such as goats and sheep worldwide (Wang et al., 2024b; Yang et al., 2019; Liu et al., 2020; Wang et al., 2021c; Yang et al., 2023; Krasteva et al., 2020; Li et al., 2024). Furthermore, the Meihua Mountain virus is novel, is thought to share substantial similarities with the Hazara virus, and belongs to the Nairobi sheep disease (NSD) Genogroup (Zhang et al., 2022a).

##### 4.2.3.2 Norwavirus

The viruses of the genus *Norwavirus*, which belong to the family Nairoviridae, are novel. They were initially unclassified and included in the genus *Orthonairovirus*. Furthermore, this genus is known to infect ixodid ticks (Wang et al., 2024a; Garrison et al., 2020; Kuhn et al., 2022). This systematic review reported only 0.42 % of Beiji-nairovirus in *Rh*. *microplus* from China. Beiji-nairovirus, also known as *Norwavirus beijiense*, belongs to this genus and is a novel pathogen first discovered in ticks and has recently been associated with emerging tick-borne diseases in humans (Wang et al., 2024a; Wang et al., 2021c).

##### 4.2.3.3 Flavivirus

The viruses of the genus *Flavivirus*, recently known as the genus *Orthoflavivirus*, belong to the family Flaviviridae. They are small enveloped viruses with positive-sense RNA transmitted to mammals and bird hosts by arthropod vectors, such as ticks or mosquitoes. Certain viruses in this genus can spread among vertebrates, including bats or rodents, even without established arthropod vectors (Simmonds et al., 2017). This review reported only 2.51 % of Jingmen tick virus (JMTV) in *Rh. microplus* from China and Colombia. Jingmen tick virus is an unclassified genus of *flavivirus* that causes tick-borne febrile diseases in humans and is found in cattle and monkeys. Occasionally, JMTV can have a co-infection with the Crimean–Congo hemorrhagic fever virus (Lu et al., 2022b; Shi et al., 2021; Wang et al., 2024a; Li et al., 2023; Pang et al., 2022; Ergunay et al., 2023; Wu et al., 2023). Flavi-like viruses and Yanggou tick virus, 0.42 %, were reported only in *Rh*. *microplus* from Colombia and China, respectively. Bole tick virus 4, 1.26 %, was reported only in *Rh*. *microplus* from Colombia and China. Flavi-like viruses are known as unclassified viruses of the Flaviviridae family, an example of the Jingmen tick virus and other viruses that may be included in the proposed genus “*Koshovirus*,” which has been reported to infect plants, marine vertebrates, and invertebrate hosts (Wu et al., 2023). Furthermore, the Yanggou tick virus belongs to the Jingmenvirus (JMV) group and is a novel, unclassified, segmented, flavi-like virus (Wang et al., 2023; Kholodilov et al., 2021). Bole tick virus 4 also belongs to the flavi-like virus group. As some new human and zoonotic infections belong to this group, they may eventually be of interest to medical or veterinary (Molina-Hoyos et al., 2024).

##### 4.2.3.4 Hepacivirus

The viruses of the genus *Hepacivirus*, members of the family Flaviviridae, are known to infect humans, such as the hepatitis C virus (HCV), which is classified as a member of the species *Hepacivirus hominis*, the causative agent of chronic hepatitis. Several other hepaciviruses have recently been reported to cause disease in both humans and animals (Simmonds et al., 2017; Postler et al., 2023). This systematic review reported only 0.42 % of Bovine hepacivirus in *Rh*. *microplus* from China. Bovine hepacivirus, also known as *Hepacivirus bovis*, is a recently added member to the genus *Hepacivirus* and is thought to infect bovines (Baechlein et al., 2019).

##### 4.2.3.5 Parapoxvirus

The viruses of the genus *Parapoxvirus* belong to the family Poxviridae, subfamily Chordopoxvirinae. They are ovoid or brick-shaped and enveloped with double-stranded DNA viruses that are thought to be zoonotic or potentially zoonotic because they typically infect ungulates and cause illnesses that manifest in livestock, wildlife, and humans (McInnes et al., 2023). In this review, 0.42 % of the Orf virus was reported only in *Rh*. *microplus* from China. Orf virus (ORFV), also called *Parapoxvirus orf,* belongs to the *Parapoxvirus* genus and infects a variety of animals, including domestic and wild animals. It causes ecthyma contagiosum in humans. Small wild ruminants, cattle, sheep, goats, and deer are the primary victims of the disease, which can infect humans worldwide through contact with contaminated meat or infected animals (Zhang et al., 2022b; Wu et al., 2023; Andreani et al., 2019).

##### 4.2.3.6 Orthopoxvirus

The viruses of the genus *Orthopoxvirus* belong to the family Poxviridae, subfamily Chordopoxvirinae, and exclusively infect humans and rodents; however, certain viruses of this genus infect numerous hosts (McInnes et al., 2023). This systematic review reported only 0.42 % of the cowpox virus (CPXV) in *Rh. microplus* from China. The cowpox virus belongs to the genus *Orthopoxvirus* and shares profound similarities with other species, including vaccinia, variola, and monkeypox viruses. It is believed that the Cowpox virus is the etiologic pathogen that causes cowpox, primarily associated with lesions on the hands of dairy workers and the udders of dairy cows. Wild rodents are thought to be a reservoir host species in many parts of the world. In addition, direct contact with infected wild animals, pet rats, or cats is the main route of Cowpox virus transmission to people (Ferrier et al., 2021; Popova et al., 2017; Campe et al., 2009).

##### 4.2.3.7 Phlebovirus

Members of the genus *Phlebovirus*, belonging to the Phenuiviridae family, have segmented negative-sense RNA that can infect mammals, such as humans and livestock, and is spread via ticks, phlebotomine sandflies, and mosquitoes. Numerous species of this genus can cause large epizootic epidemics in humans and cattle (Klimentov et al., 2020; Sasaya et al., 2023). This study only reported 0.42 % of the Brown dog tick Phlebovirus 1 and YN tick-associated phlebovirus 1 in *Rh. microplus* from China. The Brown dog tick Phlebovirus 1 (BDTPV1) is categorized as an unclassified *Phlebovirus.* The primary vectors are ticks and mosquitoes, and the primary hosts are humans, camels, and ruminants (Wang et al., 2024a; Bratuleanu et al., 2022). Moreover, YN tick-associated phlebovirus 1 is mostly related to the *Uukuniemi phlebovirus* and is included in the *phlebovirus* genus. Although some viruses within the *Uukuniemi* group have the potential to cause diseases in humans, the group as a whole has not been thought to contain viruses that are significant for veterinary or public health. Individuals can develop acute self-limiting diseases distinguished by fever, facial hyperemia, muscle and joint pain, body rash, and headache (Shi et al., 2021; Palacios et al., 2013).

##### 4.2.3.8 Bandavirus

The viruses of the genus *Bandavirus*, belonging to the family Phenuiviridae, are spread by ticks to mammals, including humans, as well as birds (Sasaya et al., 2023). This study only reported 0.84 % of the Severe fever with thrombocytopenia syndrome virus (SFTSV) in *Rh. microplus* from China. Severe fever with thrombocytopenia syndrome virus, also known as *Bandavirus dabieense* or Dabie bandavirus, belongs to the genus *Bandavirus* and is a novel zoonotic tick-borne pathogen responsible for severe hemorrhagic disease in humans*.* The clinical signs and symptoms of the disease include high fever, leukocytopenia, thrombocytopenia, and gastrointestinal problems, along with joint pain, chills, and myalgia (Lu et al., 2022b; Wang et al., 2024a; Kuhn et al., 2022; Yang et al., 2023; Cui et al., 2024; Sun et al., 2024; Casel et al., 2021; Wang et al., 2015; Lopez et al., 2020).

##### 4.2.3.9 Uukuvirus

Members of the genus *Uukuvirus*, belonging to the Phenuiviridae family, are found in ticks and are occasionally associated with human diseases (Sasaya et al., 2023). This review reported only 0.84 % of the Dabieshan tick virus (DBTV) in *Rh*. *microplus* from China. Dabieshan tick virus, also known as *Uukuvirus dabieshanense* belongs to the genus *Uukuvirus*, was initially included in the genus *Orthohantavirus*, and was known as *Orthohantavirus dabieshanesnse*. It can cause renal syndrome and hemorrhagic fever in humans (Wang et al., 2021d; Han et al., 2024). The Lihan tick virus, 1.67 %, was identified only in *Rh*. *microplus* from Colombia and China. The Lihan tick virus, often referred to as *Uukuvirus lihanense*, belongs to the genus *Uukuvirus* and is a novel tick-borne pathogen with medical and veterinary public health implications because of its close association with numerous human and zoonotic infections (Molina-Hoyos et al., 2024; Lopez et al., 2020).

##### 4.2.3.10 Mivirus

The viruses of the genus *Mivirus* belong to the family Chuviridae and are non-segmented negative-sense RNA viruses that can infect a wide range of hosts, including vertebrates and invertebrates. In this review, 0.84 % of Wuhan tick virus 2 was identified only in *Rh*. *microplus* from Colombia and China. Wuhan tick virus 2, also known as *Mivirus wuhanense*, belongs to the genus *Mivirus*. It is thought to infect ticks and has the potential to cause zoonotic diseases (Gómez et al., 2020; Kuhn et al., 2023; Xu et al., 2021).

##### 4.2.3.11 Rhabdovirus

The viruses of the genus *Rhabdovirus* belong to the Rhabdoviridae family and are negative-sense (−) RNA viruses that are categorized as significant diseases of fisheries, livestock, agriculture, and public health (Walker et al., 2018; Dietzgen et al., 2017). Studies included in this systematic review reported 0.42 % of rhabdovirus-like viruses in *Rh*. *microplus* from China. They are thought to infect plants and arthropods (Dietzgen et al., 2017).

##### 4.2.3.12 Quaranjavirus

This systematic review identified 0.84 % of *Quaranjavirus* in *Rh. microplus* from China and Colombia. The viruses of the genus *Quaranjavirus*, which belong to the Orthomyxoviridae family, are a group of negative-sense segmented viruses with single-stranded RNA. Johnston Atoll quaranjavirus (JAV), Quaranfil quaranjavirus (QRFV), and unidentified quaranjavirus are the two species that comprise the genus *Quaranjavirus*. Viruses of this genus mainly infect humans, whereas ticks serve as the main vectors (Wang et al., 2024a; Presti et al., 2009).

##### 4.2.3.13 Timovirus

The genus *Timovirus*, belonging to the family Tymoviridae, is a monopartite single-stranded RNA virus. The studies included in this systematic review reported 0.42 % of Antioquia tymovirus-like 1 and Antioquia tymovirus-like 2. Viruses in *Rh*. *microplus* from Colombia. Viruses of this genus are thought to be responsible for plant diseases (Gómez et al., 2020; Yang et al., 2021; Orozco Orozco et al., 2021).

##### 4.2.3.14 Paslahepevirus

The viruses of the genus *Paslahepevirus*, belonging to the Hepeviridae family, are non-enveloped positive-sense RNA viruses that infect a wide range of mammals, including bats, rodents, sheep, deer, camels, rabbits, domestic and wild pigs, and birds (Purdy et al., 2017). The studies included in this systematic review reported 2.13 % of Hepatitis E Virus (HEV) in *Hy*. *lusitanicum* from Spain. This virus comprises two species, *Paslahepevirus balayani* and *Paslahepevirus alci.* Hepatitis E virus (HEV) is the leading cause of acute fulminant hepatitis and acute viral hepatitis. Symptoms of the disease include fever, icterus, diarrhea, nausea, anorexia, pruritus, and hepatomegaly. In immunocompromised patients, the infection can progress to chronic hepatitis, accompanied by extrahepatic manifestations, including hematological problems, neurological disorders, and renal failure. Hepatitis E Virus is transmitted by eating raw or undercooked infected meat or drinking contaminated water (Purdy et al., 2017; Ali et al., 2024; Lin et al., 2023; Yadav et al., 2021; Teshale et al., 2010; Zahmanova et al., 2023; Zahmanova et al., 2024).

## 5 Conclusion

The present systematic review summarizes the current distribution of *Rh*. *microplus* and/or *Hy*. *lusitanicum* and the pathogens they carry globally. It has demonstrated that *Rh*. *microplus* has a worldwide distribution and harbors a wide range of emerging and re-emerging tick-borne pathogens with public health and veterinary significance. *Hy*. *lusitanicum* is only documented in select countries in Europe and Africa and carries few tick-borne pathogens compared to *Rh*. *microplus.* To detect tick-borne pathogens in both *Rh*. *microplus* and *Hy*. *lusitanicum*, molecular techniques such as standard PCR assays, nested PCR assays, and conventional PCR assays have been most commonly used and have contributed to the discovery of novel tick-borne pathogens. This review will be helpful in managing and controlling ticks and tick-borne diseases of human and animal health significance by integrating the One Health perspective. We recommend continuing surveillance of these ticks and tick-borne pathogens on humans, domestic and wild animals, and the environment worldwide.

## CRediT authorship contribution statement

**Afito Luciano:** Conceptualization, Data curation, Formal analysis, Investigation, Methodology, Visualization, Writing – original draft, Writing – review & editing. **Binta J.J. Jallow:** Data curation, Formal analysis, Methodology, Writing – original draft, Writing – review & editing. **Mandie Liu:** Conceptualization, Validation, Visualization. **Yuting Ma:** Conceptualization, Validation, Visualization. **Regina Daniel Miambo:** Conceptualization, Visualization, Writing – review & editing. **Fanming Meng:** Writing – review & editing, Visualization, Validation, Supervision, Data curation, Funding acquisition, Investigation, Methodology.

## Funding

This work was supported by the 10.13039/501100001809National Natural Science Foundation of China (grant number 32370554, 32460248), 10.13039/501100004880Research and Innovation Team Project, Xinjiang Medical University (XYD2024C05), Science and Technology Innovation Team of Science & Technology Department of Xinjiang Uygur Autonomous Region (2024D14016)

## Declaration of Competing Interest

The authors declare no competing interests.

## Data Availability

We have shared supplementary files.

## Appendix A Supplementary data

Supplementary material Image 1
