# Supplementary material for: Distribution of Rhipicephalus microplus and Hyalomma lusitanicum, and the pathogens they are carrying: A systematic review
Source: Parasite Epidemiol Control. 2025 Jun 4;30:e00437. doi: 10.1016/j.parepi.2025.e00437 (PMC12205626; doi:10.1016/j.parepi.2025.e00437)
Supplement: Supplementary file 1 — Supplementary material [file mmc1.docx]

Supplementary Table 1: Summary of characteristics of the studies for the distribution of *Rh. microplus* and/or *Hy. lusitanicum.*

| Country | Host | *Rh. microplus* | *Hy. lusitanicum* | Total of ticks | Tick diagnostic methods | Ref. |
| --- | --- | --- | --- | --- | --- | --- |
| Pakistan | Buffalo, cattle, humans, goat | n=3584 | nd | 8,641 | Morphological and Molecular | (1) |
| India | Cattle, goats | n=1915 | nd | 3633 | Morphological | (2) |
| Uganda | Cattle | n=14 | nd | 4362 | Morphological and Molecular | (3) |
| China | Buffalo, cattle | n=489 | nd | 503 | Morphological and Molecular | (4) |
| Brazil | Horses | n=4 | nd | 2238 | Morphological | (5) |
| Benin | Bovines | n=6860 | nd | 14000 | Morphological and Molecular | (6) |
| Pakistan | Buffalo, cattle | n=221 | nd | 1200 | Morphological | (7) |
| Pakistan | Goats, sheep | n=3973 | nd | 6469 | Morphological and Molecular | (8) |
| Sri Lanka | Goats | n=88 | nd | 2628 | Morphological and Molecular | (9) |
| India | Buffalo, cattle, goats | n=2076 | nd | 8170 | Morphological | (10) |
| Mozambique | Donkeys | n=34 | nd | 500 | Morphological | (11) |
| Uganda | Cattle | n=94 | nd | 18637 | Morphological and Molecular | (12) |
| Uganda | Cattle | n=257 | nd | 13019 | Morphological and Molecular | (13) |
| Pakistan | Buffalo, cattle | n=66 | nd | 774 | Morphological and Molecular | (14) |
| Ghana | Cattle | n=46 | nd | 1355 | Morphological and Molecular | (15) |
| Laos | Cattle | n=1 | nd | 15073 | Morphological | (16) |
| South Africa | Cattle | n=105 | nd | 6176 | Morphological and Molecular | (17) |
| South Africa | Cattle, eland, gemsbok, rhebok, vegetation | n=9 | nd | 14482 | Morphological | (18) |
| Pakistan | Buffalo, cattle, dogs, donkeys, horses, goats, sheep | n=140 | nd | 408 | Morphological | (19) |
| China | Cattle, goats | n=606 | nd | 858 | Morphological and Molecular | (20) |
| Pakistan | Buffalo | n=86 | nd | 352 | Morphological and Molecular | (21) |
| Pakistan | Goats, sheep | n=542 | nd | 1499 | Morphological | (22) |
| Pakistan | Goats, sheep | n=514 | nd | 6257 | Morphological | (23) |
| Pakistan | Cattle | n=143 | nd | 1649 | Morphological | (24) |
| Kenya | Cattle | n=12 | nd | 35 | Morphological and Molecular | (25) |
| Pakistan | Cattle, goats, sheep | n=45 | nd | 1012 | Morphological | (26) |
| China | Cattle, goats | n=1111 | nd | 1461 | Morphological and Molecular | (27) |
| Benin | Cattle | n=404 | nd | 756 | Morphological and Molecular | (28) |
| Tanzania | Cattle | n=909 | nd | 1889 | Morphological and Molecular | (29) |
| Tanzania | Cattle | n=3533 | nd | 12940 | Morphological | (30) |
| Pakistan | Buffalo, cattle | n=493 | nd | 649 | Morphological and Molecular | (31) |
| India | Buffalo, cattle | n=2531 | nd | 10371 | Morphological and Molecular | (32) |
| Uganda | Cattle | n=687 | nd | 19007 | Morphological and Molecular | (33) |
| Bhutan | Cattle | n=2531 | nd | 3600 | Morphological | (34) |
| Mozambique | Dogs | n=1 | nd | 1898 | Morphological and Molecular | (35) |
| Burundi | Cattle | n=138 | nd | 483 | Morphological and Molecular | (36) |
| South Africa | Cattle | n=3978 | nd | 8361 | Morphological | (37) |
| Pakistan | Cattle, Sheep | n=724 | nd | 4080 | Morphological | (38) |
| Pakistan | Buffalo, cattle, goats, sheep | n=715 | nd | 3807 | Morphological and Molecular | (39) |
| Pakistan | Buffalo, cattle, goats, sheep | n=1474 | nd | 5557 | Morphological and Molecular | (40) |
| Indonesia | Cattle | n=1139 | nd | 1575 | Morphological | (41) |
| Colombia | Cattle | n=2449 | nd | 79277 | Morphological and Molecular | (42) |
| Zimbabwe | Cattle | n=2307 | nd | 21954 | Morphological | (43) |
| Cameroon | Cattle | n=708 | nd | 7091 | Morphological and Molecular | (44) |
| Cameroon | Cattle | n=1112 | nd | 7091 | Morphological and Molecular | (45) |
| Pakistan | Cattle | n=943 | nd | 2118 | Morphological and Molecular | (46) |
| Zimbabwe | Cattle, dogs, goats, sheep | n=1270 | nd | 7657 | Morphological and Molecular | (47) |
| Trinidad and Tobago | Cattle | n=1575 | nd | 3570 | Morphological | (48) |
| Ghana | Cattle | n=1181 | nd | 1489 | Morphological and Molecular | (49) |
| Argentina | Cattle | n=19 | nd | 292 | Morphological and Molecular | (50) |
| Lesotho | Cattle, dogs, donkeys, horses, goats, sheep | n=220 | nd | 3311 | Morphological and Molecular | (51) |
| Comoros | Cattle, goats | n=1317 | nd | 2118 | Morphological and Molecular | (52) |
| Pakistan | Dogs | n=23 | nd | 1150 | Morphological and Molecular | (53) |
| Brazil | Monkey | n=1 | nd | 130 | Morphological and Molecular | (54) |
| Spain | Cattle | nd | n=356 | 3624 | Morphological | (55) |
| Spain | Vegetation | nd | n=2444 | 185005 | Morphological | (56) |
| Malta | Cats, rabbits | nd | n=2 | nc | Morphological and Molecular | (57) |
| Spain | Rabbits | nd | n=88076 | 143283 | Morphological | (58) |
| Algeria | Cattle | nd | n=50 | 1214 | Morphological | (59) |
| Malta | Birds | nd | n=1 | 113 | Morphological and Molecular | (60) |
| Chad | Cattle | nd | n=398 | 1838 | Morphological | (61) |
| Spain | Humans | nd | n=307 | 8081 | Morphological | (62) |
| Pakistan | Cats | n=123 | nd | 531 | Morphological and Molecular | (63) |
| Pakistan | Dogs | n=69 | nd | 575 | Morphological | (64) |
| Pakistan | Cattle | n=137 | nd | 1119 | Morphological and Molecular | (65) |
| Pakistan | Cattle | n=131 | nd | 370 | Morphological | (66) |
| Argentina | Cattle, horses | n=613 | nd | 47996 | Morphological and Molecular | (67) |
| Colombia | Dogs | n=15 | nd | 193 | Morphological | (68) |
| India | Vegetation | n=3 | nd | 25169 | Morphological | (69) |
| China | Cattle, vegetation | n=388 | nd | 415 | Morphological | (70) |
| Panama | Cattle | n=10 | nd | 289 | Morphological and Molecular | (71) |
| Ecuador | Cattle | n=1251 | nd | 1251 | Morphological | (72) |
| Thailand | Cattle | n=15 | nd | 20 | Morphological and Molecular | (73) |
| China | Cattle | n=157 | nd | 403 | Morphological and Molecular | (74) |
| Brazil | Dogs | n=5 | nd | 1093 | Morphological | (75) |
| Pakistan | Cattle, sheep | n=3 | nd | 529 | Morphological and Molecular | (76) |
| Nigeria | Cattle | n=7557 | nd | 11826 | Morphological and Molecular | (77) |
| Brazil | Horses | n=45 | nd | 164 | Morphological | (78) |
| Costa Rica | Cattle, dogs, horses, vegetation | n=804 | nd | 4588 | Morphological | (79) |
| Brazil | Deer | n=5 | nd | 15 | Morphological | (80) |
| Mexico | Dogs | n=1 | nd | 33 | Morphological and Molecular | (81) |
| Pakistan | Buffalo, cattle, goats, sheep | n=89 | nd | 3807 | Morphological and Molecular | (82) |
| China | Cattle, goats | n=354 | nd | 1079 | Morphological and Molecular | (83) |
| Panama | Dogs | n=1 | nd | 2074 | Morphological | (84) |
| China | Vegetation | n=1334 | nd | 1615 | Morphological and Molecular | (85) |
| Peru | Cattle | n=9 | nd | 728 | Morphological | (86) |
| Philippines | Buffalo, cattle | n=206 | nd | 206 | Morphological | (87) |
| Philippines | Buffalo, cattle | n=195 | nd | 195 | Morphological | (88) |
| China | Vegetation | n=91 | nd | 778 | Morphological | (89) |
| Thailand | Cattle | n=1954 | nd | 2111 | Morphological and Molecular | (90) |
| China | Bovines, canine | n=180 | nd | 855 | Morphological and Molecular | (91) |
| China | Cattle | n=178 | nd | 178 | Morphological | (92) |
| Pakistan | Goats, sheep | n=15 | nd | 104 | Morphological and Molecular | (93) |
| Pakistan | Buffalo, cattle | n=17 | nd | 774 | Morphological and Molecular | (94) |
| China | Cattle, goats, sheep | n=456 | nd | 456 | Morphological and Molecular | (95) |
| Thailand | Boar, deer, snakes | n=30 | nd | 103 | Morphological | (96) |
| Philippines | Cattle | n=45 | nd | 45 | Morphological | (97) |
| China | Cattle | n=40 | nd | 447 | Morphological | (98) |
| China | Cattle, goats | n=291 | nd | 291 | Morphological and Molecular | (99) |
| China | Vegetation | n=170 | nd | 1815 | Morphological | (100) |
| Pakistan | Buffalo, cattle, cats, dogs, goats, poultry, sheep | n=35 | nd | 213 | Morphological | (101) |
| China | Cattle | n=516 | nd | 516 | Morphological and Molecular | (102) |
| Pakistan | Buffalo, camel, cattle, goat, sheep | n=416 | nd | 3866 | Morphological | (103) |
| Pakistan | Achai, buffalo, cattle, goats, Jersey, Sahiwal, sheep | n=254 | nd | 676 | Morphological | (104) |
| Pakistan | Camels, cattle, dogs, goats, sheep | n=73 | nd | 729 | Morphological | (105) |
| Malaysia | Cattle, goats, sheep | n=70 | nd | 270 | Morphological and Molecular | (106) |
| Zambia | Cattle, dogs, goats, vegetation | n=9 | nd | 175 | Morphological and Molecular | (107) |
| China | Cattle, goats, vegetation | n=1111 | nd | 2418 | Molecular | (108) |
| China | Cattle, goats | n=276 | nd | 276 | Morphological and Molecular | (109) |
| China | Cattle, goats | n=397 | nd | 434 | Morphological and Molecular | (110) |
| China | Dogs, goats | n=1 | nd | 113 | Morphological and Molecular | (111) |
| Lesotho | Cattle, donkeys, goats, horses, sheep, vegetation | n=440 | nd | 3311 | Morphological | (112) |
| Guinea | Cattle | n=16 | nd | 561 | Morphological and Molecular | (113) |
| Mozambique | Bovines | n=130 | nd | 646 | Morphological | (114) |
| Colombia | Cattle | nc | nd | nc | Morphological | (115) |
| India | Buffalo, cattle, dogs, goats | n=1490 | nd | 1778 | Morphological | (116) |
| China | Cattle, dogs | n=50 | nd | 83 | Morphological and Molecular | (117) |
| Thailand | Goats | n=93 | nd | 93 | Morphological and Molecular | (118) |
| Brazil | Dogs | n=4 | nd | 829 | Morphological | (119) |
| India | Bovines | n=79 | nd | 79 | Morphological and Molecular | (120) |
| Mexico | Wild pigs | n=1 | nd | 196 | Morphological | (121) |
| Madagascar | Cattle | n=176 | nd | 235 | Molecular | (122) |
| Thailand | Deer | n=31 | nd | 79 | Morphological and Molecular | (123) |
| Mexico | Deer | n=101 | nd | 355 | Morphological | (124) |
| China | Goats, sheep, vegetation | n=34 | nd | 947 | Morphological and Molecular | (125) |
| Colombia | Cattle, horses | n=417 | nd | 2809 | Morphological and Molecular | (126) |
| China | Cattle, vegetation | n=213 | nd | 849 | Morphological | (127) |
| China | Cattle | n=212 | nd | 581 | Morphological and Molecular | (128) |
| Bangladesh | Cattle | n=1432 | nd | 2287 | Morphological | (129) |
| Colombia | Cattle, vegetation | n=2088 | nd | 2088 | Morphological and Molecular | (130) |
| Bangladesh | Dogs | n=1 | nd | 53 | Morphological | (131) |
| Mexico | Deer | n=140 | nd | 290 | Morphological | (132) |
| Brazil | Horses | n=10 | nd | 5804 | Morphological | (133) |
| Cameroon | Cattle | n=552 | nd | 1483 | Morphological and Molecular | (134) |
| India | Buffalo, cattle | n=47 | nd | 602 | Morphological and Molecular | (135) |
| Brazil | Horses | n=6 | nd | 170 | Morphological | (136) |
| Colombia | Cattle, dogs, horses | n=273 | nd | 1247 | Morphological | (137) |
| Burkina Faso | Cattle | n=453 | nd | 3583 | Morphological and Molecular | (138) |
| Cameroon | Hedgehog, Nile monitor | n=32 | nd | 686 | Morphological and Molecular | (139) |
| China | Cattle, dogs, sheep, vegetation | n=850 | nd | 7316 | *Morphological and Molecular* | (140) |
| Thailand | Cattle, goats | n=106 | nd | 606 | Morphological | (141) |
| Ecuador | Cattle | n=60 | nd | 161 | Morphological and Molecular | (142) |
| Zambia | Cattle | n=1 | nd | 1465 | Morphological and Molecular | (143) |
| Belize | Cattle, vegetation | n= 488 | nd | 2506 | Morphological and Molecular | (144) |
| China | Vegetation | n=21 | nd | 182 | Molecular | (145) |
| Madagascar | Cattle | n= 732 | nd | 1822 | Morphological and Molecular | (146) |
| Pakistan | Buffalo, cattle, goats, sheep | n=121 | nd | 2183 | Morphological | (147) |
| China | Buffalo, cattle, hedgehogs | n=15 | nd | 341 | Morphological and Molecular | (148) |
| Bangladesh | Cattle | n=70 | nd | 154 | Morphological | (149) |
| Brazil | Coati | n=1 | nd | 1582 | Morphological | (150) |
| Pakistan | Buffalo, cattle | n=105 | nd | 338 | Morphological | (151) |
| Pakistan | Goats, sheep | n=55 | nd | 564 | Morphological | (152) |
| China | Buffalo, cattle | n=7387 | nd | 7387 | Morphological | (153) |
| Ethiopia | Cattle | n=27 | nd | 139 | Morphological | (154) |
| Pakistan | Buffalo, cattle, dogs, goats, sheep | n=262 | nd | 1873 | Morphological | (155) |
| Brazil | Wolf | n=1 | nd | 1206 | Morphological | (156) |
| Brazil | Vegetation | n=2 | nd | 4000 | Morphological | (157) |
| Kenya | Cattle | n=360 | nd | 3213 | Morphological and Molecular | (158) |
| Brazil | Anteater | n=149 | nd | 1775 | Morphological | (159) |
| Brazil | Tapirs | n=222 | nd | 5970 | Morphological | (160) |
| Guatemala | Cattle, horses | n=163 | nd | 291 | Morphological | (161) |
| South Africa | Gemsbok | n=8 | nd | 1873 | Morphological | (162) |
| El Salvador | Dogs | n=1 | nd | 1873 | Morphological and Molecular | (163) |
| Mexico | Dogs | n=3 | nd | 262 | Morphological | (164) |
| India | Cattle | n=589 | nd | 1471 | Morphological | (165) |
| Colombia | Cattle, dogs, horses, humans, pigs | n=50 | nd | 576 | Morphological | (166) |
| Colombia | Cattle, sheep | n=40 | nd | 62 | Morphological | (167) |
| China | Cattle, dogs, sheep | n=83 | nd | 2251 | Morphological | (168) |
| China | Cattle, vegetation | n=70 | nd | 389 | Morphological and Molecular | (169) |
| China | Cattle | n=166 | nd | 305 | Morphological | (170) |
| United States | Cats, dogs, vegetation | n=15 | nd | 1956 | Morphological and Molecular | (171) |
| China | Cattle | n=170 | nd | 296 | Morphological | (172) |
| China | Cattle, goats, vegetation | n=250 | nd | 1024 | Morphological and Molecular | (173) |
| China | Cattle | n=465 | nd | 465 | Morphological and Molecular | (174) |
| China | Cattle | n=40 | nd | 640 | Morphological and Molecular | (175) |
| Philippines | Cattle | n=60 | nd | 60 | Molecular | (176) |
| Comoros | Cattle, goats | n=296 | nd | 477 | Morphological | (177) |
| China | Cattle | n=400 | nd | 400 | Morphological and Molecular | (178) |
| Pakistan | Cattle | n=69 | nd | 123 | Morphological and Molecular | (179) |
| China | Boar, cattle | n=612 | nd | 988 | Morphological and Molecular | (180) |
| China | Cattle | n=20 | nd | 20 | Molecular | (181) |
| China | Cattle, goats, vegetation | n=131 | nd | 1113 | Morphological and Molecular | (182) |
| China | Cattle, goats | n=166 | nd | 179 | Morphological and Molecular | (183) |
| Italy | Humans | nd | n=11 | 42 | Morphological and Molecular | (184) |
| France | Humans | nd | n=1 | 248 | Morphological and Molecular | (185) |
| Spain | Deer | nd | n=204 | 210 | Morphological | (186) |
| Italy | Vegetation | nd | n=1 | 185 | Morphological | (187) |
| Italy | Ovine | nd | n=1 | 236 | Morphological | (188) |
| Portugal | Vegetation | nd | n=30 | 677 | Morphological | (189) |
| Morocco | Cattle | nd | n=101 | 2702 | Morphological | (190) |
| Portugal | Deer | nd | n=22 | 520 | Morphological | (191) |
| Spain | Deer, rabbit | nd | n=236 | 236 | Morphological | (192) |
| Italy | Hedgehog | nd | n=1 | 115 | Molecular | (193) |
| Algeria | Bovines | nd | n=53 | 1055 | Morphological | (194) |
| Spain | Boar, deer, goats, mouflon | nd | n=230 | 1246 | Morphological | (195) |
| Spain | Deer | nd | n=48 | 48 | Morphological | (196) |
| Spain | Cattle, vegetation | nd | n=680 | 2053 | Morphological | (197) |
| Spain | Boar, deer | nd | n=934 | 1012 | Morphological and Molecular | (198) |
| Spain | Hares, rabbits | nd | n=113 | 1129 | Morphological and Molecular | (199) |
| Spain | Cattle | nd | n=1079 | 1579 | Morphological | (200) |
| Spain | Boar, deer, mouflon | nd | n=85 | 89 | Morphological | (201) |
| Italy | Cattle, dogs, goats, sheep, vegetation | nd | n=161 | 678 | Morphological | (202) |
| Spain | Boars | nd | n=29 | 29 | Morphological and Molecular | (203) |
| Spain | Rabbits | nd | n=1285 | 19848 | Morphological | (204) |
| Portugal | Boar, cattle, deer, hare, horses, rabbit, sheep | nd | n=50 | 593 | Morphological | (205) |
| Spain | Boars | nd | n=1156 | 2256 | Morphological | (206) |
| Spain | Barbary sheep, Boar, deer, cattle, goats, mouflons, vegetation | nd | n=6820 | 12584 | Morphological | (207) |
| Bangladesh | Cattle, goats, vegetation | n=547 | nd | 1427 | Morphological | (208) |
| United States of America | Vegetation | n=59 | nd | 59 | Morphological | (209) |
| South Africa | Vegetation | n=14152 | nd | 14891 | Morphological | (210) |
| Algeria | Cattle | nd | n=39 | 253 | Morphological | (211) |
| Peru | Collared peccaries | n=67 | nd | 110 | Morphological | (212) |
| Colombia | Cattle | n=1358 | nd | 2365 | Morphological | (213) |
| Brazil | Buffalo | n=90 | nd | 200 | Morphological | (214) |
| China | Goats | n=6 | nd | 125 | Morphological | (215) |
| Panama | Cattle | n=14 | nd | 25 | Morphological | (216) |
| United States of America | Deer, wild pigs | n=78 | nd | 112 | Morphological | (217) |
| Spain | Boar, deer | nd | n=589 | 613 | Morphological and Molecular | (218) |
| Italy | Cattle | nd | n=1 | 794 | Morphological | (219) |
| Spain | Birds, boar, deer, dog, hedgehog, sheep, tortoise | nd | n=125 | 463 | Morphological and Molecular | (220) |
| Colombia | Cattle, dogs, donkeys | n=1482 | nd | 1745 | Morphological and Molecular | (221) |
| Spain | Deer, Vegetation | nd | n=555 | 956 | Morphological | (222) |
| Portugal | Deer, marten, mongoose, vegetation | nd | n=175 | 231 | Morphological | (223) |

* nd, No detected

Supplementary Table 2: Summary of characteristics of studies for the distribution of tick-borne pathogens in *Rh*. *microplus* and/or *Hy*. *lusitanicum*

| Country | Tick-borne pathogens detected in *Rh*. *microplus* | Tick-borne pathogens detected in *Hy. lusitanicum* | Tick-borne pathogens Diagnostic Methods | Ref. |
| --- | --- | --- | --- | --- |
| Pakistan | *Anaplasma* spp., *Coxiella* spp., *Hepatozoon* spp., *Ehrlichia* spp., *Rickettsia* spp. | nd | Standard PCR | (64) |
| Pakistan | *Anaplasma* spp. | nd | Standard PCR | (66) |
| China | *B. bovis*, *B*. *clarridgeiae*, *B. elizabethae*, *B. henselae*, *B*. *rattimassiliensis*, *B*. *rochalimae* | nd | Standard PCR | (70) |
| Panama | Spotted Fever Group Richettiae | nd | qPCR | (71) |
| Ecuador | *A. marginale*, *B. bigemina*, *B*. *theileri* | nd | Standard PCR | (72) |
| Thailand | *A. capra*, *A. centrale*, *A*. *marginale*, *A. platys*, *Coxiella-*like endosymbiont, *E*. *minasensis* | nd | Standard PCR | (73) |
| China | *A*. *marginale*, *Ca*. A. boleense, *Ca*. R. jingxinensis | nd | Hemi-nested PCR | (74) |
| Nigeria | dugbe orthonairovirus | nd | RT-qPCR | (77) |
| Costa Rica | *Rickettsia* spp. | nd | Standard PCR | (79) |
| Pakistan | *Anaplasma* spp., *A*. *centrale*, *A*. *marginale*, *B*. *bigemina*, *B*. *bovis*, *Ehrlichia* spp., *R. massiliae*, *R*. *raoultii*, *T*. *annulate*, *T*. *ovis*, *T*. *orientalis* | nd | Conventional PCR | (82) |
| China | *Anaplasma* spp., *A*. *marginale*, *A*. *platys*, *Rickettsia* spp., *Ca*. *A*. *boleense*, *Ehrlichia* spp., *E*. *canis* | nd | Nested PCR | (83) |
| China | *Babesia* spp. | nd | Nested PCR | (85) |
| Philippines | *C*. *burnetii* | nd | Conventional PCR, nested PCR | (87) |
| Philippines | *A*. *marginale*, *Ehrlichia* spp. | nd | Conventional PCR, nested PCR | (88) |
| Thailand | *Anaplasma* spp., *A. marginale*, *A*. *platys*, *Ehrlichia* spp., *E*. *minasensis* | nd | Standard PCR | (90) |
| China | Beiji nairovirus, Brown dog tick Phlebovirus 1, Cowpox virus, Jingmen tick virus, Quaranjavirus, rhabdo-like virus | nd | mNGS, standard PCR | (91) |
| China | Jingmen tick virus | nd | Conventional PCR, sanger sequencing | (92) |
| Pakistan | *A. centrale*, *A. ovis R*. *aeschlimanni*, *R*. *massiliae*, *R*. *slovaca* | nd | Conventional PCR | (93) |
| Pakistan | *A*. *marginale*, *Borrelia* spp. | nd | Conventional PCR, nested PCR | (94) |
| China | *A*. *capra*, *A*. *platys* | nd | Nested PCR | (95) |
| Thailand | *Theileria* spp. | nd | Standard PCR | (96) |
| Philippines | *B*. *bovis*, *B*. *chomelii*, *B*. *schoenbuchensis*, *D*. *congolensis*, *E*. *minasensis* | nd | Conventional PCR | (97) |
| China | *Borrelia* spp., *B*. *valaisiana* | nd | Nested PCR | (98) |
| China | *A*. *capra*, *A*. *marginale*, *A*. *platys*, *Rickettsia* spp., *Ehrlichia canis-*like | nd | Hemi-nested PCR | (99) |
| Pakistan | *Anaplasma* spp., *Theileria* spp. | nd | Standard PCR | (101) |
| China | *A. marginale*, *C*. *burnetiid*, *Ca*. R. jingxinensis, *Coxiella*-like endosymbionts | nd | mNGS, nested PCR | (102) |
| Pakistan | *R*. *amblyommatis* | nd | Nested PCR, qPCR | (103) |
| Pakistan | *Anaplasma* spp*., A*. *marginale* | nd | Conventional PCR | (104) |
| Pakistan | *B*. *theileri* | nd | Nested PCR, standard PCR | (105) |
| Zambia | *Coxiella-*like endosymbionts | nd | Nested PCR | (107) |
| China | *B. bigemina*, *T*. *luwenshuni*, *T*. *orientalis* | nd | Nested PCR | (108) |
| China | *A*. *capra*, *A*. *marginale*, *A*. *ovis*,  *Ca.* A. boleense*, Ca.* R. jingxinensis, *E*. *minasensi*, *E*. *minasensis*, *Ehrlichia* spp*.* | nd | Nested PCR | (109) |
| China | *A*. *marginale*, *A*. *ovis*, *Ca*. A. boleense, *Ca*. R. jingxinensis, *E*. *canis*, *E*. *chaffeensis*, *E*. *minasensis*, E*hrlichia* spp. | nd | Nested PCR, standard PCR | (110) |
| China | *Ca*. R. jingxinensis | nd | Standard PCR | (111) |
| Lesotho | *Anaplasma* spp. | nd | Standard PCR | (112) |
| Guinea | *B*. *bigemina*, *A*. *marginale*, *A*. *platys* | nd | qPCR | (113) |
| Mozambique | *A*. *ovis*, *T*. *velifera* | nd | Conventional PCR | (114) |
| Colombia | Antioquia tymovirus-like 1, Jingmen tick virus, Lihan tick virus, Wuhan tick virus 2 | nd | mNGS | (115) |
| India | *Anaplasma* spp., *A.  Central*, *Rickettsia* spp. | nd | Nested PCR, standard PCR | (116) |
| China | *A*. *marginale*, *A. platys*, *E*. *minasensis*, *Ehrlichia* spp., *T*. *sinensis*, *T*. *orientalis*, *Coxiella*-like bacteria | nd | mNGS, nested PCR | (117) |
| India | *Coxiella-*like endosymbionts | nd | Standard PCR | (120) |
| Madagascar | *Borrelia* spp., *Rickettsia* spp. | nd | Conventional PCR, qPCR | (122) |
| Thailand | *Rickettsia* spp., *Rickettsia tamurae* | nd | Standard PCR | (123) |
| Colombia | *A*. *marginale*, *E*. *minasensis*, *Rickettsia* spp. | nd | Conventional PCR, qPCR | (126) |
| China | *B*. *garinii*, *E*. *ruminantium-*like, *Ehrlichia* spp., *R*. *raoultii, Rickettsia* spp., *Theileria* spp. | nd | Nested PCR | (127) |
| China | *Ca*. R. longicornii, *Rickettsia* spp. | nd | Nested PCR | (128) |
| Colombia | *A*. *marginale*, *B*. *bigemina*, *Coxiella-*like endosymbiont, *R*. *felis* | nd | Nested PCR, RT-qPCR | (130) |
| Bangladesh | *H*. *canis* | nd | Standard PCR | (131) |
| Mexico | *A*. *aestuarii*, *A*. *lwoffii*, *A*. *johnsonii*, *Coxiella-like endosymbionts*, *Mycobacterium abscessus* | nd | mNGS, qPCR | (132) |
| Cameroon | *A*. *centrale*, *A*. *marginale*, *A*. *platys*, *Bartonela* spp., *Borrelia* spp., *Ehrlichia* spp., *R*. *africae*, *Rickettsia* spp. | nd | qRT-PCR, standard PCR | (134) |
| Brazil | *T*. *equi* | nd | Standard PCR | (136) |
| Colombia | *A*. *marginale*, *A*. *phagocitophylum*, *E*. *canis* | nd | Conventional PCR | (137) |
| Burkina Faso | *B*. *bigemina*, *B*. *bovis*, *T*. *annulata*, *T*. *mutans* | nd | Standard PCR, sanger sequencing | (138) |
| China | Jingmen tick virus | nd | qRT-PCR | (140) |
| Thailand | *Ehrlichia* spp. | nd | Standard PCR | (141) |
| Ecuador | *A*. *ovis*, *A*. *centrale*, *A*. *marginale*, *A*. *phagocytophilum*, *R*. *monacensis*, *R*. *tamurae* | nd | Standard PCR | (142) |
| Zambia | *R*. *massiliae* | nd | RT-PCR | (143) |
| China | *B*. *garinii* | nd | Nested PCR | (145) |
| Madagascar | *A*. *marginale* | nd | RT-PCR | (146) |
| Pakistan | Crimean-Congo hemorrhagic fever virus | nd | ELISA, nested PCR | (147) |
| China | *Ca*. R. jingxinensis | nd | Nested PCR | (148) |
| Bangladesh | *R*. *monacensis* | nd | RT-PCR | (149) |
| China | Jingmen tick virus, YN tick-associated phlebovirus 1 | nd | mNGS, nested RT-PCR | (153) |
| Pakistan | *Anaplasma* Spp. | nd | Standard PCR | (155) |
| Kenya | *Coxiella* spp., *E*. *minasensis*, *E*. *ruminantium*, *R*. *africae* | nd | Standard PCR | (158) |
| Brazil | *R*. *bellii* | nd | Standard PCR | (159) |
| Colombia | Bole Tick Virus 4, Flavi-like viruses, Quaranjavirus, Uukuvirus | nd | mNGS, nested PCR | (166) |
| Colombia | Antioquia tymovirus-like 2, Lihan tick virus, Bole tick virus 4 | nd | mNGS | (167) |
| China | Severe fever with thrombocytopenia syndrome virus | nd | RT-qPCR | (168) |
| China | Dabieshan tick virus | nd | Nested PCR, RT-qPCR | (169) |
| China | *Ca*. R. jingxinensis, *Rickettsia* spp. Sw | nd | Standard PCR | (170) |
| China | *Coxiella* spp., *Rickettsia* spp. | nd | mNGS | (172) |
| China | Dabieshan tick virus, Jingmen tick virus, Lihan tick virus, Nairobi sheep disease virus, Severe fever with thrombocytopenia virus, Wuhan tick virus 2 | nd | RT-PCR, Sanger sequencing | (173) |
| China | *A*. *bovis*, *A*. *marginale*, *A*. *platys*, *Ca*. A.boleense, *E*. *minasensis*, *Ehrlichia* spp., *Ca*. R. xinyangensis | nd | Standard PCR | (174) |
| China | Bole tick virus 4, Yanggou tick virus | nd | Nested PCR, Sanger sequencing | (175) |
| Philippines | *A*. *marginale*, *A*. *centrale* | nd | Nested PCR | (176) |
| Comoros | *R*. *africae* | nd | RT-qPCR | (177) |
| China | Bovine hepacivirus | nd | Standard PCR | (178) |
| Pakistan | *B. occultans*, *T*. *annulate* | nd | Standard PCR | (179) |
| China | Meihua Mountain virus | nd | mNGS, qPCR | (180) |
| China | *A*. *phagocytophilum*, *E*. *minasensis*, Orf virus | nd | mNGS | (181) |
| China | *A*. *marginale*, *Ca*. R. jingxinensis | nd | Nested PCR | (182) |
| China | *A. marginale*, *A*. *ovis*, *A*. *platys*, *B*. *bigemina*, *Ca*. A. boleense, *Ehrlichia* sp.*, E*. *minasensis*, *R*. *japonica*, *T*. *orientalis* | nd | RT-qPCR, standard PCR | (183) |
| Italy | nd | *R*. *aeschlimannii* | Standard PCR | (184) |
| Spain | nd | Crimean–Congo hemorrhagic fever virus | Nested PCR, RT-qPCR | (186) |
| Italy | nd | *R*. *aeschlimannii* | Standard PCR | (187) |
| Italy | nd | *Francisella* spp. | Standard PCR | (188) |
| Portugal | nd | *B*. *lusitaniae* | Culture, Standard PCR | (189) |
| Morocco | nd | *A*. *bovis*, *A*. *capra*, *A*. *marginale*, *A*. *phagocytophilum*, *A*. *platys*, *Anaplasma* spp., *B*. *bovis*, *B*. *occultans*, *E*. *minasensis*, *T*. *annulata*, *T*. *buffeli*, *T*. *orientalis* | Conventional PCR | (190) |
| Portugal | nd | *B*. *microti* | Conventional PCR, nested PCR | (191) |
| Spain | nd | *C*. *burnetii* | RT-PCR | (192) |
| Italy | nd | *A. phagocytophilum*, *A*. *platys* | Standard PCR | (193) |
| Algeria | nd | *Bartonella* spp. | RT-PCR | (194) |
| Spain | nd | *Anaplasma* spp., *Ehrlichia* spp. | Conventional PCR, sanger sequencing | (196) |
| Spain | nd | *R. slovaca*, *Rickettsia* spp. | Nested PCR | (198) |
| Spain | nd | *R*. *aeschlimannii*, *R. massiliae*, *R*. *sibirica* subsp. *mongolitimonae*, *R*. *slovaca* | Nested PCR | (199) |
| Spain | nd | Crimean–Congo hemorrhagic fever virus | Nested PCR | (200) |
| Spain | nd | *A*. *phagocytophilum, Anaplasma* spp. *Babesia* spp., *Ehrlichia* spp. *Theileria* spp. | Standard PCR | (201) |
| Italy | nd | *Anaplasma* spp., *Babesia* spp., *Ehrlichia* spp., *Theileria* spp. | Standard PCR | (202) |
| Spain | nd | Hepatitis E Virus | RT-qPCR | (203) |
| Spain | nd | *C*. *burnetii* | Standard PCR | (204) |
| Portugal | nd | *R*. *helvetica* | Conventional PCR, nested PCR | (205) |
| Spain | nd | *R. slovaca*, *Rickettsia* spp. | Standard PCR | (206) |
| Spain | nd | Crimean–Congo hemorrhagic fever virus | Nested RT-PCR, RT-PCR | (207) |
| Peru | *B*. *bacilliformis* | nd | RT-qPCR | (212) |
| Colombia | Lihan tick virus | nd | Conventional PCR, qPCR | (213) |
| Brazil | *A*. *marginale* | nd | Conventional PCR, qPCR | (214) |
| China | *Ehrlichia spp*., *H*. *canis* | nd | RT-qPCR | (215) |
| Panama | *A*. *marginale*, *E*. *minasensis* | nd | Standard PCR | (216) |
| United States of America | *Ca*. *R*. *senegalensis*, *R. amblyommatis*, *R*. *felis* | nd | Nested PCR | (217) |
| Spain | nd | Crimean–Congo hemorrhagic fever Virus | Nested RT-PCR | (218) |
| Spain | nd | *Borrelia* spp., *Rickettsia* spp. | RT-qPCR | (220) |
| Portugal | nd | *A. platys*, *C*. *burnetii* | Conventional PCR | (223) |

* nd, No detected

Supplementary Table 3: Assessment of quality of the included studies.

| References | Q1 | Q2 | Q3 | Q4 | Q5 | Q6 | Q7 | Q8 | Total Score | Percentage |
| --- | --- | --- | --- | --- | --- | --- | --- | --- | --- | --- |
| Ali (1) | 1 | 1 | 1 | 1 | 1 | 1 | 1 | 1 | 8 | 100 |
| Balasubramanian (2) | 1 | 1 | 1 | 1 | 1 | 1 | 1 | 1 | 8 | 100 |
| Balinandi (3) | 1 | 1 | 1 | 1 | 1 | 1 | 1 | 1 | 8 | 100 |
| Li (4) | 1 | 1 | 1 | 1 | 1 | 1 | 1 | 1 | 8 | 100 |
| Gama (5) | 1 | 1 | 1 | 1 | 1 | 1 | 1 | 0 | 7 | 87.5 |
| De Clercq (6) | 1 | 1 | 1 | 1 | 1 | 1 | 1 | 1 | 8 | 100 |
| Farooqi (7) | 1 | 1 | 1 | 1 | 1 | 1 | 1 | 1 | 8 | 100 |
| Ullah (8) | 1 | 1 | 1 | 1 | 1 | 1 | 1 | 1 | 8 | 100 |
| Diyes (9) | 1 | 1 | 1 | 1 | 1 | 1 | 1 | 1 | 8 | 100 |
| Elango (10) | 1 | 1 | 1 | 1 | 1 | 1 | 1 | 0 | 7 | 87.5 |
| Esculudis (11) | 1 | 1 | 1 | 1 | 1 | 1 | 1 | 1 | 8 | 100 |
| Etiang (12) | 1 | 1 | 1 | 1 | 1 | 1 | 1 | 1 | 8 | 100 |
| Etiang (13) | 1 | 1 | 1 | 1 | 1 | 1 | 1 | 1 | 8 | 100 |
| Ghafar (14) | 1 | 1 | 1 | 1 | 1 | 1 | 1 | 1 | 8 | 100 |
| Addo (15) | 1 | 1 | 1 | 1 | 1 | 1 | 1 | 1 | 8 | 100 |
| Vongphayloth (16) | 1 | 1 | 1 | 1 | 1 | 1 | 1 | 1 | 8 | 100 |
| Yawa (17) | 1 | 1 | 1 | 1 | 1 | 1 | 1 | 1 | 8 | 100 |
| Horak (18) | 1 | 1 | 1 | 1 | 1 | 1 | 1 | 1 | 8 | 100 |
| Hussain (19) | 1 | 1 | 1 | 1 | 1 | 1 | 1 | 1 | 8 | 100 |
| Intirach (20) | 1 | 1 | 1 | 1 | 1 | 1 | 1 | 1 | 8 | 100 |
| Iqbal (21) | 1 | 1 | 1 | 1 | 1 | 1 | 1 | 0 | 7 | 87.5 |
| Iqbal (22) | 1 | 1 | 1 | 1 | 1 | 1 | 1 | 1 | 8 | 100 |
| Iqbal (23) | 1 | 1 | 1 | 1 | 1 | 1 | 1 | 1 | 8 | 100 |
| Rafiq (24) | 1 | 1 | 1 | 1 | 1 | 1 | 1 | 1 | 8 | 100 |
| Kanduma (25) | 1 | 1 | 1 | 1 | 1 | 1 | 1 | 1 | 8 | 100 |
| Khan (26) | 1 | 1 | 1 | 1 | 1 | 1 | 1 | 1 | 8 | 100 |
| Li (27) | 1 | 1 | 1 | 1 | 1 | 1 | 1 | 1 | 8 | 100 |
| Madder (28) | 1 | 1 | 1 | 1 | 1 | 1 | 1 | 0 | 7 | 87.5 |
| Magesa (29) | 1 | 1 | 1 | 1 | 1 | 1 | 1 | 1 | 8 | 100 |
| Mamiro (30) | 1 | 1 | 1 | 1 | 1 | 1 | 1 | 1 | 8 | 100 |
| Nasreen (31) | 1 | 1 | 1 | 1 | 1 | 1 | 1 | 1 | 8 | 100 |
| Kandi (32) | 1 | 1 | 1 | 1 | 1 | 1 | 1 | 1 | 8 | 100 |
| Muhannguzi (33) | 1 | 1 | 1 | 1 | 1 | 1 | 1 | 1 | 8 | 100 |
| Namgyal (34) | 1 | 1 | 1 | 1 | 1 | 1 | 1 | 1 | 8 | 100 |
| Neves (35) | 1 | 1 | 1 | 1 | 1 | 1 | 1 | 0 | 7 | 87.5 |
| Nyabongo (36) | 1 | 1 | 1 | 1 | 1 | 1 | 1 | 1 | 8 | 100 |
| Nyangiwe (37) | 1 | 1 | 1 | 1 | 1 | 1 | 1 | 1 | 8 | 100 |
| Kebzai (38) | 1 | 1 | 1 | 1 | 1 | 1 | 1 | 1 | 8 | 100 |
| Rehman (39) | 1 | 1 | 1 | 1 | 1 | 1 | 1 | 1 | 8 | 100 |
| Rooman (40) | 1 | 1 | 1 | 1 | 1 | 1 | 1 | 1 | 8 | 100 |
| Sahara (41) | 1 | 1 | 1 | 1 | 1 | 1 | 1 | 0 | 7 | 87.5 |
| Segura (42) | 1 | 1 | 1 | 1 | 1 | 1 | 1 | 1 | 8 | 100 |
| Sungirai (43) | 1 | 1 | 1 | 1 | 1 | 1 | 1 | 1 | 8 | 100 |
| Silatsa (44) | 1 | 1 | 1 | 1 | 1 | 1 | 1 | 1 | 8 | 100 |
| Silatsa (45) | 1 | 1 | 1 | 1 | 1 | 1 | 1 | 1 | 8 | 100 |
| Sultan (46) | 1 | 1 | 1 | 1 | 1 | 1 | 1 | 1 | 8 | 100 |
| Sungirai (47) | 1 | 1 | 1 | 1 | 1 | 1 | 1 | 1 | 8 | 100 |
| Charles (48) | 1 | 1 | 1 | 1 | 1 | 1 | 1 | 0 | 7 | 87.5 |
| Tawiah-Mensah (49) | 1 | 1 | 1 | 1 | 1 | 1 | 1 | 1 | 8 | 100 |
| Lamattina (50) | 1 | 1 | 1 | 1 | 1 | 1 | 1 | 1 | 8 | 100 |
| Mahlobo-Shwabede (51) | 1 | 1 | 1 | 1 | 1 | 1 | 1 | 1 | 8 | 100 |
| Yssouf (52) | 1 | 1 | 1 | 1 | 1 | 1 | 1 | 1 | 8 | 100 |
| Zeb (53) | 1 | 1 | 1 | 1 | 1 | 1 | 1 | 1 | 8 | 100 |
| Zimmermann (54) | 1 | 1 | 1 | 1 | 1 | 1 | 1 | 1 | 8 | 100 |
| Castella (55) | 1 | 1 | 1 | 1 | 1 | 1 | 1 | 0 | 7 | 87.5 |
| Barandika (56) | 1 | 1 | 1 | 1 | 1 | 1 | 1 | 1 | 8 | 100 |
| Hornok (57) | 1 | 1 | 0 | 1 | 1 | 1 | 1 | 0 | 6 | 75 |
| Gonzalez (58) | 1 | 1 | 1 | 1 | 1 | 1 | 1 | 1 | 8 | 100 |
| Lotfi (59) | 1 | 1 | 1 | 1 | 1 | 1 | 1 | 1 | 8 | 100 |
| Hornok (60) | 1 | 1 | 1 | 1 | 1 | 1 | 1 | 1 | 8 | 100 |
| Zachee (61) | 1 | 1 | 1 | 1 | 1 | 1 | 1 | 1 | 8 | 100 |
| Vieira (62) | 1 | 1 | 1 | 1 | 1 | 1 | 1 | 1 | 8 | 100 |
| Ali (63) | 1 | 1 | 1 | 1 | 1 | 1 | 1 | 1 | 8 | 100 |
| Ali (64) | 1 | 1 | 1 | 1 | 1 | 1 | 1 | 1 | 8 | 100 |
| Ali (65) | 1 | 1 | 1 | 1 | 1 | 1 | 1 | 1 | 8 | 100 |
| Ali (66) | 1 | 1 | 1 | 1 | 1 | 1 | 1 | 1 | 8 | 100 |
| Copa (67) | 1 | 1 | 1 | 1 | 1 | 1 | 1 | 1 | 8 | 100 |
| Arroyave (68) | 1 | 1 | 1 | 1 | 1 | 1 | 1 | 1 | 8 | 100 |
| Balasubramanian (69) | 1 | 1 | 1 | 1 | 1 | 1 | 1 | 1 | 8 | 100 |
| Tsai (70) | 1 | 1 | 1 | 1 | 1 | 1 | 1 | 1 | 8 | 100 |
| Bermudez (71) | 1 | 1 | 1 | 1 | 1 | 1 | 1 | 1 | 8 | 100 |
| Gioia (72) | 1 | 1 | 1 | 1 | 1 | 1 | 1 | 1 | 8 | 100 |
| Thanchomnang (73) | 1 | 1 | 1 | 1 | 1 | 1 | 1 | 1 | 8 | 100 |
| Lu (74) | 1 | 1 | 1 | 1 | 1 | 1 | 1 | 1 | 8 | 100 |
| Costa (75) | 1 | 1 | 1 | 1 | 1 | 1 | 1 | 1 | 8 | 100 |
| Kasi (76) | 1 | 1 | 1 | 1 | 1 | 1 | 1 | 1 | 8 | 100 |
| Daodu (77) | 1 | 1 | 1 | 1 | 1 | 1 | 1 | 1 | 8 | 100 |
| Muraro (78) | 1 | 1 | 1 | 1 | 1 | 1 | 1 | 1 | 8 | 100 |
| Troyo (79) | 1 | 1 | 1 | 1 | 1 | 1 | 1 | 1 | 8 | 100 |
| Da Silveira (80) | 1 | 1 | 0 | 1 | 1 | 1 | 1 | 0 | 6 | 75 |
| Dzul-Rosado (81) | 1 | 1 | 1 | 1 | 1 | 1 | 1 | 1 | 8 | 100 |
| Rehman (82) | 1 | 1 | 1 | 1 | 1 | 1 | 1 | 1 | 8 | 100 |
| Lu (83) | 1 | 1 | 1 | 1 | 1 | 1 | 1 | 1 | 8 | 100 |
| Ferrell (84) | 1 | 1 | 1 | 1 | 1 | 1 | 1 | 1 | 8 | 100 |
| Chiang (85) | 1 | 1 | 1 | 1 | 1 | 1 | 1 | 1 | 8 | 100 |
| Flores-Mendoza (86) | 1 | 1 | 1 | 1 | 1 | 1 | 1 | 0 | 7 | 87.5 |
| Galay (87) | 1 | 1 | 1 | 1 | 1 | 1 | 1 | 1 | 8 | 100 |
| Galay (88) | 1 | 1 | 1 | 1 | 1 | 1 | 1 | 1 | 8 | 100 |
| Niu (89) | 1 | 1 | 1 | 1 | 1 | 1 | 1 | 1 | 8 | 100 |
| Thinnabut (90) | 1 | 1 | 1 | 1 | 1 | 1 | 1 | 1 | 8 | 100 |
| Wang (91) | 1 | 1 | 1 | 1 | 1 | 1 | 1 | 1 | 8 | 100 |
| Li (92) | 1 | 1 | 1 | 1 | 1 | 1 | 1 | 1 | 8 | 100 |
| Ghafar (93) | 1 | 1 | 1 | 1 | 1 | 1 | 1 | 1 | 8 | 100 |
| Ghafar (94) | 1 | 1 | 1 | 1 | 1 | 1 | 1 | 1 | 8 | 100 |
| Guo (95) | 1 | 1 | 1 | 1 | 1 | 1 | 1 | 1 | 8 | 100 |
| Sumrandee (96) | 1 | 1 | 1 | 1 | 1 | 1 | 1 | 1 | 8 | 100 |
| Hernandez (97) | 1 | 1 | 1 | 1 | 1 | 1 | 1 | 1 | 8 | 100 |
| Hou (98) | 1 | 1 | 1 | 1 | 1 | 1 | 1 | 1 | 8 | 100 |
| Lu (99) | 1 | 1 | 1 | 1 | 1 | 1 | 1 | 1 | 8 | 100 |
| Wang (100) | 1 | 1 | 1 | 1 | 1 | 1 | 1 | 1 | 8 | 100 |
| Jamil (101) | 1 | 1 | 1 | 1 | 1 | 1 | 1 | 1 | 8 | 100 |
| Jiao (102) | 1 | 1 | 1 | 1 | 1 | 1 | 1 | 1 | 8 | 100 |
| Karim (103) | 1 | 1 | 1 | 1 | 1 | 1 | 1 | 0 | 7 | 87.5 |
| Khan (104) | 1 | 1 | 1 | 1 | 1 | 1 | 1 | 1 | 8 | 100 |
| Khan (105) | 1 | 1 | 1 | 1 | 1 | 1 | 1 | 1 | 8 | 100 |
| Kho (106) | 1 | 1 | 1 | 1 | 1 | 1 | 1 | 1 | 8 | 100 |
| Kobayashi (107) | 1 | 1 | 1 | 1 | 1 | 1 | 1 | 1 | 8 | 100 |
| Li (108) | 1 | 1 | 1 | 1 | 1 | 1 | 1 | 1 | 8 | 100 |
| Lu (109) | 1 | 1 | 1 | 1 | 1 | 1 | 1 | 1 | 8 | 100 |
| Lu (110) | 1 | 1 | 1 | 1 | 1 | 1 | 1 | 1 | 8 | 100 |
| Lu (111) | 1 | 1 | 1 | 1 | 1 | 1 | 1 | 1 | 8 | 100 |
| Mahlobo-Shawabede (112) | 1 | 1 | 1 | 1 | 1 | 1 | 1 | 1 | 8 | 100 |
| Makenov (113) | 1 | 1 | 1 | 1 | 1 | 1 | 1 | 1 | 8 | 100 |
| Matsimbe (114) | 1 | 1 | 1 | 1 | 1 | 1 | 1 | 1 | 8 | 100 |
| Gomez (115) | 1 | 1 | 0 | 1 | 1 | 1 | 1 | 1 | 7 | 87.5 |
| Misra (116) | 1 | 1 | 1 | 1 | 1 | 1 | 1 | 1 | 8 | 100 |
| Intirach (117) | 1 | 1 | 1 | 1 | 1 | 1 | 1 | 1 | 8 | 100 |
| Aung (118) | 1 | 1 | 1 | 1 | 1 | 1 | 1 | 1 | 8 | 100 |
| Dos Santos (119) | 1 | 1 | 1 | 1 | 1 | 1 | 1 | 1 | 8 | 100 |
| Rialch (120) | 1 | 1 | 1 | 1 | 1 | 1 | 1 | 1 | 8 | 100 |
| Lopez-Perez (121) | 1 | 1 | 1 | 1 | 1 | 1 | 1 | 1 | 8 | 100 |
| Ehlers (122) | 1 | 1 | 1 | 1 | 1 | 1 | 1 | 1 | 8 | 100 |
| Sumrandee (123) | 1 | 1 | 1 | 1 | 1 | 1 | 1 | 1 | 8 | 100 |
| Ojida-Chi (124) | 1 | 1 | 1 | 1 | 1 | 1 | 1 | 1 | 8 | 100 |
| Guo (125) | 1 | 1 | 1 | 1 | 1 | 1 | 1 | 1 | 8 | 100 |
| Martinez (126) | 1 | 1 | 1 | 1 | 1 | 1 | 1 | 1 | 8 | 100 |
| Yu (127) | 1 | 1 | 1 | 1 | 1 | 1 | 1 | 1 | 8 | 100 |
| Yuan (128) | 1 | 1 | 1 | 1 | 1 | 1 | 1 | 1 | 8 | 100 |
| Roy (129) | 1 | 1 | 1 | 1 | 1 | 1 | 1 | 1 | 8 | 100 |
| Segura (130) | 1 | 1 | 1 | 1 | 1 | 1 | 1 | 1 | 8 | 100 |
| Mohanta (131) | 1 | 1 | 1 | 1 | 1 | 1 | 1 | 1 | 8 | 100 |
| Molina-Garza (132) | 1 | 1 | 1 | 1 | 1 | 1 | 1 | 1 | 8 | 100 |
| Neves (133) | 1 | 1 | 1 | 1 | 1 | 1 | 1 | 1 | 8 | 100 |
| Ngnindji-Youdje (134) | 1 | 1 | 1 | 1 | 1 | 1 | 1 | 1 | 8 | 100 |
| Nimisha (135) | 1 | 1 | 1 | 1 | 1 | 1 | 1 | 1 | 8 | 100 |
| Nogueira (136) | 1 | 1 | 1 | 1 | 1 | 1 | 1 | 1 | 8 | 100 |
| Osorio (137) | 1 | 1 | 1 | 1 | 1 | 1 | 1 | 1 | 8 | 100 |
| Ouedraogo (138) | 1 | 1 | 1 | 1 | 1 | 1 | 1 | 1 | 8 | 100 |
| Paguem (139) | 1 | 1 | 1 | 1 | 1 | 1 | 1 | 1 | 8 | 100 |
| Pang (140) | 1 | 1 | 1 | 1 | 1 | 1 | 1 | 1 | 8 | 100 |
| Parola (141) | 1 | 1 | 1 | 1 | 1 | 1 | 1 | 0 | 7 | 87.5 |
| Pesquera (142) | 1 | 1 | 1 | 1 | 1 | 1 | 1 | 0 | 7 | 87.5 |
| Phiri (143) | 1 | 1 | 1 | 1 | 1 | 1 | 1 | 1 | 8 | 100 |
| Polsomboon (144) | 1 | 1 | 1 | 1 | 1 | 1 | 1 | 1 | 8 | 100 |
| Chu (145) | 1 | 1 | 1 | 1 | 1 | 1 | 1 | 1 | 8 | 100 |
| Pothamann (146) | 1 | 1 | 1 | 1 | 1 | 1 | 1 | 1 | 8 | 100 |
| Shahid (147) | 1 | 1 | 1 | 1 | 1 | 1 | 1 | 1 | 8 | 100 |
| Qi (148) | 1 | 1 | 1 | 1 | 1 | 1 | 1 | 1 | 8 | 100 |
| Qiu (149) | 1 | 1 | 1 | 1 | 1 | 1 | 1 | 1 | 8 | 100 |
| De Sousa (150) | 1 | 1 | 1 | 1 | 1 | 1 | 1 | 1 | 8 | 100 |
| Sajid (151) | 1 | 1 | 1 | 1 | 1 | 1 | 1 | 1 | 8 | 100 |
| Shehla (152) | 1 | 1 | 1 | 1 | 1 | 1 | 1 | 1 | 8 | 100 |
| Shi (153) | 1 | 1 | 1 | 1 | 1 | 1 | 1 | 1 | 8 | 100 |
| Solomon (154) | 1 | 1 | 1 | 1 | 1 | 1 | 1 | 1 | 8 | 100 |
| Alam (155) | 1 | 1 | 1 | 1 | 1 | 1 | 1 | 1 | 8 | 100 |
| Arrais (156) | 1 | 1 | 1 | 1 | 1 | 1 | 1 | 0 | 7 | 87.5 |
| Sousa (157) | 1 | 1 | 1 | 1 | 1 | 1 | 1 | 1 | 8 | 100 |
| Oundo (158) | 1 | 1 | 1 | 1 | 1 | 1 | 1 | 1 | 8 | 100 |
| Szabo (159) | 1 | 1 | 1 | 1 | 1 | 1 | 1 | 1 | 8 | 100 |
| Labruna (160) | 1 | 1 | 1 | 1 | 1 | 1 | 1 | 1 | 8 | 100 |
| Teglas (161) | 1 | 1 | 1 | 1 | 1 | 1 | 1 | 1 | 8 | 100 |
| Tonetti (162) | 1 | 1 | 1 | 1 | 1 | 1 | 1 | 0 | 7 | 87.5 |
| Romero (163) | 1 | 1 | 1 | 1 | 1 | 1 | 1 | 1 | 8 | 100 |
| Ojeda-Chi (164) | 1 | 1 | 1 | 1 | 1 | 1 | 1 | 1 | 8 | 100 |
| Velusamy (165) | 1 | 1 | 1 | 1 | 1 | 1 | 1 | 1 | 8 | 100 |
| Molina-Hoyos (166) | 1 | 1 | 1 | 1 | 1 | 1 | 1 | 1 | 8 | 100 |
| Orozco (167) | 1 | 1 | 1 | 1 | 1 | 1 | 1 | 1 | 8 | 100 |
| Wang (168) | 1 | 1 | 1 | 1 | 1 | 1 | 1 | 1 | 8 | 100 |
| Wang (169) | 1 | 1 | 1 | 1 | 1 | 1 | 1 | 1 | 8 | 100 |
| Wang (170) | 1 | 1 | 1 | 1 | 1 | 1 | 1 | 1 | 8 | 100 |
| Weaver (171) | 1 | 1 | 1 | 1 | 1 | 1 | 1 | 1 | 8 | 100 |
| Xiang (172) | 1 | 1 | 1 | 1 | 1 | 1 | 1 | 1 | 8 | 100 |
| Xu (173) | 1 | 1 | 1 | 1 | 1 | 1 | 1 | 1 | 8 | 100 |
| Xu (174) | 1 | 1 | 1 | 1 | 1 | 1 | 1 | 1 | 8 | 100 |
| Yang (175) | 1 | 1 | 1 | 1 | 1 | 1 | 1 | 1 | 8 | 100 |
| Ybanez (176) | 1 | 1 | 1 | 1 | 1 | 1 | 1 | 1 | 8 | 100 |
| Yssouf (177) | 1 | 1 | 1 | 1 | 1 | 1 | 1 | 1 | 8 | 100 |
| Yuan (178) | 1 | 1 | 1 | 1 | 1 | 1 | 1 | 1 | 8 | 100 |
| Zeb (179) | 1 | 1 | 1 | 1 | 1 | 1 | 1 | 1 | 8 | 100 |
| Zhang (180) | 1 | 1 | 1 | 1 | 1 | 1 | 1 | 1 | 8 | 100 |
| Zhang (181) | 1 | 1 | 0 | 1 | 1 | 1 | 1 | 1 | 7 | 87.5 |
| Zhang (182) | 1 | 1 | 1 | 1 | 1 | 1 | 1 | 1 | 8 | 100 |
| Zhao (183) | 1 | 1 | 1 | 1 | 1 | 1 | 1 | 1 | 8 | 100 |
| Blanda (184) | 1 | 1 | 1 | 1 | 1 | 1 | 1 | 1 | 8 | 100 |
| Aubry (185) | 1 | 1 | 1 | 1 | 1 | 1 | 1 | 1 | 8 | 100 |
| Cajimat (186) | 1 | 1 | 1 | 1 | 1 | 1 | 1 | 1 | 8 | 100 |
| Chisu (187) | 1 | 1 | 1 | 1 | 1 | 1 | 1 | 1 | 8 | 100 |
| Chisu (188) | 1 | 1 | 1 | 1 | 1 | 1 | 1 | 1 | 8 | 100 |
| Milhano (189) | 1 | 1 | 1 | 1 | 1 | 1 | 1 | 0 | 7 | 87.5 |
| Elhachimi (190) | 1 | 1 | 1 | 1 | 1 | 1 | 1 | 1 | 8 | 100 |
| Fernandez (191) | 1 | 1 | 1 | 1 | 1 | 1 | 1 | 1 | 8 | 100 |
| Gonzalez (192) | 1 | 1 | 1 | 1 | 1 | 1 | 1 | 1 | 8 | 100 |
| Chisu (193) | 1 | 1 | 1 | 1 | 1 | 1 | 1 | 1 | 8 | 100 |
| Boularias (194) | 1 | 1 | 1 | 1 | 1 | 1 | 1 | 0 | 7 | 87.5 |
| Marquez (195) | 1 | 1 | 1 | 1 | 1 | 1 | 1 | 1 | 8 | 100 |
| Diaz (196) | 1 | 1 | 1 | 1 | 1 | 1 | 1 | 1 | 8 | 100 |
| Palomar (197) | 1 | 1 | 1 | 1 | 1 | 1 | 1 | 0 | 7 | 87.5 |
| Remesar (198) | 1 | 1 | 1 | 1 | 1 | 1 | 1 | 1 | 8 | 100 |
| Remesar (199) | 1 | 1 | 1 | 1 | 1 | 1 | 1 | 1 | 8 | 100 |
| Negredo (200) | 1 | 1 | 1 | 1 | 1 | 1 | 1 | 1 | 8 | 100 |
| Diaz-Cao (201) | 1 | 1 | 1 | 1 | 1 | 1 | 1 | 1 | 8 | 100 |
| Torina (202) | 1 | 1 | 1 | 1 | 1 | 1 | 1 | 1 | 8 | 100 |
| Rivero (203) | 1 | 1 | 1 | 1 | 1 | 1 | 1 | 1 | 8 | 100 |
| Sanchez (204) | 1 | 1 | 1 | 1 | 1 | 1 | 1 | 1 | 8 | 100 |
| Pereira (205) | 1 | 1 | 1 | 1 | 1 | 1 | 1 | 1 | 8 | 100 |
| Castillo (206) | 1 | 1 | 1 | 1 | 1 | 1 | 1 | 1 | 8 | 100 |
| Paz Santchez-Seco (207) | 1 | 1 | 1 | 1 | 1 | 1 | 1 | 1 | 8 | 100 |
| Islam (208) | 1 | 1 | 1 | 1 | 1 | 1 | 1 | 0 | 7 | 87.5 |
| Osbrink (209) | 1 | 1 | 1 | 1 | 1 | 1 | 1 | 0 | 7 | 87.5 |
| Nyangiwe (210) | 1 | 1 | 1 | 1 | 1 | 1 | 1 | 1 | 8 | 100 |
| Mokhtaria (211) | 1 | 1 | 1 | 1 | 1 | 1 | 1 | 0 | 7 | 87.5 |
| Valle-Mendoza (212) | 1 | 1 | 1 | 1 | 1 | 1 | 1 | 0 | 7 | 87.5 |
| Lopez (213) | 1 | 1 | 1 | 1 | 1 | 1 | 1 | 1 | 8 | 100 |
| Da Silva (214) | 1 | 1 | 1 | 1 | 1 | 1 | 1 | 1 | 8 | 100 |
| Li (215) | 1 | 1 | 1 | 1 | 1 | 1 | 1 | 0 | 7 | 87.5 |
| Bermudez (216) | 1 | 1 | 1 | 1 | 1 | 1 | 1 | 1 | 8 | 100 |
| Cleveland (217) | 1 | 1 | 1 | 1 | 1 | 1 | 1 | 1 | 8 | 100 |
| Moraga-Fernandez (218) | 1 | 1 | 1 | 1 | 1 | 1 | 1 | 1 | 8 | 100 |
| Jimale (219) | 1 | 1 | 1 | 1 | 1 | 1 | 1 | 1 | 8 | 100 |
| Ortega (220) | 1 | 1 | 1 | 1 | 1 | 1 | 1 | 1 | 8 | 100 |
| Rivera-Paez (221) | 1 | 1 | 1 | 1 | 1 | 1 | 1 | 1 | 8 | 100 |
| Jouglin (222) | 1 | 1 | 1 | 1 | 1 | 1 | 1 | 0 | 7 | 87.5 |
| Santos-Silva (223) | 1 | 1 | 1 | 1 | 1 | 1 | 1 | 0 | 7 | 87.5 |

*Q1: Was the sample frame appropriate to address the target population?

Q2: Were study participants sampled in as appropriate way?

Q3: Was the sample size adequate?

Q4: Were the study subjects and the setting described in detail?

Q5: Was the data analysis conducted with sufficient coverage of the identified sample?

Q6: Was valid method used for the identification of the condition?

Q7: Was the condition measured in a standard, reliable way for all participants?

Q8: Was there appropriate statistical analysis?

Supplementary Table 4: Frequency of tick-borne pathogens in *Rh*. *microplus* and/or *Hy*. *lusitanicum*

| **Tick-borne Pathogens** | **N^o^. studies-*Rh*. *microplus*** | **Percentage** | **N^o^. studies-*Hy*. *lusitanicum*** | **Percentage** |
| --- | --- | --- | --- | --- |
| **Bacteria** | 171 | 71.55 | 32 | 68.09 |
| ***Anaplasma*** | 64 | 26.78 | 9 | 19.15 |
| *A*. *bovis* | 1 | 0.42 | 1 | 2.13 |
| *A*. *capra* | 4 | 1.67 | 1 | 2.13 |
| *A*. *centrale* | 7 | 2.93 | 0 | 0.00 |
| *A*. *marginale* | 27 | 11.30 | 1 | 2.13 |
| *A*. *ovis* | 6 | 2.51 | 0 | 0.00 |
| *A*. *phagocitophylum* | 3 | 1.26 | 3 | 6.38 |
| *A*. *platys* | 10 | 4.18 | 3 | 6.38 |
| *Ca*. A. boleense | 6 | 2.51 | 0 | 0.00 |
| ***Acinetobacter*** | 2 | 0.84 | 0 | 0.00 |
| *A*. *johnsonii* | 1 | 0.42 | 0 | 0.00 |
| *A*. *lwoffii* | 1 | 0.42 | 0 | 0.00 |
| ***Bartonella*** | 9 | 3.77 | 1 | 2.13 |
| *B*. *bacilliformis* | 1 | 0.42 | 0 | 0.00 |
| *B*. *chomelii* | 1 | 0.42 | 0 | 0.00 |
| *B*. *clarridgeiae* | 1 | 0.42 | 0 | 0.00 |
| *B*. *elizabethae* | 1 | 0.42 | 0 | 0.00 |
| *B*. *henselae* | 1 | 0.42 | 0 | 0.00 |
| *B*. *rattimassiliensis* | 1 | 0.42 | 0 | 0.00 |
| *B*. *rochalimae* | 1 | 0.42 | 0 | 0.00 |
| *B*. *schoenbuchensis* | 1 | 0.42 | 0 | 0.00 |
| *Bartonella* spp. | 1 | 0.42 | 1 | 2.13 |
| ***Borrelia*** | 9 | 3.77 | 2 | 4.26 |
| *B*. *garinii* | 2 | 0.84 | 0 | 0.00 |
| *B*. *lusitaniae* | 0 | 0.00 | 1 | 2.13 |
| *Borrelia* spp. | 4 | 1.67 | 1 | 2.13 |
| *B*. *theileri* | 2 | 0.84 | 0 | 0.00 |
| *B*. *valaisiana* | 1 | 0.42 | 0 | 0.00 |
| ***Coxiella*** | 11 | 4.60 | 3 | 6.38 |
| *C*. *burnetii* | 2 | 0.84 | 3 | 6.38 |
| *Coxiella-*like endosymbionts | 6 | 2.51 | 0 | 0.00 |
| *Coxiella* spp. | 3 | 1.26 | 0 | 0.00 |
| ***Dermatophilus*** | 1 | 0.42 | 0 | 0.00 |
| *D*. *congolensis* | 1 | 0.42 | 0 | 0.00 |
| ***Ehrlichia*** | 33 | 13.81 | 4 | 8.51 |
| *E*. *canis* | 3 | 1.26 | 0 | 0.00 |
| *E*. *canis-*like | 1 | 0.42 | 0 | 0.00 |
| *E*. *chaffeensis* | 1 | 0.42 | 0 | 0.00 |
| *E*. *minasensis* | 12 | 5.02 | 1 | 2.13 |
| *E*. *ruminantium* | 1 | 0.42 | 0 | 0.00 |
| *E*. *Ruminantium-*like | 1 | 0.42 | 0 | 0.00 |
| *Ehrlichia* spp. | 14 | 5.86 | 3 | 6.38 |
| ***Francisella*** | 0 | 0.00 | 1 | 2.13 |
| *Francisella* spp. | 0 | 0.00 | 1 | 2.13 |
| ***Mycobacterium*** | 1 | 0.42 | 0 | 0.00 |
| *M*. *abscessus* | 1 | 0.42 | 0 | 0.00 |
| ***Rickettsia*** | 41 | 17.15 | 12 | 25.53 |
| *R*. *aeschlimanni* | 1 | 0.42 | 3 | 6.38 |
| *R*. *africae* | 3 | 1.26 | 0 | 0.00 |
| *R*. *amblyommatis* | 2 | 0.84 | 0 | 0.00 |
| *R*. *bellii* | 1 | 0.42 | 0 | 0.00 |
| *R*. *felis* | 2 | 0.84 | 0 | 0.00 |
| *R*. *helvetica* | 0 | 0.00 | 1 | 2.13 |
| *R*. *japonica* | 1 | 0.42 | 0 | 0.00 |
| *R*. *massiliae* | 3 | 1.26 | 1 | 2.13 |
| *R*. *monacensis* | 2 | 0.84 | 0 | 0.00 |
| *R*. *raoultii* | 2 | 0.84 | 0 | 0.00 |
| *R*. *sibirica* subsp. *Mongolitimonae* | 0 | 0.00 | 1 | 2.13 |
| *R*. *slovaca* | 1 | 0.42 | 3 | 6.38 |
| *Rickettsia* spp. | 12 | 5.02 | 3 | 6.38 |
| *Rickettsia* sp. Sw | 1 | 0.42 | 0 | 0.00 |
| *R*. *tamurae* | 2 | 0.84 | 0 | 0.00 |
| *Ca*. R. jingxinensis | 6 | 2.51 | 0 | 0.00 |
| *Ca*. R. senegalensis | 1 | 0.42 | 0 | 0.00 |
| *Ca*. R. xinyangensis | 1 | 0.42 | 0 | 0.00 |
| **Protozoa** | 32 | 13.39 | 10 | 21.28 |
| ***Babesia*** | 13 | 5.44 | 5 | 10.64 |
| *B*. *begemina* | 7 | 2.93 | 0 | 0.00 |
| *B*. *bovis* | 4 | 1.67 | 1 | 2.13 |
| *B*. *microti* | 0 | 0.00 | 1 | 2.13 |
| *B*. *occultans* | 1 | 0.42 | 1 | 2.13 |
| *Babesia* spp. | 1 | 0.42 | 2 | 4.26 |
| ***Theileria*** | 16 | 6.69 | 5 | 10.64 |
| *T*. *annulata* | 3 | 1.26 | 1 | 2.13 |
| *T*. *buffeli* | 0 | 0.00 | 1 | 2.13 |
| *T*. *equi* | 1 | 0.42 | 0 | 0.00 |
| *T*. *luwenshumi* | 1 | 0.42 | 0 | 0.00 |
| *T*. *mutans* | 1 | 0.42 | 0 | 0.00 |
| *T*. *orientalis* | 4 | 1.67 | 1 | 2.13 |
| *T*. *ovis* | 1 | 0.42 | 0 | 0.00 |
| *T*. *sinensis* | 1 | 0.42 | 0 | 0.00 |
| *Theileria* spp. | 3 | 1.26 | 2 | 4.26 |
| *T*. *velifera* | 1 | 0.42 | 0 | 0.00 |
| ***Hepatozoon*** | 3 | 1.26 | 0 | 0.00 |
| *H*. *canis* | 2 | 0.84 | 0 | 0.00 |
| *Hepatozoon* spp. | 1 | 0.42 | 0 | 0.00 |
| **Viruses** | 36 | 15.06 | 5 | 10.64 |
| ***Orthonairovirus*** | 4 | 1.67 | 4 | 8.51 |
| Crimean-Congo hemorrhagic fever virus | 1 | 0.42 | 4 | 8.51 |
| Dugbe orthonairovirus | 1 | 0.42 | 0 | 0.00 |
| Nairobi sheep disease viruses | 1 | 0.42 | 0 | 0.00 |
| Meihua Mountain virus | 1 | 0.42 | 0 | 0.00 |
| ***Norwavirus*** | 1 | 0.42 | 0 | 0.00 |
| Beiji-nairovirus | 1 | 0.42 | 0 | 0.00 |
| ***Flavivirus*** | 11 | 4.60 | 0 | 0.00 |
| Bole tick virus | 3 | 1.26 | 0 | 0.00 |
| Flavi-like virus | 1 | 0.42 | 0 | 0.00 |
| Jingmen tick virus | 6 | 2.51 | 0 | 0.00 |
| Yanggou tick virus | 1 | 0.42 | 0 | 0.00 |
| ***Hepacivirus*** | 1 | 0.42 | 0 | 0.00 |
| Bovine hepacivirus | 1 | 0.42 | 0 | 0.00 |
| ***Parapoxvirus*** | 1 | 0.42 | 0 | 0.00 |
| Orf virus | 1 | 0.42 | 0 | 0.00 |
| ***Orthopoxvirus*** | 1 | 0.42 | 0 | 0.00 |
| Cowpox virus | 1 | 0.42 | 0 | 0.00 |
| ***Phlebovirus*** | 2 | 0.84 | 0 | 0.00 |
| Brown dog tick phlebovirus 1 | 1 | 0.42 | 0 | 0.00 |
| YN tick-associated phlebovirus 1 | 1 | 0.42 | 0 | 0.00 |
| ***Bandavirus*** | 2 | 0.84 | 0 | 0.00 |
| Severe fever with thrombocytopenia syndrome virus | 2 | 0.84 | 0 | 0.00 |
| **Uukuvirus** | 6 | 2.51 | 0 | 0.00 |
| Dabieshan tick virus | 2 | 0.84 | 0 | 0.00 |
| Lihan tick virus | 4 | 1.67 | 0 | 0.00 |
| **Mivirus** | 2 | 0.84 | 0 | 0.00 |
| Wuhan tick virus | 2 | 0.84 | 0 | 0.00 |
| **Rhabdovirus** | 1 | 0.42 | 0 | 0.00 |
| Rhabdo-like viruses | 1 | 0.42 | 0 | 0.00 |
| **Quaranjavirus** | 2 | 0.84 | 0 | 0.00 |
| Quaranjavirus | 2 | 0.84 | 0 | 0.00 |
| **Timovirus** | 2 | 0.84 | 0 | 0.00 |
| Antioquia tymovirus-like 1 | 1 | 0.42 | 0 | 0.00 |
| Antioquia tymovirus-like 2 | 1 | 0.42 | 0 | 0.00 |
| **Paslahepevirus** | 0 | 0.00 | 1 | 2.13 |
| Hepatitis E Virus | 0 | 0.00 | 1 | 2.13 |
| Total | 239 | 100.00 | 47 | 100.00 |

**References**

1. Ali A, Khan MA, Zahid H, Yaseen PM, Khan MQ, Nawab J, et al. Seasonal dynamics, record of ticks infesting humans, wild and domestic animals and molecular phylogeny of rhipicephalus microplus in Khyber Pakhtunkhwa Pakistan. Frontiers in Physiology. 2019;10:793.

2. Balasubramanian R, Yadav PD, Sahina S, Arathy Nadh V. Distribution and prevalence of ticks on livestock population in endemic area of Kyasanur forest disease in Western Ghats of Kerala, South India. Journal of parasitic diseases : official organ of the Indian Society for Parasitology. 2019;43(2):256-62.

3. Balinandi S, Chitimia-Dobler L, Grandi G, Nakayiki T, Kabasa W, Bbira J, et al. Morphological and molecular identification of ixodid tick species (Acari: Ixodidae) infesting cattle in Uganda. PARASITOLOGY RESEARCH. 2020;119(8):2411-20.

4. Li J, Chen Z-H, Jiang L, Wu C-Y, Liao S-Q, Lin X-H, et al. Characterization of cattle-origin ticks from Southern China. Acta Tropica. 2018;187:92-8.

5. da Gama BC, Martins TF, Labruna MB, da Costa Vieira RF, de Almeida JC. First report of Amblyomma sculptum (Amblyomma cajennense complex) in a Brazilian state classified as a silent area for human rickettsiosis. Veterinary World. 2023;16(11):2200-4.

6. De Clercq EM, Vanwambeke SO, Sungirai M, Adehan S, Lokossou R, Madder M. Geographic distribution of the invasive cattle tick Rhipicephalus microplus, a country-wide survey in Benin. EXPERIMENTAL AND APPLIED ACAROLOGY. 2012;58(4):441-52.

7. Farooqi SH, Ijaz M, Saleem MH, Rashid MI, Oneeb M, Khan A, et al. Distribution of Ixodid Tick Species and Associated Risk Factors in Temporal Zones of Khyber Pakhtunkhwa Province, Pakistan. PAKISTAN JOURNAL OF ZOOLOGY. 2017;49(6):2011-7.

8. Ullah H, Tabassum S, Ayaz S, Noreen S, Rehman AU, Akhtar N, et al. Diversity and Epidemiological Study of Hard Ticks Infesting Goats and Sheep of Hazara Division, Khyber Pakhtunkhwa, Pakistan. PAKISTAN JOURNAL OF ZOOLOGY. 2023;55(3):1031-40.

9. Diyes GCP, Rajakaruna RS. Diversity and distribution of tick species infesting goats with two new host records from Sri Lanka. JOURNAL OF THE NATIONAL SCIENCE FOUNDATION OF SRI LANKA. 2015;43(3):225-34.

10. Elango A, Shriram AN, Raju HK, Kumar A. Study on the Ixodid ticks species diversity (Acari: Ixodidae) on domestic ruminants in different agro-climatic regions of Tamil Nadu. TROPICAL ANIMAL HEALTH AND PRODUCTION. 2024;56(2):73.

11. Esculudis C, De Matos C, Cala A, Sungirai M, Madder M, Mapatse M. Morphological identification of ticks (Acari: Ixodidae) infesting donkeys (Equus asinus) in Maputo Province, Mozambique. EXPERIMENTAL AND APPLIED ACAROLOGY. 2022;86(2):257-69.

12. Etiang P, Musoba A, Nalumenya D, Ndekezi C, Bbira J, Ochwo S, et al. Distribution and prevalence of ixodid tick species (Acari: Ixodidae) infesting cattle in Karamoja region of northeastern Uganda. BMC VETERINARY RESEARCH. 2024;20(1):50.

13. Etiang P, Atim SA, Nkamwesiga J, Nalumenya D, Byaruhanga C, Odongo S, et al. Identification and distribution of Rhipicephalus microplus in selected high-cattle density districts in Uganda: signaling future demand for novel tick control approaches. BMC VETERINARY RESEARCH. 2024;20(1):119.

14. Ghafar A, Gasser RB, Rashid I, Ghafoor A, Jabbar A. Exploring the prevalence and diversity of bovine ticks in five agro-ecological zones of Pakistan using phenetic and genetic tools. Ticks and Tick-borne Diseases. 2020;11(5):101472.

15. Addo SO, Bentil RE, Baako BOA, Addae CA, Larbi JA, Baidoo PK, et al. First record of Rhipicephalus (Boophilus) microplus in Ghana, a potential risk to livestock production. Experimental & applied acarology. 2023;89(3-4):475-83.

16. Vongphayloth K, Brey PT, Robbins RG, Sutherland IW. First survey of the hard tick (Acari: Ixodidae) fauna of Nakai District, Khammouane Province, Laos, and an updated checklist of the ticks of Laos. SYSTEMATIC AND APPLIED ACAROLOGY. 2016;21(2):166-80.

17. Yawa M, Nyangiwe N, Jaja IF, Kadzere CT, Marufu MC. Geographic distribution of boophilid ticks in communal grazing cattle in the north-eastern region of the Eastern Cape Province, South Africa. Veterinary Parasitology: Regional Studies and Reports. 2021;23:100538.

18. Horak IG, Jordaan AJ, Nel PJ, van Heerden J, Heyne H, van Dalen EM. Distribution of endemic and introduced tick species in Free State Province, South Africa. JOURNAL OF THE SOUTH AFRICAN VETERINARY ASSOCIATION. 2015;86(1):1255.

19. Hussain N, Shabbir RMK, Ahmed H, Afzal MS, Ullah S, Ali A, et al. Prevalence of different tick species on livestock and associated equines and canine from different agro-ecological zones of Pakistan. Frontiers in Veterinary Science. 2023;9:1089999.

20. Intirach J, Lv X, Han Q, Lv Z-Y, Chen T. Morphological and Molecular Identification of Hard Ticks in Hainan Island, China. GENES. 2023;14(8).

21. Iqbal A, Sajid MS, Khan MN, Khan MK. Frequency distribution of hard ticks (Acari: Ixodidae) infesting bubaline population of district Toba Tek Singh, Punjab, Pakistan. Parasitology research. 2013;112(2):535-41.

22. Iqbal Z, Kayani AR, Akhter A, Qayyum M. Prevalence and Distribution of Hard Ticks and Their Associated Risk Factors in Sheep and Goats from Four Agro-Climatic Zones of Khyber Pakhtunkhwa (KPK), Pakistan. INTERNATIONAL JOURNAL OF ENVIRONMENTAL RESEARCH AND PUBLIC HEALTH. 2022;19(18).

23. Iqbal Z, Afshan K, Kayani AR, Ahmad H, Irfan M, Qayyum M. Epidemiology and Risk Mapping of hard ticks (Ixodidae) infecting Small Ruminants in Khyber Pakhtunkhwa Province, Pakistan. JOURNAL OF THE HELLENIC VETERINARY MEDICAL SOCIETY. 2023;74(1):5259-66.

24. Rafiq N, Kakar A, Ghani A, Iqbal A, Achakzai WM, Sadozai S, et al. Ixodid Ticks (Arachnida: Acari) Prevalence Associated with Risk Factors in the Bovine Host in District Quetta, Balochistan. PAKISTAN JOURNAL OF ZOOLOGY. 2017;49(6):2113-21.

25. Kanduma EG, Emery D, Githaka NW, Nguu EK, Bishop RP, Šlapeta J. Molecular evidence confirms occurrence of Rhipicephalus microplus Clade A in Kenya and sub-Saharan Africa. Parasites and Vectors. 2020;13(1):432.

26. Khan SS, Ahmed H, Afzal MS, Khan MR, Birtles RJ, Oliver JD. Epidemiology, Distribution and Identification of Ticks on Livestock in Pakistan. INTERNATIONAL JOURNAL OF ENVIRONMENTAL RESEARCH AND PUBLIC HEALTH. 2022;19(5).

27. Li L-H, Zhang Y, Wang J-Z, Li X-S, Yin S-Q, Zhu D, et al. High genetic diversity in hard ticks from a China-Myanmar border county. PARASITES & VECTORS. 2018;11(1):469.

28. Madder M, Adehan S, De Deken R, Adehan R, Lokossou R. New foci of Rhipicephalus microplus in West Africa. EXPERIMENTAL AND APPLIED ACAROLOGY. 2012;56(4):385-90.

29. Magesa WS, Haji I, Kinimi E, Nzalawahe JS, Kazwala R. Distribution and molecular identification of ixodid ticks infesting cattle in Kilombero and Iringa Districts, Tanzania. BMC VETERINARY RESEARCH. 2023;19(1):121.

30. Mamiro KA, Magwisha HB, Rukambile EJ, Ruheta MR, Kimboka EJ, Malulu DJ, Malele II. Occurrence of Ticks in Cattle in the New Pastoral Farming Areas in Rufiji District, Tanzania. Journal of veterinary medicine. 2016;2016:3420245-.

31. Nasreen N, Niaz S, Khan A, Ayaz S, Rashid M, Khattak I, et al. Molecular characterization of ticks infesting livestock in Khyber Pakhtunkhwa Province, Pakistan. INTERNATIONAL JOURNAL OF ACAROLOGY. 2020;46(3):165-70.

32. Kandi S, Chennuru S, Chitichoti J, Metta M, Krovvidi S. Morphological and molecular characterization of ticks infesting cattle and buffaloes in different agro-climatic zones in Andhra Pradesh, India, and factors associated with high tick prevalence. INTERNATIONAL JOURNAL OF ACAROLOGY. 2022;48(3):192-200.

33. Muhanguzi D, Byaruhanga J, Amanyire W, Ndekezi C, Ochwo S, Nkamwesiga J, et al. Invasive cattle ticks in East Africa: morphological and molecular confirmation of the presence of Rhipicephalus microplus in south-eastern Uganda. PARASITES & VECTORS. 2020;13(1):165.

34. Namgyal J, Lysyk TJ, Couloigner I, Checkley S, Gurung RB, Tenzin T, et al. Identification, Distribution, and Habitat Suitability Models of Ixodid Tick Species in Cattle in Eastern Bhutan. TROPICAL MEDICINE AND INFECTIOUS DISEASE. 2021;6(1).

35. Neves L, Afonso S, Horak IG. Ixodid ticks on dogs in southern Mozambique. The Onderstepoort journal of veterinary research. 2004;71(4):279-83.

36. Nyabongo L, Odongo DO, Milton G, Machuka E, Vudriko P, Pelle R, Kanduma EG. Molecular survey of cattle ticks in Burundi: First report on the presence of the invasive Rhipicephalus microplus tick. PLoS ONE. 2021;16(12 December 2021):e0261218.

37. Nyangiwe N, Goni S, Herve-Claude LP, Ruddat I, Horak IG. Ticks on pastures and on two breeds of cattle in the Eastern Cape province, South Africa. ONDERSTEPOORT JOURNAL OF VETERINARY RESEARCH. 2011;78(1):88-96.

38. Kebzai F, Ashraf K, Rehman MU, Akbar H, Avais M. Prevalence and associated risk factors of ixodid tick species infesting cattle and sheep in Balochistan, Pakistan. Veterinary Parasitology: Regional Studies and Reports. 2024;49:100993.

39. Rehman A, Nijhof AM, Sauter-Louis C, Schauer B, Staubach C, Conraths FJ. Distribution of ticks infesting ruminants and risk factors associated with high tick prevalence in livestock farms in the semiarid and arid agro-ecological zones of Pakistan. PARASITES & VECTORS. 2017;10(1):190.

40. Rooman M, Assad Y, Tabassum S, Sultan S, Ayaz S, Khan MF, et al. A cross-sectional survey of hard ticks and molecular characterization of Rhipicephalus microplus parasitizing domestic animals of Khyber Pakhtunkhwa, Pakistan. PLOS ONE. 2021;16(8):e0255138.

41. Sahara A, Nugraheni YR, Patra G, Prastowo J, Priyowidodo D. Ticks (Acari: Ixodidae) infestation on cattle in various regions in Indonesia. Veterinary world. 2019;12(11):1755-9.

42. Segura JA, Saldarriaga LJ, Cerón JM, Osorio LR, Rueda ZV, Gutiérrez LA. Hard tick species (Acari: Ixodidae) and infestation in two livestock agroecosystems from Antioquia, Colombia. Experimental & applied acarology. 2022;86(2):235-55.

43. Sungirai M, Abatih EN, Moyo DZ, De Clercq P, Madder M. Shifts in the distribution of ixodid ticks parasitizing cattle in Zimbabwe. MEDICAL AND VETERINARY ENTOMOLOGY. 2017;31(1):78-87.

44. Silatsa BA, Simo G, Githaka N, Mwaura S, Kamga RM, Oumarou F, et al. A comprehensive survey of the prevalence and spatial distribution of ticks infesting cattle in different agro-ecological zones of Cameroon. PARASITES & VECTORS. 2019;12(1):489.

45. Silatsa BA, Kuiate J-R, Njiokou F, Simo G, Feussom J-MK, Tunrayo A, et al. A countrywide molecular survey leads to a seminal identification of the invasive cattle tick Rhipicephalus Boophilus microplus in Cameroon, a decade after it was reported in Cote d'Ivoire. TICKS AND TICK-BORNE DISEASES. 2019;10(3):585-93.

46. Sultan S, Zeb J, Ayaz S, Rehman SU, Khan S, Hussain M, et al. Epidemiologic profile of hard ticks and molecular characterization of Rhipicephalus microplus infesting cattle in central part of Khyber Pakhtunkhwa, Pakistan. Parasitology research. 2022;121(9):2481-93.

47. Sungirai M, Madder M, Moyo DZ, De Clercq P, Abatih EN. An update on the ecological distribution of the Ixodidae ticks in Zimbabwe. EXPERIMENTAL AND APPLIED ACAROLOGY. 2015;66(2):269-80.

48. Charles R, Basu A, Sanford B, King-Cenac A, Melville-Edwin S, Pow-Brown P, et al. Survey of ticks of domestic dogs and cattle in three Caribbean islands. TRANSBOUNDARY AND EMERGING DISEASES. 2020;67:129-34.

49. Tawiah-Mensah CNL, Addo SO, Ansah-Owusu J, Abudu M, Malm ROT, Yartey KN, et al. Molecular identification of cattle ticks in the Greater Accra Region of Ghana: a high occurrence of Rhipicephalus microplus. Experimental & applied acarology. 2024;92(2):253-61.

50. Lamattina D, Tarragona EL, Costa SA, Guglielmone A, Nava S. Ticks (Acari: Ixodidae) of northern Misiones Province, Argentina. SYSTEMATIC AND APPLIED ACAROLOGY. 2014;19(4):393-8.

51. Mahlobo-Shwabede SIC, Zishiri OT, Thekisoe OMM, Bakkes D, Bohloa L, Molomo M, et al. Ticks of domestic animals in Lesotho: Morphological and molecular characterization. Veterinary parasitology, regional studies and reports. 2022;29:100691.

52. Yssouf A, Lagadec E, Bakari A, Foray C, Stachurski F, Cardinale E, et al. Colonization of Grande Comore Island by a lineage of Rhipicephalus appendiculatus ticks. PARASITES & VECTORS. 2011;4:38.

53. Zeb J, Song B, Senbill H, Aziz MU, Hussain S, Khan MA, et al. Ticks Infesting Dogs in Khyber Pakhtunkhwa, Pakistan: Detailed Epidemiological and Molecular Report. PATHOGENS. 2023;12(1).

54. Zimmermann NP, Rangel Aguirre AdA, Rodrigues VdS, Garcia MV, Medeiros JF, Zaidan Blecha IM, et al. Wildlife species, Ixodid fauna and new host records for ticks in an Amazon forest area, Rondonia, Brazil. REVISTA BRASILEIRA DE PARASITOLOGIA VETERINARIA. 2018;27(2):177-82.

55. Castellà J, Estrada-Peña A, Almería S, Ferrer D, Gutiérrez J, Ortuño A. A survey of ticks (Acari: Ixodidae) on dairy cattle on the island of Menorca in Spain. Experimental and Applied Acarology. 2001;25(10-11):899-908.

56. Barandika JF, Olmeda SA, Casado-Nistal MA, Hurtado A, Juste RA, Valcárcel F, et al. Differences in questing tick species distribution between Atlantic and Continental Climate regions in Spain. Journal of Medical Entomology. 2011;48(1):13-9.

57. Hornok S, Grima A, Takács N, Szekeres S, Kontschán J. First records and molecular-phylogenetic analyses of three tick species (Ixodes kaiseri, Hyalomma lusitanicum and Ornithodoros coniceps) from Malta. Ticks and Tick-borne Diseases. 2020;11(3):101379.

58. González J, Valcárcel F, Pérez-Sánchez JL, Tercero-Jaime JM, Olmeda AS. Seasonal dynamics of ixodid ticks on wild rabbits Oryctolagus cuniculus (Leporidae) from Central Spain. Experimental and Applied Acarology. 2016;70(3):369-80.

59. Lotfi D, Karima K. Identification and incidence of hard tick species during summer season 2019 in Jijel Province (northeastern Algeria). Journal of parasitic diseases : official organ of the Indian Society for Parasitology. 2021;45(1):211-7.

60. Hornok S, Cutajar B, Takacs N, Galea N, Attard D, Coleiro C, et al. On the way between Africa and Europe: Molecular taxonomy of ticks collected from birds in Malta. TICKS AND TICK-BORNE DISEASES. 2022;13(5):102001.

61. Zachée B, Mahamat O, Saboune M, Julius AN. Prevalence, intensity and risk factors of tick infestation of cattle in N’djamena Chad. Int J Vet Sci Anim Husb. 2020;5:139-46.

62. Vieira Lista MC, Belhassen-Garcia M, Vicente Santiago MB, Sanchez-Montejo J, Pedroza Perez C, Monsalve Arteaga LC, et al. Identification and Distribution of Human-Biting Ticks in Northwestern Spain. INSECTS. 2022;13(5).

63. Ali A, Shehla S, Zahid H, Ullah F, Zeb I, Ahmed H, et al. Molecular Survey and Spatial Distribution of Rickettsia spp. in Ticks Infesting Free-Ranging Wild Animals in Pakistan (2017–2021). Pathogens. 2022;11(2).

64. Ali A, Ullah S, Numan M, Almutairi MM, Alouffi A, Tanaka T. First report on tick-borne pathogens detected in ticks infesting stray dogs near butcher shops. Frontiers in Veterinary Science. 2023;10:1246871.

65. Ali A, Obaid MK, Almutairi MM, Alouffi A, Numan M, Ullah S, et al. Molecular detection of Coxiella spp. in ticks (Ixodidae and Argasidae) infesting domestic and wild animals: with notes on the epidemiology of tick-borne Coxiella burnetii in Asia. Frontiers in Microbiology. 2023;14.

66. Ali S, Hasan M, Ahmad AS, Ashraf K, Khan JA, Rashid MI. Molecular prevalence of Anaplasma marginale in ruminants and Rhipicephalus ticks in northern Pakistan. TROPICAL BIOMEDICINE. 2023;40(1):7-13.

67. Copa GN, Flores FS, Tarragona EL, Lamattina D, Sebastian PS, Gil JF, et al. Analysis of the tick communities associated to domestic mammals in rural areas of the Yungas montane forest from Argentina. Veterinary Parasitology: Regional Studies and Reports. 2023;39:100850.

68. Arroyave E, Cornwell ER, McBride JW, Diaz CA, Labruna MB, Rodas JD. Detection of tick-borne rickettsial pathogens in naturally infected dogs and dog-associated ticks in Medellin, Colombia. REVISTA BRASILEIRA DE PARASITOLOGIA VETERINARIA. 2020;29(3):e005320.

69. Balasubramanian R, Yadav PD, Sahina S, Nadh VA. The species distribution of ticks & the prevalence of Kyasanur forest disease virus in questing nymphal ticks from Western Ghats of Kerala, South India. INDIAN JOURNAL OF MEDICAL RESEARCH. 2021;154(5):743-9.

70. Tsai Y-L, Chomel BB, Chang C-C, Kass PH, Conrad PA, Chuang S-T. Bartonella and Babesia infections in cattle and their ticks in Taiwan. Comparative immunology, microbiology and infectious diseases. 2011;34(2):179-87.

71. Bermudez SE, Eremeeva ME, Karpathy SE, Samudio F, Zambrano ML, Zaldivar Y, et al. Detection and Identification of Rickettsial Agents in Ticks From Domestic Mammals in Eastern Panama. JOURNAL OF MEDICAL ENTOMOLOGY. 2009;46(4):856-61.

72. Gioia GV, Vinueza RL, Marsot M, Devillers E, Cruz M, Petit E, et al. Bovine anaplasmosis and tick-borne pathogens in cattle of the Galapagos Islands. TRANSBOUNDARY AND EMERGING DISEASES. 2018;65(5):1262-71.

73. Thanchomnang T, Rodpai R, Thinnabut K, Boonroumkaew P, Sadaow L, Tangkawanit U, et al. Characterization of the bacterial microbiota of cattle ticks in northeastern Thailand through 16S rRNA amplicon sequencing. INFECTION GENETICS AND EVOLUTION. 2023;115:105511.

74. Lu M, Ji Y, Zhao H, Wang W, Tian J, Duan C, et al. Circulation of multiple Rickettsiales bacteria in ticks from Sichuan province, Southwest China. Microbial Pathogenesis. 2023;183:106313.

75. Costa FB, da Costa AP, Moraes-Filho J, Martins TF, Soares HS, Ramirez DG, et al. Rickettsia amblyommatis infecting ticks and exposure of domestic dogs to Rickettsia spp. in an Amazon-Cerrado transition region of northeastern Brazil. PLOS ONE. 2017;12(6):e0179163.

76. Kasi KK, von Arnim F, Schulz A, Rehman A, Chudhary A, Oneeb M, et al. Crimean-Congo haemorrhagic fever virus in ticks collected from livestock in Balochistan, Pakistan. TRANSBOUNDARY AND EMERGING DISEASES. 2020;67(4):1543-52.

77. Daoduid OB, Eisenbarth A, Schulz A, Hartlaub J, Olopade JO, Oluwayelu DO, Groschup MH. Molecular detection of dugbe orthonairovirus in cattle and their infesting ticks Amblyomma and Rhipicephalus Boophilus)) in Nigeria. PLOS NEGLECTED TROPICAL DISEASES. 2021;15(11):e0009905.

78. Muraro LS, Nogueira MF, Borges AMCM, Souza AdO, Vieira TSWJ, de Aguiar DM. Detection of Ehrlichia sp. in Amblyomma sculptum parasitizing horses from Brazilian Pantanal wetland. Ticks and Tick-borne Diseases. 2021;12(3):101658.

79. Troyo A, Moreira-Soto RD, Calderon-Arguedas O, Mata-Somarribas C, Ortiz-Tello J, Barbieri ARM, et al. Detection of rickettsiae in fleas and ticks from areas of Costa Rica with history of spotted fever group rickettsioses. TICKS AND TICK-BORNE DISEASES. 2016;7(6):1128-34.

80. da Silveira JAG, Rabelo EML, Ribeiro MFB. Detection of Theileria and Babesia in brown brocket deer (Mazama gouazoubira) and marsh deer (Blastocerus dichotomus) in the State of Minas Gerais, Brazil. Veterinary parasitology. 2011;177(1-2):61-6.

81. Dzul-Rosado KR, Arroyo-Solis KA, Torres-Monroy AJ, Arias-Leon JJ, Peniche-Lara GF, Puerto-Manzano FI, et al. Tick-associated diseases identified from hunting dogs during the COVID-19 pandemic in a Mayan community in Yucatan, Mexico. OPEN VETERINARY JOURNAL. 2023;13(6):794-800.

82. Rehman A, Conraths FJ, Sauter-Louis C, Krücken J, Nijhof AM. Epidemiology of tick-borne pathogens in the semi-arid and the arid agro-ecological zones of Punjab province, Pakistan. Transboundary and Emerging Diseases. 2019;66(1):526-36.

83. Lu M, Tian J-H, Yu B, Guo W-P, Holmes EC, Zhang Y-Z. Extensive diversity of rickettsiales bacteria in ticks from Wuhan, China. Ticks and Tick-borne Diseases. 2017;8(4):574-80.

84. Ferrell AM, Brinkerhoff RJ, Bernal J, Bermúdez SE. Ticks and tick-borne pathogens of dogs along an elevational and land-use gradient in Chiriquí province, Panamá. Experimental & applied acarology. 2017;71(4):371-85.

85. Chiang PS, Lai YW, Chung HH, Chia YT, Wang CC, Teng HJ, Chen SL. First molecular detection of a novel Babesia species from Haemaphysalis hystricis in Taiwan. Ticks and Tick-borne Diseases. 2024;15(1):102284.

86. Flores-Mendoza C, Florin D, Felices V, Pozo EJ, Graf PCF, Burrus RG, Richards AL. Detection of Rickettsia parkeri from within Piura, Peru, and the First Reported Presence of Candidatus Rickettsia andeanae in the Tick Rhipicephalus sanguineus. VECTOR-BORNE AND ZOONOTIC DISEASES. 2013;13(7):505-8.

87. Galay RL, Talactac MR, Ambita-Salem BV, Chu DMM, dela Costa LMO, Salangsang CMA, et al. Molecular detection of Rickettsia spp. And Coxiella burnetii in cattle, water buffalo, and Rhipicephalus (Boophilus) microplus ticks in Luzon Island of the Philippines. Tropical Medicine and Infectious Disease. 2020;5(2).

88. Galay RL, Llaneta CR, Monreal MKFB, Armero AL, Baluyut ABD, Regino CMF, et al. Molecular Prevalence of Anaplasma marginale and Ehrlichia in Domestic Large Ruminants and Rhipicephalus Boophilus microplus Ticks From Southern Luzon, Philippines. FRONTIERS IN VETERINARY SCIENCE. 2021;8:746705.

89. Niu Q, Liu Z, Yang J, Yu P, Pan Y, Zhai B, et al. Genetic diversity and molecular characterization of Babesia motasi-like in small ruminants and ixodid ticks from China. Infection, Genetics and Evolution. 2016;41:8-15.

90. Thinnabut K, Rodpai R, Sanpool O, Maleewong W, Tangkawanit U. Genetic diversity of tick (Acari: Ixodidae) populations and molecular detection of Anaplasma and Ehrlichia infesting beef cattle from upper-northeastern Thailand. Infection, Genetics and Evolution. 2023;107:105394.

91. Wang G, Tian X, Peng R, Huang Y, Li Y, Li Z, et al. Genomic and phylogenetic profiling of RNA of tick-borne arboviruses in Hainan Island, China. Microbes and Infection. 2024;26(1):105218.

92. Li W, Li R, Tang X, Cheng J, Zhan L, Shang Z, Wu J. Genomics evolution of Jingmen viruses associated with ticks and vertebrates. GENOMICS. 2023;115(6):110734.

93. Ghafar A, Khan A, Cabezas-Cruz A, Gauci CG, Niaz S, Ayaz S, et al. An Assessment of the Molecular Diversity of Ticks and Tick-Borne Microorganisms of Small Ruminants in Pakistan. MICROORGANISMS. 2020;8(9).

94. Ghafar A, Cabezas-Cruz A, Galon C, Obregon D, Gasser RB, Moutailler S, Jabbar A. Bovine ticks harbour a diverse array of microorganisms in Pakistan. PARASITES & VECTORS. 2020;13(1):1.

95. Guo WP, Zhang B, Wang YH, Xu G, Wang X, Ni X, Zhou EM. Molecular identification and characterization of Anaplasma capra and Anaplasma platys-like in Rhipicephalus microplus in Ankang, Northwest China. BMC Infectious Diseases. 2019;19(1):434.

96. Sumrandee C, Baimai V, Trinachartvanit W, Ahantarig A. Hepatozoon and Theileria species detected in ticks collected from mammals and snakes in Thailand. Ticks and Tick-borne Diseases. 2015;6(3):309-15.

97. Hernandez SAV, Salamat SEA, Galay RL. Analysis of the bacterial community in female Rhipicephalus microplus ticks from selected provinces in Luzon, Philippines, using next-generation sequencing. Experimental & applied acarology. 2023;91(3):463-75.

98. Hou J, Ling F, Chai C, Lu Y, Yu X, Lin J, et al. Prevalence of Borrelia burgdorferi Sensu Lato in Ticks from Eastern China. AMERICAN JOURNAL OF TROPICAL MEDICINE AND HYGIENE. 2015;92(2):262-6.

99. Lu M, Tian J, Pan X, Qin X, Wang W, Chen J, et al. Identification of Rickettsia spp., Anaplasma spp., and an Ehrlichia canis-like agent in Rhipicephalus microplus from Southwest and South-Central China. Ticks and Tick-borne Diseases. 2022;13(2):101884.

100. Wang Y-N, Jiang R-R, Ding H, Zhang X-L, Wang N, Zhang Y-F, et al. First Detection of Mukawa Virus in Ixodes persulcatus and Haemaphysalis concinna in China. FRONTIERS IN MICROBIOLOGY. 2022;13:791563.

101. Jamil A, Yu Z, Wang Y, Xin Q, Gao S, Wahab MA, et al. Tick-borne Rickettsia, Anaplasma, Theileria, and enzootic nasal tumor virus in ruminant, PET, and poultry animals in Pakistan. FRONTIERS IN MICROBIOLOGY. 2024;15:1359492.

102. Jiao J, Zhang J, He P, OuYang X, Yu Y, Wen B, et al. Identification of Tick-Borne Pathogens and Genotyping of Coxiella burnetii in Rhipicephalus microplus in Yunnan Province, China. Frontiers in Microbiology. 2021;12:736484.

103. Karim S, Budachetri K, Mukherjee N, Williams J, Kausar A, Hassan MJ, et al. A study of ticks and tick-borne livestock pathogens in Pakistan. PLOS NEGLECTED TROPICAL DISEASES. 2017;11(6):e0005681.

104. Khan Z, Shehla S, Alouffi A, Obaid MK, Khan AZ, Almutairi MM, et al. Molecular Survey and Genetic Characterization of Anaplasma marginale in Ticks Collected from Livestock Hosts in Pakistan. Animals. 2022;12(13).

105. Khan M, Almutairi MM, Alouffi A, Tanaka T, Chang SC, Chen CC, Ali A. Molecular evidence of Borrelia theileri and closely related Borrelia spp. in hard ticks infesting domestic animals. Frontiers in Veterinary Science. 2023;10:1297928.

106. Kho K-L, Koh F-X, Jaafar T, Nizam QNH, Tay S-T. Prevalence and molecular heterogeneity of Bartonella bovis in cattle and Haemaphysalis bispinosa ticks in Peninsular Malaysia. BMC VETERINARY RESEARCH. 2015;11:153.

107. Kobayashi T, Chatanga E, Qiu Y, Simuunza M, Kajihara M, Hang’ombe BM, et al. Molecular detection and genotyping of coxiella-like endosymbionts in ticks collected from animals and vegetation in Zambia. Pathogens. 2021;10(6).

108. Li LH, Wang JZ, Zhu D, Li XS, Lu Y, Yin SQ, et al. Detection of novel piroplasmid species and Babesia microti and Theileria orientalis genotypes in hard ticks from Tengchong County, Southwest China. Parasitology Research. 2020;119(4):1259-69.

109. Lu M, Meng C, Gao X, Sun Y, Zhang J, Tang G, et al. Diversity of Rickettsiales in Rhipicephalus microplus Ticks Collected in Domestic Ruminants in Guizhou Province, China. Pathogens. 2022;11(10).

110. Lu M, Tian J, Wang W, Zhao H, Jiang H, Han J, et al. High diversity of Rickettsia spp., Anaplasma spp., and Ehrlichia spp. in ticks from Yunnan Province, Southwest China. Frontiers in Microbiology. 2022;13:1008110.

111. Lu M, Meng C, Zhang B, Wang X, Tian J, Tang G, et al. Prevalence of Spotted Fever Group Rickettsia and Candidatus Lariskella in Multiple Tick Species from Guizhou Province, China. Biomolecules. 2022;12(11).

112. Mahlobo-Shwabede SIC, Zishiri OT, Thekisoe OMM, Makalo MJR. Molecular detection of coxiella burnetii, rickettsia africae and anaplasma species in ticks from domestic animals in lesotho. Pathogens. 2021;10(9).

113. Makenov MT, Toure AH, Korneev MG, Sacko N, Porshakov AM, Yakovlev SA, et al. Rhipicephalus microplus and its vector-borne haemoparasites in Guinea: further species expansion in West Africa. Parasitology research. 2021;120(5):1563-70.

114. Matsimbe AM, Magaia V, Sanches GS, Neves L, Noormahomed E, Antunes S, Domingos A. Molecular detection of pathogens in ticks infesting cattle in Nampula province, Mozambique. EXPERIMENTAL AND APPLIED ACAROLOGY. 2017;73(1):91-102.

115. Gómez GF, Isaza JP, Segura JA, Alzate JF, Gutiérrez LA. Metatranscriptomic virome assessment of Rhipicephalus microplus from Colombia. Ticks and Tick-borne Diseases. 2020;11(5):101426.

116. Misra BR, Kumar N, Kant R, Deval H, Singh R, Pandey AK, et al. Abundance of Ticks (Acari: Ixodidae) and Presence of Rickettsia and Anaplasma in Ticks Infesting Domestic Animals From Northern India. JOURNAL OF MEDICAL ENTOMOLOGY. 2021;58(3):1370-5.

117. Intirach J, Lv X, Sutthanont N, Cai B, Champakaew D, Chen T, et al. Molecular and next-generation sequencing analysis of tick-borne pathogens of Rhipicephalus ticks (Acari: Ixodidae) in cattle and dogs. Acta Tropica. 2024;252:107138.

118. Aung A, Kaewlamun W, Narapakdeesakul D, Poofery J, Kaewthamasorn M. Molecular detection and characterization of tick-borne parasites in goats and ticks from Thailand. Ticks and Tick-borne Diseases. 2022;13(3):101938.

119. Santos CAd, Suzin A, Vogliotti A, Nunes PH, Barbieri ARM, Labruna MB, et al. Molecular detection of a Borrelia sp. in nymphs of Amblyomma brasiliense ticks (Acari: Ixodidae) from Iguaçu National Park, Brazil, genetically related to Borrelia from Ethiopia and Côte d’Ivoire. Ticks and Tick-borne Diseases. 2020;11(6):101519.

120. Rialch A, Sankar M, Silamparasan M, Madhusoodan AP, Kharayat NS, Gautam S, et al. Molecular detection of Coxiella-like endosymbionts in Rhipicephalus microplus from north India. Veterinary Parasitology: Regional Studies and Reports. 2022;36:100803.

121. López-Pérez AM, Sánchez-Montes S, Maya-Badillo BA, Orta-Pineda G, Reveles-Félix S, Becker I, et al. Molecular detection of Rickettsia amblyommatis and Rickettsia parkeri in ticks collected from wild pigs in Campeche, Mexico. Ticks and Tick-borne Diseases. 2022;13(1):101844.

122. Ehlers J, Krüger A, Rakotondranary SJ, Ratovonamana RY, Poppert S, Ganzhorn JU, Tappe D. Molecular detection of Rickettsia spp., Borrelia spp., Bartonella spp. and Yersinia pestis in ectoparasites of endemic and domestic animals in southwest Madagascar. Acta Tropica. 2020;205:105339.

123. Sumrandee C, Baimai V, Trinachartvanit W, Ahantarig A. Molecular detection of Rickettsia, Anaplasma, Coxiella and Francisella bacteria in ticks collected from Artiodactyla in Thailand. Ticks and Tick-borne Diseases. 2016;7(5):678-89.

124. Ojeda-Chi MM, Rodriguez-Vivas RI, Esteve-Gasent MD, Pérez de León A, Modarelli JJ, Villegas-Perez S. Molecular detection of rickettsial tick-borne agents in white-tailed deer (Odocoileus virginianus yucatanensis), mazama deer (Mazama temama), and the ticks they host in Yucatan, Mexico. Ticks and Tick-borne Diseases. 2019;10(2):365-70.

125. Guo W-P, Wang Y-H, Lu Q, Xu G, Luo Y, Ni X, Zhou E-M. Molecular detection of spotted fever group rickettsiae in hard ticks, northern China. TRANSBOUNDARY AND EMERGING DISEASES. 2019;66(4):1587-96.

126. Martinez Diaz H-C, Gil-Mora J, Betancourt-Ruiz P, Silva-Ramos CR, Matiz-Gonzalez JM, Villalba-Perez M-A, et al. Molecular detection of tick-borne rickettsial pathogens in ticks collected from domestic animals from Cauca, Colombia. Acta tropica. 2023;238:106773.

127. Yu P-F, Niu Q-L, Liu Z-J, Yang J-F, Chen Z, Guan G-Q, et al. Molecular epidemiological surveillance to assess emergence and re-emergence of tick-borne infections in tick samples from China evaluated by nested PCRs. Acta Tropica. 2016;158:181-8.

128. Yuan T-T, Du C-H, Xia L-Y, Que T-C, von Fricken ME, Jiang B-G, et al. Molecular evidence of Candidatus Rickettsia longicornii and a novel Rickettsia strain from ticks in Southern China. Ticks and Tick-borne Diseases. 2021;12(3):101679.

129. Roy BC, Krücken J, Ahmed JS, Majumder S, Baumann MP, Clausen PH, Nijhof AM. Molecular identification of tick-borne pathogens infecting cattle in Mymensingh district of Bangladesh reveals emerging species of Anaplasma and Babesia. Transboundary and Emerging Diseases. 2018;65(2):e231-e42.

130. Segura JA, Dibernardo A, Manguiat K, Waitt B, Rueda ZV, Keynan Y, et al. Molecular surveillance of microbial agents from cattle-attached and questing ticks from livestock agroecosystems of Antioquia, Colombia. Comparative Immunology, Microbiology and Infectious Diseases. 2024;105:102113.

131. Mohanta UK, Marguerite MP, Ji S, Ma Z, Li H, El-Sayed SAES, et al. Molecular survey of canine tick-borne pathogens in ticks and stray dogs in Dhaka city, Bangladesh. Parasitology International. 2024;100:102860.

132. Molina-Garza ZJ, Cuesy-Leon M, Baylon-Pacheco L, Rosales-Encina JL, Galaviz-Silva L. Diversity of midgut microbiota in ticks collected from white-tailed deer (Odocoileus virginianus) from northern Mexico. PARASITES HOSTS AND DISEASES. 2024;62(1):117-30.

133. Neves LC, Paula WVdF, de Paula LGF, da Silva BBF, Dias SA, Pereira BG, et al. Detection of <i>Rickettsia</i> spp. in Animals and Ticks in Midwestern Brazil, Where Human Cases of Rickettsiosis Were Reported. ANIMALS. 2023;13(8).

134. Ngnindji-Youdje Y, Diarra AZ, Lontsi-Demano M, Tchuinkam T, Parola P. Detection of Tick-Borne Pathogens in Ticks from Cattle in Western Highlands of Cameroon. Microorganisms. 2022;10(10).

135. Nimisha M, Devassy JK, Pradeep RK, Pakideery V, Sruthi MK, Pious A, et al. Ticks and accompanying pathogens of domestic and wild animals of Kerala, South India. Experimental & applied acarology. 2019;79(1):137-55.

136. Nogueira RdMS, Silva AB, Sato TP, Sá JCd, Santos ACGd, Amorim Filho EF, et al. Molecular and serological detection of Theileria equi, Babesia caballi and Anaplasma phagocytophilum in horses and ticks in Maranhão, Brazil. Pesquisa Veterinária Brasileira. 2017;37(12):1416-22.

137. Osorio M, Miranda J, Gonzalez M, Mattar S. Anaplasma sp., Ehrlichia sp., and Rickettsia sp in Ticks: A High Risk for Public Health in Ibague, Colombia. KAFKAS UNIVERSITESI VETERINER FAKULTESI DERGISI. 2018;24(4):557-62.

138. Ouedraogo AS, Zannou OM, Biguezoton AS, Kouassi PY, Belem A, Farougou S, et al. Cattle ticks and associated tick-borne pathogens in Burkina Faso and Benin: Apparent northern spread of Rhipicephalus microplus in Benin and first evidence of Theileria velifera and Theileria annulata. TICKS AND TICK-BORNE DISEASES. 2021;12(4):101733.

139. Paguem A, Manchang K, Kamtsap P, Renz A, Schaper S, Dobler G, et al. Ticks and Rickettsiae Associated with Wild Animals Sold in Bush Meat Markets in Cameroon. PATHOGENS. 2023;12(2).

140. Pang Z, Jin Y, Pan M, Zhang Y, Wu Z, Liu L, Niu G. Geographical distribution and phylogenetic analysis of Jingmen tick virus in China. iScience. 2022;25(9):105007.

141. Parola P, Cornet JP, Sanogo YO, Miller RS, Thien HV, Gonzalez JP, et al. Detection of Ehrlichia spp., Anaplasma spp., Rickettsia spp., and other eubacteria in ticks from the Thai-Myanmar border and Vietnam. JOURNAL OF CLINICAL MICROBIOLOGY. 2003;41(4):1600-8.

142. Pesquera C, Portillo A, Palomar AM, Oteo JA. Investigation of tick-borne bacteria Rickettsia spp., Anaplasma spp., Ehrlichia spp. and Borrelia spp.) in ticks collected from Andean tapirs, cattle and vegetation from a protected area in Ecuador. PARASITES & VECTORS. 2015;8:46.

143. Phiri BSJ, Kattner S, Chitimia-Dobler L, Woelfel S, Albanus C, Dobler G, Kuepper T. Rickettsia spp. in Ticks of South Luangwa Valley, Eastern Province, Zambia. MICROORGANISMS. 2023;11(1).

144. Polsomboon S, Hoel DF, Murphy JR, Linton YM, Motoki M, Robbins RG, et al. Molecular Detection and Identification of Rickettsia Species in Ticks (Acari: Ixodidae) Collected From Belize, Central America. Journal of medical entomology. 2017;54(6):1718-26.

145. Chu C, Jiang B-G, Liu W, Zhao Q-M, Wu X-M, Zhang P-H, et al. Presence of pathogenic Borrelia burgdorferi sensu lato in ticks and rodents in Zhejiang, south-east China. JOURNAL OF MEDICAL MICROBIOLOGY. 2008;57(8):980-5.

146. Pothmann D, Poppert S, Rakotozandrindrainy R, Hogan B, Mastropaolo M, Thiel C, Silaghi C. Prevalence and genetic characterization of Anaplasma marginale in zebu cattle (Bos indicus) and their ticks (Amblyomma variegatum, Rhipicephalus microplus) from Madagascar. Ticks and Tick-borne Diseases. 2016;7(6):1116-23.

147. Shahid MF, Yaqub T, Ali M, Ul-Rahman A, Bente DA. Prevalence and phylogenetic analysis of Crimean-Congo hemorrhagic fever virus in ticks collected from Punjab province of Pakistan. Acta Tropica. 2021;218:105892.

148. Qi Y, Ai L, Jiao J, Wang J, Wu D, Wang P, et al. High prevalence of Rickettsia spp. in ticks from wild hedgehogs rather than domestic bovine in Jiangsu province, Eastern China. FRONTIERS IN CELLULAR AND INFECTION MICROBIOLOGY. 2022;12:954785.

149. Qiu Y, Nakao R, Thu MJ, Akter S, Alam MZ, Kato S, et al. Molecular evidence of spotted fever group rickettsiae and Anaplasmataceae from ticks and stray dogs in Bangladesh. Parasitology Research. 2016;115(3):949-55.

150. de Sousa KCM, Herrera HM, Rocha FL, Costa FB, Martins TF, Labruna MB, et al. Rickettsia spp. among wild mammals and their respective ectoparasites in Pantanal wetland, Brazil. Ticks and Tick-borne Diseases. 2018;9(1):10-7.

151. Sajid MS, Iqbal A, Rizwan HM, Kausar A, Bin Tahir U, Younus M, et al. Guardians of the Herd: Molecular Surveillance of Tick Vectors Uncovers Theileriosis Perils in Large Ruminants. MICROORGANISMS. 2023;11(11).

152. Shehla S, Almutairi MM, Alouffi A, Tanaka T, Chang SC, Chen CC, Ali A. Molecular Survey of Rickettsia raoultii in Ticks Infesting Livestock from Pakistan with Notes on Pathogen Distribution in Palearctic and Oriental Regions. Veterinary Sciences. 2023;10(11).

153. Shi J, Shen S, Wu H, Zhang Y, Deng F. Metagenomic Profiling of Viruses Associated with Rhipicephalus microplus Ticks in Yunnan Province, China. VIROLOGICA SINICA. 2021;36(4):623-35.

154. Solomon A, Tanga BM. The First Investigation of Tick Vectors and Tick-Borne Diseases in Extensively Managed Cattle in Alle District, Southwestern Ethiopia. VETERINARY MEDICINE INTERNATIONAL. 2020;2020:8862289.

155. Alam S, Khan M, Alouffi A, Almutairi MM, Ullah S, Numan M, et al. Spatio-Temporal Patterns of Ticks and Molecular Survey of Anaplasma marginale, with Notes on Their Phylogeny. MICROORGANISMS. 2022;10(8).

156. Arrais RC, Paula RC, Martins TF, Nieri-Bastos FA, Marcili A, Labruna MB. Survey of ticks and tick-borne agents in maned wolves (Chrysocyon brachyurus) from a natural landscape in Brazil. Ticks and Tick-borne Diseases. 2021;12(2):101639.

157. Sousa ACP, Suzin A, da Silva Rodrigues V, Rezende LM, da Costa Maia R, Vieira RBK, Szabó MPJ. Ticks (Acari: Ixodidae) and rickettsiae associated with wild boars in a rural area of Minas Gerais, Brazil. Veterinary Parasitology: Regional Studies and Reports. 2024;50:101016.

158. Oundo JW, Kalayou S, Bosch QT, Villinger J, Koenraadt CJM, Masiga D. Ticks (Acari: Ixodidae) infesting cattle in coastal Kenya harbor a diverse array of tick-borne pathogens. Ticks and Tick-borne Diseases. 2024;15(1):102266.

159. Szabó MPJ, Pascoal JO, Martins MM, Ramos VdN, Osava CF, Santos ALQ, et al. Ticks and Rickettsia on anteaters from Southeast and Central-West Brazil. Ticks and Tick-borne Diseases. 2019;10(3):540-5.

160. Labruna MB, Martins TF, Acosta ICL, Serpa MCA, Soares HS, Teixeira RHF, et al. Ticks and rickettsial exposure in lowland tapirs (Tapirus terrestris) of three Brazilian biomes. Ticks and Tick-borne Diseases. 2021;12(3):101648.

161. Teglas M, Matern E, Lein S, Foley P, Mahan SM, Foley J. Ticks and tick-borne disease in Guatemalan cattle and horses. Veterinary parasitology. 2005;131(1-2):119-27.

162. Tonetti N, Berggoetz M, Rühle C, Pretorius AM, Gern L. Ticks and tick-borne pathogens from wildlife in the Free State Province, South Africa. Journal of wildlife diseases. 2009;45(2):437-46.

163. Romero LE, Binder LC, Marcili A, Labruna MB. Ticks and tick-borne rickettsiae from dogs in El Salvador, with report of the human pathogen Rickettsia parkeri. TICKS AND TICK-BORNE DISEASES. 2023;14(5):102206.

164. Ojeda-Chi MM, Rodriguez-Vivas RI, Esteve-Gasent MD, Pérez de León AA, Modarelli JJ, Villegas-Perez SL. Ticks infesting dogs in rural communities of Yucatan, Mexico and molecular diagnosis of rickettsial infection. Transboundary and Emerging Diseases. 2019;66(1):102-10.

165. Velusamy R, Ponnudurai G, Alagesan A, Rani N, Kolte SW, Rubinibala B. Epidemiology and molecular characterization of Theileria annulata in ticks collected from cattle in the central part of Tamil Nadu, India. Parasitology Research. 2023;122(12):3077-86.

166. Molina-Hoyos K, Montoya-Ruiz C, Aguilar PV, Perez-Doria A, Diaz FJ, Rodas JD. Virome analyses of Amblyomma cajennense and Rhipicephalus microplus ticks collected in Colombia. Acta tropica. 2024;253:107158-.

167. Orozco Orozco M, Gómez GF, Alzate JF, Isaza JP, Gutiérrez LA. Virome analysis of three Ixodidae ticks species from Colombia: A potential strategy for discovering and surveying tick-borne viruses. Infection, Genetics and Evolution. 2021;96:105103.

168. Wang S, Li J, Niu G, Wang X, Ding S, Jiang X, et al. SFTS Virus in Ticks in an Endemic Area of China. AMERICAN JOURNAL OF TROPICAL MEDICINE AND HYGIENE. 2015;92(4):684-9.

169. Wang A, Pang Z, Liu L, Ma Q, Han Y, Guan Z, et al. Detection and Phylogenetic Analysis of a Novel Tick-Borne Virus in Yunnan and Guizhou Provinces, Southwestern China. PATHOGENS. 2021;10(9).

170. Wang Q, Guo W-B, Pan Y-S, Jiang B-G, Du C-H, Que T-C, et al. Detection of Novel Spotted Fever Group Rickettsiae (Rickettsiales: Rickettsiaceae) in Ticks (Acari: Ixodidae) in Southwestern China. JOURNAL OF MEDICAL ENTOMOLOGY. 2021;58(3):1363-9.

171. Weaver GV, Anderson N, Garrett K, Thompson AT, Yabsley MJ. Ticks and Tick-Borne Pathogens in Domestic Animals, Wild Pigs, and Off-Host Environmental Sampling in Guam, USA. Frontiers in Veterinary Science. 2021;8:803424.

172. Xiang Y, Zhou J, Yu F, Zhang Y, Li S, Hu Y, et al. Characterization of bacterial communities in ticks parasitizing cattle in a touristic location in southwestern China. EXPERIMENTAL AND APPLIED ACAROLOGY. 2023;90(1-2):119-35.

173. Xu L, Guo M, Hu B, Zhou H, Yang W, Hui L, et al. Tick virome diversity in Hubei Province, China, and the influence of host ecology. VIRUS EVOLUTION. 2021;7(2):veab089.

174. Xu J, Gu XL, Jiang ZZ, Cao XQ, Wang R, Peng QM, et al. Pathogenic Rickettsia, Anaplasma, and Ehrlichia in Rhipicephalus microplus ticks collected from cattle and laboratory hatched tick larvae. PLoS Neglected Tropical Diseases. 2023;17(8 August):e0011546.

175. Yang Z, Wang H, Yang S, Wang X, Shen Q, Ji L, et al. Virome diversity of ticks feeding on domestic mammals in China. VIROLOGICA SINICA. 2023;38(2):208-21.

176. Ybanez AP, Sivakumar T, Ybanez RHD, Ratilla JC, Perez ZO, Gabotero SR, et al. First Molecular Characterization of Anaplasma marginale in Cattle and Rhipicephalus (Boophilus) microplus Ticks in Cebu, Philippines. JOURNAL OF VETERINARY MEDICAL SCIENCE. 2013;75(1):27-36.

177. Yssouf A, Socolovschi C, Kernif T, Temmam S, Lagadec E, Tortosa P, Parola P. First molecular detection of Rickettsia africae in ticks from the Union of the Comoros. PARASITES & VECTORS. 2014;7:444.

178. Yuan S, Yao X-Y, Lian C-Y, Kong S, Shao J-W, Zhang X-L. Molecular detection and genetic characterization of bovine hepacivirus identified in ticks collected from cattle in Harbin, northeastern China. FRONTIERS IN VETERINARY SCIENCE. 2023;10:1093898.

179. Zeb J, Szekeres S, Takács N, Kontschán J, Shams S, Ayaz S, Hornok S. Genetic diversity, piroplasms and trypanosomes in Rhipicephalus microplus and Hyalomma anatolicum collected from cattle in northern Pakistan. Experimental & applied acarology. 2019;79(2):233-43.

180. Zhang X, Li H-Y, Shao J-W, Pei M-C, Cao C, Huang F-Q, Sun M-F. Genomic characterization and phylogenetic analysis of a novel Nairobi sheep disease genogroup Orthonairovirus from ticks, Southeastern China. FRONTIERS IN MICROBIOLOGY. 2022;13:977405.

181. Zhang X-L, Deng Y-P, Yang T, Li L-Y, Cheng T-Y, Liu G-H, Duan D-Y. Metagenomics of the midgut microbiome of Rhipicephalus microplus from China. Parasites & vectors. 2022;15(1):48.

182. Zhang X, Lv W, Teng Z, Zhao N, Zhou Y, Ma D, et al. Molecular detection of Rickettsiales and a potential novel Ehrlichia species closely related to Ehrlichia chaffeensis in ticks (Acari: Ixodidae) from Shaanxi Province, China, in 2022 to 2023. Frontiers in Microbiology. 2023;14:1331434.

183. Zhao N, Pan K, Teng Z, Wang H, Zhang X, Ren H, et al. Molecular detection reveals diverse tick-borne bacterial and protozoan pathogens in two tick species from Yingshan County of Hubei Province, China in 2021–2022. Frontiers in Microbiology. 2023;14:1298037.

184. Blanda V, Torina A, La Russa F, D'Agostino R, Randazzo K, Scimeca S, et al. A retrospective study of the characterization of Rickettsia species in ticks collected from humans. TICKS AND TICK-BORNE DISEASES. 2017;8(4):610-4.

185. Aubry C, Socolovschi C, Raoult D, Parola P. Bacterial agents in 248 ticks removed from people from 2002 to 2013. Ticks and Tick-borne Diseases. 2016;7(3):475-81.

186. Cajimat MNB, Rodriguez SE, Schuster IUE, Swetnam DM, Ksiazek TG, Habela MA, et al. Genomic Characterization of Crimean-Congo Hemorrhagic Fever Virus in Hyalomma Tick from Spain, 2014. VECTOR-BORNE AND ZOONOTIC DISEASES. 2017;17(10):714-9.

187. Chisu V, Foxi C, Masala G. First molecular detection of the human pathogen Rickettsia raoultii and other spotted fever group rickettsiae in Ixodid ticks from wild and domestic mammals. Parasitology Research. 2018;117(11):3421-9.

188. Chisu V, Foxi C, Masala G. First molecular detection of Francisella-like endosymbionts in Hyalomma and Rhipicephalus tick species collected from vertebrate hosts from Sardinia island, Italy. Experimental and Applied Acarology. 2019;79(2):245-54.

189. Milhano N, Carvalho ILD, Alves AS, Arroube S, Soares J, Rodriguez P, et al. Coinfections of Rickettsia slovaca and Rickettsia helvetica with Borrelia lusitaniae in ticks collected in a Safari Park, Portugal. Ticks and Tick-borne Diseases. 2010;1(4):172-7.

190. Elhachimi L, Rogiers C, Casaert S, Fellahi S, Van Leeuwen T, Dermauw W, et al. Ticks and Tick-Borne Pathogens Abound in the Cattle Population of the Rabat-Sale Kenitra Region, Morocco. PATHOGENS. 2021;10(12).

191. Fernández N, Revuelta B, Aguilar I, Soares JF, Zintl A, Gray J, et al. Babesia and Theileria Identification in Adult Ixodid Ticks from Tapada Nature Reserve, Portugal. Pathogens. 2022;11(2).

192. Gonzalez J, Gonzalez MG, Valcarcel F, Sanchez M, Martin-Hernandez R, Maria Tercero J, Sonia Olmeda A. Prevalence of Coxiella burnetii (Legionellales: Coxiellaceae) Infection Among Wildlife Species and the Tick Hyalomma lusitanicum (Acari: Ixodidae) in a Meso-Mediterranean Ecosystem. JOURNAL OF MEDICAL ENTOMOLOGY. 2020;57(2):551-6.

193. Chisu V, Zobba R, Lecis R, Sotgiu F, Masala G, Foxi C, et al. GroEL typing and phylogeny of Anaplasma species in ticks from domestic and wild vertebrates. Ticks and Tick-borne Diseases. 2018;9(1):31-6.

194. Boularias G, Azzag N, Galon C, Simo L, Boulouis H-J, Moutailler S. High-Throughput Microfluidic Real-Time PCR for the Detection of Multiple Microorganisms in Ixodid Cattle Ticks in Northeast Algeria. PATHOGENS. 2021;10(3).

195. Márquez FJ. Spotted fever group Rickettsia in ticks from southeastern Spain natural parks. Experimental and Applied Acarology. 2008;45(3-4):185-94.

196. Díaz-Sánchez S, Fernández AM, Habela MA, Calero-Bernal R, de Mera IGF, de la Fuente J. Microbial community of Hyalomma lusitanicum is dominated by Francisella-like endosymbiont. Ticks and Tick-borne Diseases. 2021;12(2):101624.

197. Palomar AM, Portillo A, Santibanez S, Garcia-Alvarez L, Munoz-Sanz A, Marquez FJ, et al. Molecular (ticks) and serological (humans) study of Crimean-Congo hemorrhagic fever virus in the Iberian Peninsula, 2013-2015. ENFERMEDADES INFECCIOSAS Y MICROBIOLOGIA CLINICA. 2017;35(6):344-7.

198. Remesar S, Cano-Terriza D, Morrondo P, Jiménez-Ruiz S, López CM, Jiménez-Martín D, et al. Molecular detection of Rickettsia spp. in wild ungulates and their ticks in Mediterranean areas of southwestern Spain. Zoonoses and Public Health. 2023;70(6):485-97.

199. Remesar S, Castro-Scholten S, Cano-Terriza D, Díaz P, Morrondo P, Jiménez-Martín D, et al. Molecular identification of zoonotic Rickettsia species in Ixodidae parasitizing wild lagomorphs from Mediterranean ecosystems. Transboundary and Emerging Diseases. 2022;69(4):e992-e1004.

200. Negredo A, Angel Habela M, Ramirez de Arellano E, Diez F, Lasala F, Lopez P, et al. Survey of Crimean Congo Hemorrhagic Fever Enzootic Focus Spain, 2011-2015. EMERGING INFECTIOUS DISEASES. 2019;25(6):1177-84.

201. Díaz-Cao JM, Adaszek Ł, Dzięgiel B, Paniagua J, Caballero-Gómez J, Winiarczyk S, et al. Prevalence of selected tick-borne pathogens in wild ungulates and ticks in southern Spain. Transboundary and Emerging Diseases. 2022;69(3):1084-94.

202. Torina A, Alongi A, Scimeca S, Vicente J, Caracappa S, de la Fuente J. Prevalence of tick-borne pathogens in ticks in Sicily. Transboundary and Emerging Diseases. 2010;57(1-2):46-8.

203. Rivero-Juarez A, Risalde MA, Gortazar C, Lopez-Lopez P, Barasona JA, Frias M, et al. Detection of Hepatitis E Virus in Hyalomma lusitanicumTicks Feeding on Wild Boars. FRONTIERS IN MICROBIOLOGY. 2021;12:692147.

204. Sánchez M, Valcárcel F, González J, González MG, Martín-Hernández R, Tercero JM, et al. Seasonality of Coxiella Burnetii among Wild Rabbits (Oryctolagus Cuniculus) and the Hyalomma Lusitanicum (Acari: Ixodidae) in a Meso-Mediterranean Ecosystem. Pathogens. 2022;11(1).

205. Pereira A, Parreira R, Cotão AJ, Nunes M, Vieira ML, Azevedo F, et al. Tick-borne bacteria and protozoa detected in ticks collected from domestic animals and wildlife in central and southern Portugal. Ticks and Tick-borne Diseases. 2018;9(2):225-34.

206. Castillo-Contreras R, Magen L, Birtles R, Varela-Castro L, Hall JL, Conejero C, et al. Ticks on wild boar in the metropolitan area of Barcelona (Spain) are infected with spotted fever group rickettsiae. TRANSBOUNDARY AND EMERGING DISEASES. 2022;69(4):E82-E95.

207. Paz Sanchez-Seco M, Jose Sierra M, Estrada-Pena A, Valcarcel F, Molina R, Ramirez de Arellano E, et al. Widespread Detection of Multiple Strains of Crimean-Congo Hemorrhagic Fever Virus in Ticks, Spain. EMERGING INFECTIOUS DISEASES. 2022;28(2):394-402.

208. Islam S, Al-Amin HM, Sazed SA, Islam KM, Bhuiyan MJU, Nath TC, Alam MS. Faunal diversity of ticks in animals and environment in eastern Bangladesh. Veterinary parasitology, regional studies and reports. 2022;31:100736.

209. Osbrink WLA, Showler AT, Abrigo V, de Leon AAP. Rhipicephalus (Boophilus) microplus (Ixodida: Ixodidae ) Larvae Collected From Vegetation in the Coastal Wildlife Corridor of Southern Texas and Research Solutions for Integrated Eradication. JOURNAL OF MEDICAL ENTOMOLOGY. 2020;57(4):1305-9.

210. Nyangiwe N, Yawa M, Qokweni L, Mthi S, Goni S, Mapholi NO. Species richness and adaptation of the invasive cattle tick, Rhipicephalus microplus (Acari: Ixodidae) on camps grazed by sheep in the Eastern Cape Province, South Africa. Ticks and tick-borne diseases. 2023;14(3):102122.

211. Mokhtaria K, Ammar AA, Mohammed Ammar SS, Chahrazed K, Fadela S, Belkacem BT. Survey on species composition of Ixodidae tick infesting cattle in Tiaret (Algeria). Tropical Agriculture. 2018;95(1).

212. Del Valle-Mendoza J, Rojas-Jaimes J, Vasquez-Achaya F, Aguilar-Luis MA, Correa-Nunez G, Silva-Caso W, et al. Molecular identification of Bartonella bacilliformis in ticks collected from two species of wild mammals in Madre de Dios: Peru. BMC research notes. 2018;11(1):405-.

213. Lopez Y, Miranda J, Mattar S, Gonzalez M, Rovnak J. First report of Lihan Tick virus (Phlebovirus, Phenuiviridae) in ticks, Colombia. VIROLOGY JOURNAL. 2020;17(1):63.

214. da Silva JB, da Fonseca AH, Barbosa JD. Molecular characterization of Anaplasma marginale in ticks naturally feeding on buffaloes. INFECTION GENETICS AND EVOLUTION. 2015;35:38-41.

215. Li J, Kelly P, Guo W, Zhang J, Yang Y, Liu W, Wang C. Molecular detection of Rickettsia, Hepatozoon, Ehrlichia and SFTSV in goat ticks. Veterinary Parasitology: Regional Studies and Reports. 2020;20:100407.

216. Bermudez C SE, Felix ML, Dominguez A L, Arauz D, Venzal JM. Molecular screening of tick-borne microorganisms in ticks from rural areas of Panama, with the first record of Ehrlichia minasensis in Rhipicephalus microplus from Central America. Veterinary research communications. 2024;48(2):1301-8.

217. Cleveland CA, Swanepoel L, Box EK, De Nicola A, Yabsley MJ. Rickettsia species in ticks collected from wild pigs (Sus scrofa) and Philippine deer (Rusa marianna) on Guam, Marianna Islands, USA. Acta Tropica. 2019;194:89-92.

218. Moraga-Fernández A, Ruiz-Fons F, Habela MA, Royo-Hernández L, Calero-Bernal R, Gortazar C, et al. Detection of new Crimean–Congo haemorrhagic fever virus genotypes in ticks feeding on deer and wild boar, Spain. Transboundary and Emerging Diseases. 2021;68(3):993-1000.

219. Jimale KA, Zeni V, Ripamonti A, Canale A, Mele M, Benelli G, Otranto D. Grazing system and Hyalomma marginatum tick infestation in cattle with high prevalence of SFG Rickettsia spp. Veterinary Parasitology: Regional Studies and Reports. 2024;49:100994.

220. Ortega N, Arcenillas-Hernández I, Villa M, González M, Caro M. Molecular identification of Borrelia and SFG Rickettsia spp. in hard ticks parasitizing domestic and wild animals in southeastern Spain. Veterinary Research Communications. 2024.

221. Rivera-Paez FA, Labruna MB, Martins TF, Perez JE, Castano-Villa GJ, Ossa-Lopez PA, et al. Contributions to the knowledge of hard ticks (Acari: Ixodidae) in Colombia. TICKS AND TICK-BORNE DISEASES. 2018;9(1):57-66.

222. Jouglin M, Fernandez-de-Mera IG, de la Cotte N, Ruiz-Fons F, Gortazar C, Moreau E, et al. Isolation and characterization of Babesia pecorum sp nov from farmed red deer (Cervus elaphus). VETERINARY RESEARCH. 2014;45.

223. Santos-Silva MM, Melo P, Santos N, Antunes S, Duarte LR, Ferrolho J, et al. PCR screening of tick-borne agents in sensitive conservation areas, Southeast Portugal. Molecular and Cellular Probes. 2017;31:42-5.
